# Homoleptic seven-coordinate Ti(0) and Zr(0) through a new stabilization mode

**DOI:** 10.1039/d6sc00226a

**Published:** 2026-03-12

**Authors:** Ivan Antsiburov, Raphael Bühler, Johannes Stephan, Maxim Erdyakov, Christian Gemel, Samia Kahlal, Olivier Cador, Thierry Guizouarn, Jean-Yves Saillard, Karsten Meyer, Roland A. Fischer

**Affiliations:** a Department of Chemistry, Technical University of Munich, TUM School of Natural Sciences, Chair of Inorganic and Metal-Organic Chemistry Garching Germany; b Catalysis Research Center, Technical University of Munich Garching Germany; c CNRS, Univ Rennes, ISCR-UMR 6226 F-35000 Rennes France; d Department of Chemistry and Pharmacy, Inorganic Chemistry, Friedrich-Alexander-Universität, Erlangen-Nürnberg (FAU) Erlangen 91058 Germany

## Abstract

The stabilization of zero oxidation state group (IV) metal centers in readily accessible compounds on a preparative scale remains a significant challenge. Substituting hydrocarbon ligands in the precursor complexes [Ti(η^6^-toluene)_2_] and [Zr(η^6^-cycloheptatriene)_2_] with monovalent GaTMP (TMP = 2,2,6,6-tetramethylpiperidinyl) yields the first homoleptic seven-coordinate Ti^0^ and Zr^0^ complexes, [Ti(GaTMP)_7_] (1) and [Zr(GaTMP)_7_] (2), which are exclusively coordinated by metalloligands. The bonding situation of 1 and 2 was rationalized through DFT calculations, revealing the critical importance of tangential Ga⋯Ga covalent interactions for stabilizing the compounds. Oxidation of 2 leads to the formation of [Zr(GaTMP)_8_]^2+^ (5), showcasing a similar bonding situation. These Ga⋯Ga interactions arise from significant π-backbonding from the Ti^0^ and Zr^0^ centers into the constructive combinations of the diffuse 4p orbitals of the Ga(i) centers. This unique cooperative feature of the all-Ga metalloligand sphere marks a clear distinction from the bonding properties of formally isolobal carbonyls, phosphines, or N-heterocyclic carbenes.

## Abstract 2

The first homoleptic Ti^0^ and Zr^0^ complexes stabilized by gallylene ligands reveal unprecedented bonding: Ga–Ga interactions enabled by π-backbonding into diffuse Ga 4p orbitals. This unique ligand sphere defies classical carbonyl analogies.

## Graphical abstract

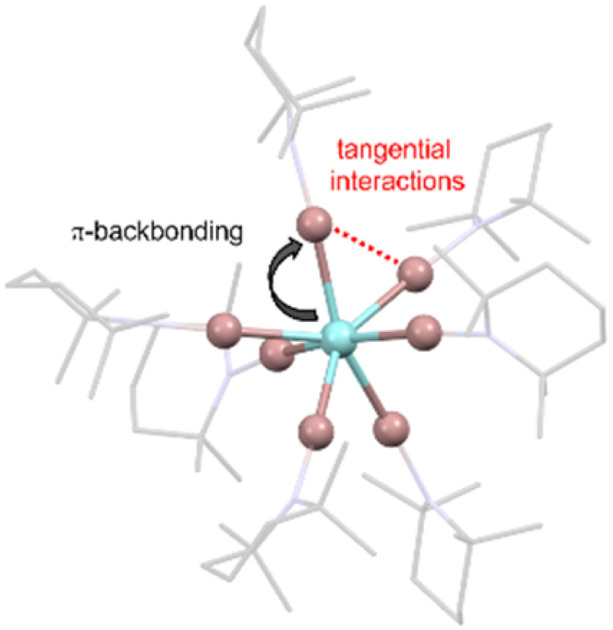

## Introduction

Accessing and stabilizing the highly reactive zero oxidation state of early transition metals remains a frontier in inorganic synthesis.^1–3^ Titanium(0) and zirconium(0) compounds are particularly elusive due to these metals' electropositive and highly oxophilic nature,^1,4^ and the need for a crowded coordination environment to achieve an electronically saturated configuration. This is illustrated by the instability of the classic 18 valence electron complex [Ti(CO)_7_] and the related species [Ti(CO)_*x*_]^*n*^ (*x* ≤ 7, *n* = −1, 0, +1), which, unlike [Cr(CO)_6_], [Fe(CO)_5_], and [Ni(CO)_4_], have only been observed in the gas phase or cryogenic matrices.^5–9^ In the case of the group IV transition metals (TMs), it was found that the formally 20 valence electron (in which TM still fulfils the 18-electron rule^6^) octacoordinated [TM(CO)_8_]^*n*^ (TM = Zr, Hf) are preferred under these conditions, as the CO ligands experience less steric repulsion than in the hepta-coordinated structure; however, they remained inaccessible by wet chemistry.^6^ Among group V metals only vanadium forms a complex V(CO)_6_, which is stable at room temperature, while Ta_2_(CO)_12_ has been isolated more recently^10^ but remains stable only under a carbon monoxide atmosphere. The first successful synthesis of organometallic group IV metal complexes in the zero oxidation state was reported by Cloke, Green, and co-workers.^11^ They used a metal-vapor synthesis approach to prepare bis(η^6^-arene) compounds of group IV metals and their phosphine adducts.^12,13^ Another significant milestone was the development of wet-chemical synthesis of Ti^0^ and Zr^0^ complexes.^14,15^ The first seven-coordinated complexes of group IV metals were prepared by J. E. Ellis and co-workers *via* reduction of [TMCl_4_(thf)_2_] (TM = Ti, Zr) under a CO atmosphere in the presence of tridentate phosphines.^16^ Notably, the highly reduced homoleptic, but merely hexa-coordinated carbonyl metalates [TM(CO)_6_]^2−^ (TM = Ti, Zr, Hf) can be obtained and have been used on a preparative scale.^17–19^ Regardless, and to the best of our knowledge, isolated homoleptic neutral zero oxidation state complexes of group IV metals bearing exclusively monodentate ligands (L = CO, phosphines (PR_3_), N-heterocyclic carbene (NHC), *etc.*) remain elusive. However, for group V metals, the neutral homoleptic complexes of V^0^ and Ta^0^ stabilized by six isocyanide ligands are known.^20,21^

We have previously demonstrated that metalloligands in d-metal complexes of the type [TM(ZnR)_*n*_] (TM = Mo, Ru, Ni, Pd, Pt; R = Cp*, Me, Et) can delocalize electron density across the ZnR ligand sphere, thereby significantly stabilizing highly coordinated structures and energetically compensating for the inherent steric crowding.^22,23^ Furthermore, several examples reported by other research groups demonstrate the ability of metalloligands to access high coordination numbers in transition metals.^24–26^ We hypothesized that this metalloligand approach could be utilized to access the previously elusive group IV homoleptic TM^0^ complexes, where strong π-backbonding interactions also contribute to stabilization – as exemplified by C–C bond-forming processes with early transition metal complexes^27^ and in the aforementioned carbonyl metalates [TM(CO)_6_]^2−^.^28^ To probe this idea, we selected GaTMP (TMP = 2,2,6,6-tetramethylpiperidinyl) as a metalloligand. It is less prone than other Ga^I^R compounds (*e.g.* GaCp*) to the transmetallation of the organic protecting ligand to the transition metal, allowing for the formation of unique gallylene ligated species.^29–31^

Herein, we report the first isolable, homoleptic group IV TM^0^ complexes bearing only monodentate ligands; namely [Ti(GaTMP)_7_] (1) and [Zr(GaTMP)_7_] (2). A detailed bonding analysis provides insight into the electronic structure and reveals the key factors responsible for their unprecedented stability, including significant π-backbonding from the Ti^0^ and Zr^0^ centers into the constructive combinations of the diffuse 4p orbitals of the seven Ga centers.

## Results and discussion

**Scheme 1:**
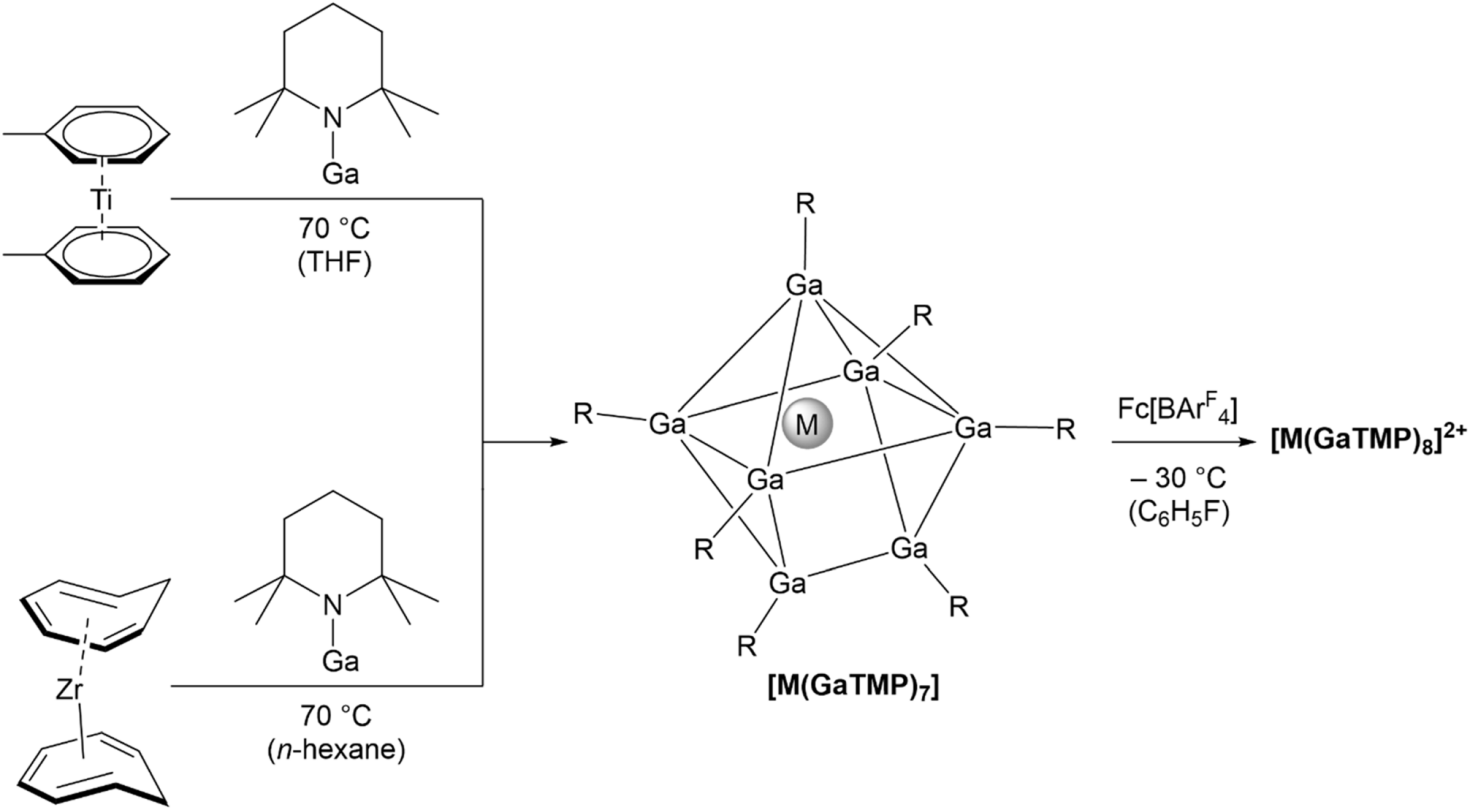
Synthesis of [Ti(GaTMP)_7_] (1) from bis(η^6^-toluene)titanium and [Zr(GaTMP)_7_] (2) from bis(η^6^-cycloheptatriene)zirconium and oxidation towards [Ti(GaTMP)_8_][BAr^F^_4_]_2_ (4) and [Zr(GaTMP)_8_][BAr^F^_4_]_2_ (5).

The reaction of GaTMP (TMP = 2,2,6,6-tetramethylpiperidinyl) with [Ti(η^6^-toluene)_2_] in THF at 70 °C over 10 days gives [Ti(GaTMP)_7_] (1) (Scheme 1) *via* quantitative toluene substitution. Compound 1 was isolated as an analytically pure, red-orange crystalline solid in 17% yield after recrystallization from *n*-hexane. Its heavier analogue [Zr(GaTMP)_7_] (2) can be prepared in a similar fashion from GaTMP and [Zr(η^6^-cycloheptatriene)_2_] in 30% yield. The low yields in both cases can be explained by the formation of metal-precipitates observed during synthesis. Furthermore, the similar solubility of compounds 1 and 2 relative to GaTMP leads to an additional reduction in yield during recrystallization. Compounds 1 and 2 are well soluble in THF, benzene, or hydrocarbons and are highly air sensitive, but stable for months when kept under inert gas.

The identity of compounds 1 and 2 has been unambiguously confirmed by high-resolution mass spectrometry (HRMS), ^1^H- and ^13^C-NMR, ATR-IR/Raman vibrational, and UV-vis electronic absorption spectroscopy. The NMR spectra are consistent with a highly fluxional structure in solution, with all TMP moieties being equivalent in the tested temperature range of −80 °C to room temperature. The ^1^H-NMR resonances of 1 and 2 are slightly downfield-shifted compared to free GaTMP, indicating complexation.

The mass spectra were recorded from a glovebox setup using a liquid injection field desorption ionization (LIFDI) source and an Orbitrap analyzer.^32^ The molecular ions 1^+^ and 2^+^ at *m*/*z* = 1517.4341 [M^+^] (calc. *m*/*z* = 1517.4332) and *m*/*z* = 1559.3866 [M^+^] (calc. *m*/*z* = 1559.3901), respectively, were clearly detected as the base peaks, and the calculated isotope pattern matched the experimental patterns. Additionally, for compound 2, the ligand exchange was studied with *in situ* LIFDI-MS. Therefore, a sample of 2 was heated in toluene at 80 °C with 7 equivalents of GaPMP (PMP = 2,2,4,6,6-pentamethylpiperidinyl), resulting in Δ*m*/*z* = 14 for each substitution (Fig. S31). The substitution proceeds slowly, and a statistical distribution of the products was achieved only after 16 h.

The preparation of [Hf(GaTMP)_7_] (3) was attempted from crude [Hf(η^6^-cycloheptatriene)_2_]. While the molecular ion [Hf(GaTMP)_7_]^+^ could be observed through *in situ* LIFDI-MS at *m*/*z* = 1647.4265 [M^+^] (calc. *m*/*z* = 1647.4311) (Fig. S26), the isolation of 3 was unsuccessful.

**Fig. 1:**
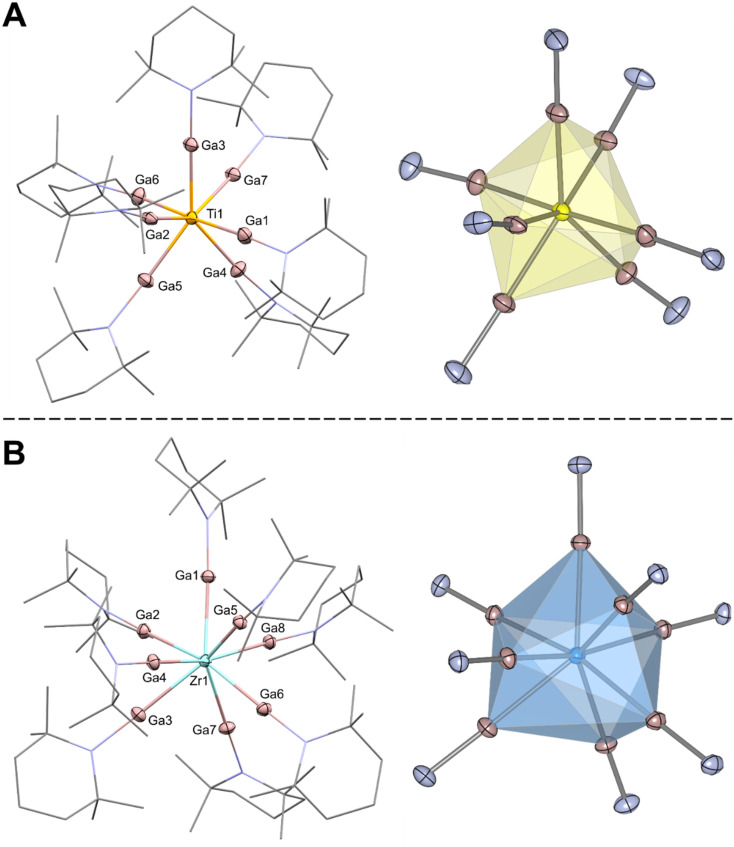
Molecular structures of [Ti(GaTMP)_7_] (1) (A, left) and [Zr(GaTMP)_8_][BAr^F^_4_]_2_ (5) (B, left) in the solid state as determined by SC-XRD. Ti: yellow, Zr: turquois, Ga: magenta, N: blue, and C: grey. H-atoms, cocrystallized solvent molecules and BAr^F^_4_ ions are omitted for clarity. Distorted capped trigonal prism coordination polyhedron calculated from crystal structure data for [Ti(GaTMP)_7_] (1) (A, right) and coordination polyhedron calculated from crystal structure data for [Zr(GaTMP)_8_][BAr^F^_4_]_2_ (5) (B, right). Thermal ellipsoids are shown at the 50% probability level. Relevant bond distances and angles in 1: av. Ti–Ga = 2.428(1) Å, av. Ga–Ga = 2.975(1), av. Ga–N = 1.862(12), and av. Ti–Ga–N = 172.6° ± 2.0°; in 2: av Zr–Ga = 2.667(1) Å, av. Ga–Ga = 3.117(1), av. Ga–N = 1.835(3), and av. Zr–Ga–N = 177.3° ± 1.2°.

Single-crystals of 1 and 2, suitable for X-ray crystallography, were grown from oversaturated *n*-hexane solutions at 30 °C in a Schlenk tube over 3 days. Compounds 1 and 2 crystallize in the groups *Cc* and *P*1̄*,* respectively. The result of the structure refinement for [Ti(GaTMP)_7_] (1) is provided in Fig. 1; the structure of the Zr analogue is given in the crystallography section of the SI. Continuous shape measures^33^ of 1 and 2 indicate that their L_7_ coordination spheres are best described as a distorted mono-capped trigonal prismatic structure (more details in the SI).

The average Ti–Ga distance in 1 is 2.428(1) Å, notably shorter than in the lower-coordinated, heteroleptic titanium(iv)-gallyl complex [Cp*(^*t*^BuN)(py)Ti(GaL*)] at 2.685 Å (L* = (CHNDipp)_2_),^34^ and close to the Ti–Ga distance in [Cp_2_Ti(GaAr*)_2_] at 2.492 Å (Ar* = C_6_H_3_-2,6-(2,4,6-*i*-Pr_3_C_6_H_2_)_2_).^35^ The average Zr–Ga distance in 2 is 2.580(2) Å, shorter than that found in the zirconium(iii)-gallyl complex [Cp_2_Zr(GaL*)_2_]^−^ (2.738 Å)^36^ and very close to the Zr–Ga distance observed for [ZrCp_2_(GaAr*)_2_] (2.635 Å).^35^ The TM–Ga bond distance is expected to shorten within the group of known analogous complexes [Zr(GaTMP)_7_], [Mo(GaTMP)_6_] (average Mo–Ga of 2.385 Å),^29^ and [Ru(GaTMP)_5_] (average Ru–Ga of 2.284 Å)^29^ due to the decreasing covalent radii of the TM and the lowered coordination number, reflecting an overall reduction in steric crowding.

The comparison of structural features among binary solid-state phases indicates that Ti_5_Ga_4_ (ref. 37) exhibits the highest similarity of metal-atom coordination polyhedra to 1, with the unit cell containing a TiGa_7_ motif, among others. In contrast to 1, the coordination environment of the Ti center is more accurately described as a distorted pentagonal bipyramid (see continuous shape measure details in the SI), with Ti–Ga distances ranging from 2.656 to 2.816 Å – significantly longer than those in 1. To the best of our knowledge, no binary Ga-rich solid-state phase containing a ZrGa_7_ motif has been reported to date.

To verify the diamagnetic nature of 1 and 2, as indicated by their NMR behavior, variable-temperature magnetization measurements were conducted on 1 and 2 between 2 K and 300 K (see the SI). These measurements unambiguously confirmed that their electronic ground state is diamagnetic with no unpaired electrons.

The oxidation of 2 with one equivalent of ferrocenium tetrakis[3,5-bis(trifluoromethyl)phenyl]borate (Fc[BAr^F^_4_]) leads to the formation of [Zr(GaTMP)_8_][BAr^F^_4_]_2_ (5). Complex 5 could be crystallized at room temperature from a saturated fluorobenzene solution carefully layered with *n*-hexane. The identity of 5 has been unambiguously confirmed by HRMS spectrometry as well as ^1^H- and ^13^C-NMR, ATR-IR/Raman vibrational, and UV-vis electronic absorption spectroscopy. The ^1^H-NMR resonances of 5 are slightly downfield-shifted compared to the substrate 2, and the integrals of the TMP and [BAr^F^_4_]^− 1^H-resonances are consistent with a dicationic, eight-coordinate Zr-center. LIFDI-MS analysis of 5 further supports this interpretation with the observation of the doubly charged molecular ion [Zr(GaTMP)_8_]^2+^ at *m*/*z* = 885.2262 [M^2+^] (calc. *m*/*z* = 885.2281) and of the single charged {[Zr(GaTMP)_8_][BAr^F^_4_]}^+^ ion at *m*/*z* = 2633.5224 [M^+^] (calc. *m*/*z* = 2633.5216).

Considering the stoichiometry 1 : 1 between 2 and Fc[BAr^F^_4_], one [Zr(GaTMP)_7_] must serve as a ligand donor for another, which is oxidized to [Zr(GaTMP)_8_]^2+^. The conversion of 2 into 5 is also possible when two equivalents of Fc[BAr^F^_4_] and one equivalent of GaTMP ligand are used per equivalent of [Zr(GaTMP)_7_].

Performing the same reaction with 1 leads to the *in situ* LIFDI-MS observation of the analogous [Ti(GaTMP)_8_]^2+^ (4) at *m*/*z* = 863.2499 [M^2+^] (calc. *m*/*z* = 863.2501). Unfortunately, it was not possible to get single crystals of this oily compound despite numerous efforts and different crystallization strategies; however, the analytical data for the raw product are given in the SI.

Single-crystals of 5 suitable for X-ray crystallography were obtained from the carefully layered solution described earlier. The structural refinement of [Zr(GaTMP)_8_]^2+^ (5) unambiguously confirms that the Zr-center adopts an eight-coordinate environment, arranged in an irregular polyhedron. The average Zr–Ga distance in 5 is 2.667(1) Å, which is longer than in neutral 2 and closer to those in [ZrCp_2_(GaAr*)].^35^ This could be explained by the even more crowded coordination sphere of zirconium in 5 compared to 2.

The average Ga–Ga distance in 5 is 3.117(1) Å, noticeably shorter than in the parent compound 2 (3.234(2) Å), which also originates from increasing steric demand around the central atom. The Ga–Ga distances in the coordination shell around the seven-coordinate TM vary between 2.891(1) and 3.017(1) Å in 1 and between 3.005(2) and 3.548(2) Å in 2. For the eight-coordinate 5, the Ga–Ga distances vary between 2.846(1) and 3.584(1) Å. These values are all notably shorter than sum of the van-der-Waals radii (Ga–Ga, 3.74 Å)^38^ and indicate significant interactions between the metalloligands. Nevertheless, the Ga–Ga distances found in 1, 2 and 5 are comparable to those in related complexes [Mo(GaTMP)_6_] (3.120–3.605 Å), [Ru(GaTMP)_5_] (3.065–3.213 Å among basal gallium atoms in the pyramid), and [Ni_2_(GaTMP)_5_] (3.065–3.513 Å).^29,31^

We have previously reported a number of highly coordinated metalloligand complexes featuring weak M–M interactions within the ligand sphere.^22,23,39,40^ These can be rationalized by a lack of available orbitals at the transition metal centers for bonding with the metalloligands, resulting in delocalization among the available bonding atomic orbital (AO) combinations over the ligand sphere. The situation is different for 1, 2, and 3, as the d^4^ Ti^0^, Zr^0^, and Hf^0^ centers each feature seven accepting AOs (s, p, and d) to accommodate the seven gallylene ligand lone pairs. Consequently, the d^2^ metal centers in 4, 5, and in the theoretical [Hf(GaTMP)_8_]^2+^ (6) feature eight accepting orbitals for the eight gallylene ligand lone pairs. Nevertheless, short Ga⋯Ga contacts exist in the single crystal structures of these [TM(GaTMP)_7_] and [Zr(GaTMP)_8_]^2+^ complexes, which prompted an investigation into the nature of these interactions. Covalent interactions between the gallium centers may arise through two possible mechanisms: (i) through-space interactions directly between the gallylene fragments, and (ii) through-bond interactions mediated *via* the transition metal center.^41^

To gain insight into their respective bonding situations, complexes 1–6 were studied using DFT calculations at the PBE0/TZ2P-D3 level (see computational details in the SI). The isolated species 1, 2, and 5 were optimized starting from their experimentally determined structures. The seven-coordinate Hf-analog 3 was optimized starting from the structure of 2, while the eight-coordinate Ti- and Hf-analogs 4 and 6 were optimized starting from the structure of 5. For the sake of conciseness, the bonding situation of these highly-coordinated complexes will be discussed primarily on the basis of the neutral [TM(GaTMP)_7_] (TM = Ti, Zr, Hf) complexes, the findings being also applicable to their dicationic counterparts.

The optimized structures of 1 and 2 (Fig. S47) exhibit similar distorted capped trigonal prism coordination spheres that align well with the experimentally determined solid-state crystal structures, and the computed TM–Ga and Ga–Ga distances correlate well with the experimental data (Table 1). The bonding situations in complexes 1–3 are quite the same, as shown by their various computed indicators (Tables S2 and S3). In particular, the QTAIM and NBO atomic charges (Table S3) are fully consistent across the series. It is of note that in the three complexes, the fairly negative NBO metal charges differ from their positive QTAIM counterparts. Such a discrepancy is associated with an unsymmetrical type of metal–ligand bonding. The simulated UV-vis spectra from the TD-DFT calculations also correspond well to the experimental spectra (Fig. S48 and S49), although they display sharper absorption bands. This is nonetheless expected, as the fluctuating nature of 1 and 2, also observed in the NMR spectra, leads to a broadening of the experimental absorption bands.

**Table 1:** Selected averaged experimental (SC-XRD) and computed (DFT) distances along with the corresponding averaged Wiberg bond indices and QTAIM delocalization indices for [Ti(GaTMP)_7_] (1), [Zr(GaTMP)_7_] (2) and [Zr(GaTMP)_8_]^2+^ (5) as well as the theoretical structures of [Hf(GaTMP)_7_] (3), [Ti(GaTMP)_8_]^2+^ (4) and [Hf(GaTMP)_8_]^2+^ (6)

		Experimental distances	DFT-optimized distances	Wiberg-bond-indices	QTAIM delocalization indices
[Ti(GaTMP)_7_] (1)	Ti–Ga	2.428(1)	2.400	0.597	0.636
	Ga–N	1.862(12)	1.864	0.370	0.749
	Ga⋯Ga	2.975(1)	2.945	0.283	0.350
[Zr(GaTMP)_7_] (2)	Zr–Ga	2.580(2)	2.547	0.552	0.646
	Ga–N	1.822(15)	1.861	0.359	0.759
	Ga⋯Ga	3.252(4)	3.204	0.222	0.276
[Hf(GaTMP)_7_] (3)	Hf–Ga	n/a	2.523	0.579	0.651
	Ga–N	n/a	1.858	0.363	0.761
	Ga⋯Ga	n/a	3.176	0.221	0.286
[Ti(GaTMP)_8_]^2+^ (4)	Ti–Ga	n/a	2.521	0.548	0.525
	Ga–N	n/a	1.835	0.301	0.826
	Ga⋯Ga	n/a	2.819	0.567	0.363
[Zr(GaTMP)_8_]^2+^ (5)	Zr–Ga	2.667(1)	2.637	0.636	0.540
	Ga–N	1.835(11)	1.828	0.397	0.837
	Ga⋯Ga	3.117(1)	2.929	0.459	0.328
[Hf(GaTMP)_8_]^2+^ (6)	Hf–Ga	n/a	2.621	0.538	0.540
	Ga–N	n/a	1.828	0.406	0.837
	Ga⋯Ga	n/a	2.915	0.480	0.333

**Fig. 2:**
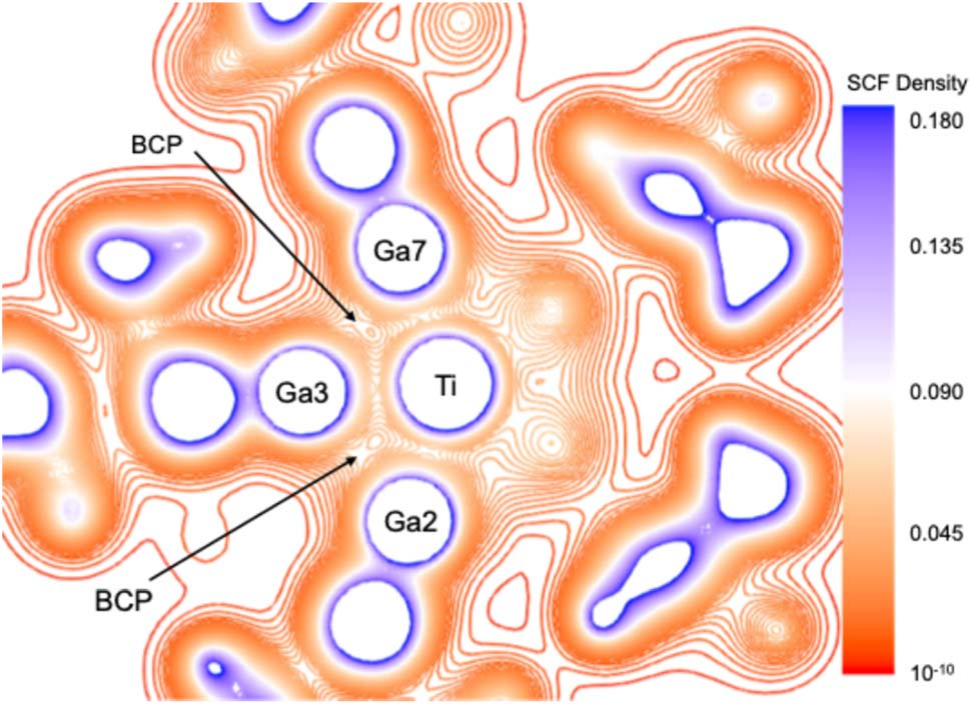
SCF density plot along the Ti–Ga2–Ga3–Ga7 plane extracted from the QTAIM topological analysis, reflecting the Ga^I^–Ga^I^ bond paths along which the Ga^I^–Ga^I^ bond critical points (BCPs) are located.

To evaluate the Ga⋯Ga through-space interactions, an energy decomposition analysis (EDA)^42–44^ was performed on three dimeric (GaTMP)_2_ fragments extracted from the optimized structure of [Ti(GaTMP)_7_], with Ga–Ga distances of 2.837 Å, 2.905 Å, and 3.343 Å. Surprisingly, the total “bonding” energy (TBE) of the dimers was found to be very close to zero (Table S7). A closer examination of the individual energy contributions reveals that, although the (repulsive) total steric interaction (sum of the Pauli repulsion and electrostatic interaction components) varies among the fragments – being highest for the shortest Ga–Ga distance – it is unexpectedly fully offset by the (attractive) orbital interaction, which varies symmetrically, with the dispersion (van der Waals) contribution remaining small. This indicates that the individual GaTMP units within the ligand sphere do not experience strong mutual repulsion or attraction. The non-negligible stabilizing values of the orbital interaction components are associated with the diffuseness of the Ga AOs, allowing significant overlap between the Ga lone pair orbitals of one ligand and the vacant 4p(Ga) AOs of the other, even at distances greater than 3.3 Å. Analyzing the (GaTMP)_7_ ligand shells (L_7_) extracted from the optimized structures of 1, 2, and 3, a topological density analysis within the framework of the Quantum Theory of Atoms in Molecules (QTAIM) formalism^45–47^ led to the identification of 11 Ga⋯Ga bond critical points (BCPs) for the extracted L7 shells (Fig. 2 and Table S6). These indicate non-negligible covalent bonding character between the Ga atoms and are further supported by the Wiberg bond indices and QTAIM delocalization indices (Table S4). This demonstrates that the Ga⋯Ga through-space interactions within the L_7_ ligand spheres play a crucial role in stabilizing the complexes despite the steric bulk of the TMP backbone.

**Fig. 3:**
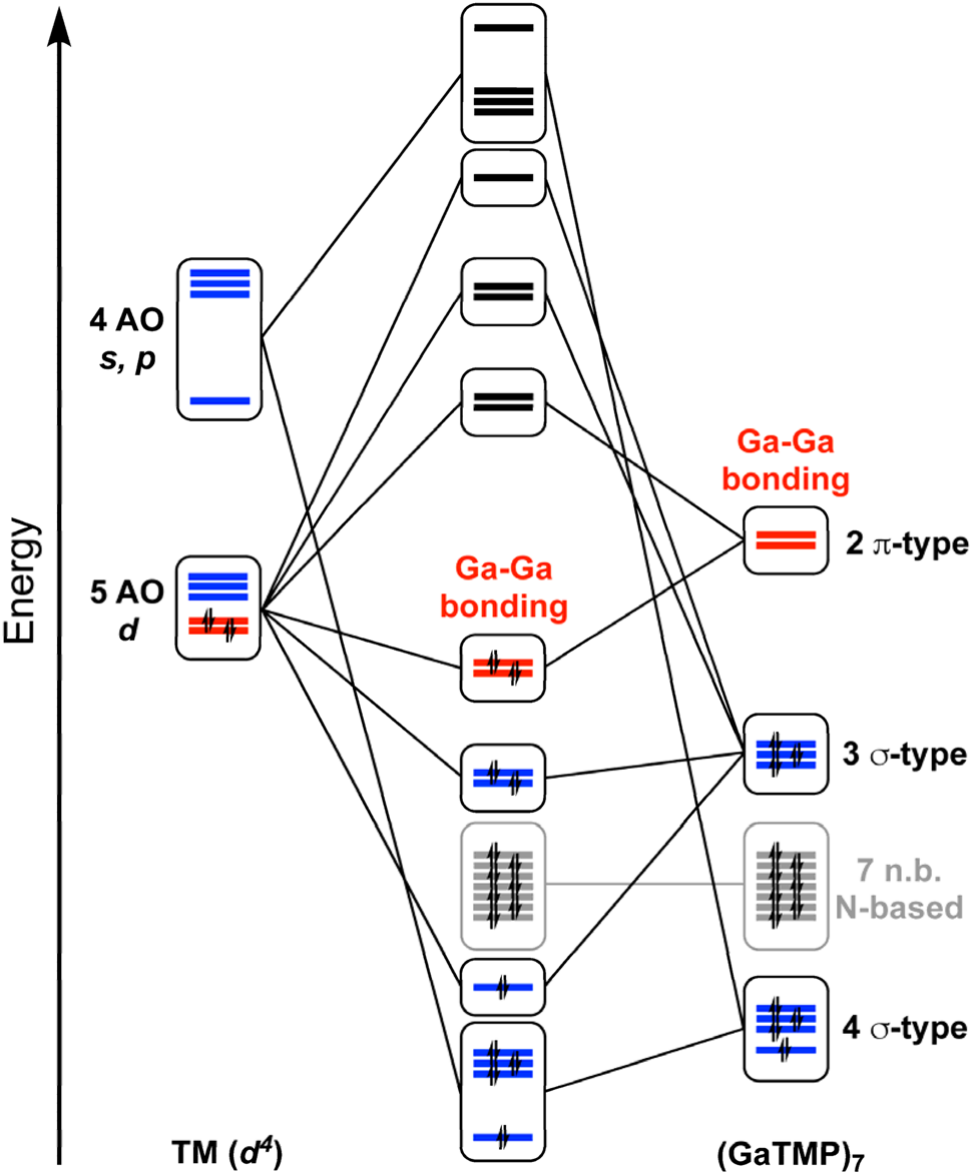
Simplified MO diagram sketching the interactions of the TM (Ti, Zr, and Hf) AOs with the frontier orbitals of the (GaTMP)_7_ ligand shell.

Another crucial contribution to Ga⋯Ga bonding interactions stems from through-bond effects. While a strong interaction from the Ga lone pairs into the vacant TM AOs is observed for both complexes, a significant π-backbonding interaction occurs from the two occupied d-type AOs of the nd^4^-configurated TM into vacant combinations of 4p(Ga) AOs. There are 7 × 2 = 14 such combinations, but those that primarily interact have the lowest energy, thus exhibiting the highest Ga–Ga bonding character. This characteristic is conferred to the complexes' highest occupied molecular orbitals (MOs) (Fig. S54 and S55). This π-backbonding interaction results in total electron populations of 2.61 for the gallium-based 4p combinations in 1, 2.50 in 2, and 2.63 in 3, respectively, demonstrating that π-backbonding plays a crucial role in establishing bonding interactions between the gallylene ligands. A simplified orbital interaction diagram for 1 and 2 is provided in Fig. 3.

Despite the comparable Ga–Ga distances in the related complexes [Mo(GaTMP)_6_], [Ru(GaTMP)_5_] (among basal gallium atoms in the pyramid), and [Ni_2_(GaTMP)_5_], their bonding situation, especially regarding Ga–Ga interactions, revealed by computational methods was rationalized in a more classical way although not contradicting our findings.^29,31^ Nevertheless, for [Ru(GaTMP)_5_] and [Mo(GaTMP)_6_], the π-backbonding was indeed observed and discussed. These orbitals can be interpreted in light of our current findings as being also responsible for Ga–Ga interactions. Reevaluating these known compounds thoroughly in light of our discovery for 1 and 2 will be the subject of an upcoming dedicated manuscript.

EDA and QTAIM analyses of the analogous carbonyl compounds [TM(CO)_7_] (TM = Ti, Zr, Hf; Tables S9 and S10) show a situation similar to that of the gallylene coordinated complexes. The π-backbonding interaction results in a total electron population of 1.98 in the π*(CO) combinations of the (CO)_7_ shell for [Ti(CO)_7_] and 2.02 for [Zr(CO)_7_]. These π*(CO) orbital combinations bond between the carbon atoms of the CO ligands and align well with the findings from Frenking's group.^6,48^ Nevertheless, the π*(CO) orbitals are considerably less diffuse than the accepting 4p orbitals of the gallylene ligands. This is reflected in quasi-negligible C–C Wiberg bond indices and much smaller QTAIM delocalization indices (Table S9) compared to those found in 1, 2, and 3 (Table 1) and thus cannot be described as attractive ligand–ligand interactions.

The bonding analysis of the dicationic eight-coordinate complexes 4, 5, and 6 leads to very similar findings. While more Ga^I^–Ga^I^ BCPs were identified through their topological QTAIM analysis – 10 BCPs for 4 and 9 BCPs for 5 and 6 – their descriptors are consistent with the findings for the neutral seven-coordinate species (Table 2). The same bonding principle of tangential π-backbonding interaction is highlighted by a total electron population in the gallium-based 4p combinations of 1.12 for 4, 5, and 6. The Wiberg bond indices and QTAIM delocalization indices for the Ga⋯Ga interactions are, however, noticeably larger for the dicationic complexes (Table 1), reflecting both the good π-backbonding properties of the group IV TM^2+^ centers and the shorter Ga–Ga distances in 4, 5, and 6 as compared to their neutral homologues.

Averaged topological BCP descriptors for [Ti(GaTMP)_7_] (1), [Zr(GaTMP)_7_] (2) and [Zr(GaTMP)_8_]^2+^ (5) as well as the theoretical structures of [Hf(GaTMP)_7_] (3), [Ti(GaTMP)_8_]^2+^ (4) and [Hf(GaTMP)_8_]^2+^ (6). *ρ*, ∇^2^*ρ*, *H*, *V* and *G* are the electron density, Laplacian of *ρ*, energy density, potential energy density and kinetic energy density values at the BCP, respectively. All values are in a.u

**Table:** 

	[Ti(GaTMP)_7_]^1^			[Zr(GaTMP)_7_]^2^			[Hf(GaTMP)_7_]^3^		
	6 × Ti–Ga	6 × Ga–N	3 × Ga–Ga	6 × Zr–Ga	6 × Ga–N	2 × Ga–Ga	6 × Hf–Ga	6 × Ga–N	0 × Ga–Ga
*ρ*	0.057	0.129	0.041	0.055	0.129	0.038	0.058	0.130	n/a
∇^2^*ρ*	0.1	0.409	0.016	0.085	0.419	0.015	0.091	0.419	n/a
*H*	−0.016	−0.06	−0.013	−0.016	−0.06	−0.011	−0.017	−0.061	n/a
*V*	−0.057	−0.191	−0.03	−0.053	−0.225	−0.026	−0.058	−0.227	n/a
|*V*|/*G*	1.392	1.37	1.766	1.425	1.365	1.751	1.434	1.369	n/a

**Table:** 

	[Ti(GaTMP)_8_]^2+^ (4)			[Zr(GaTMP)_8_]^2+^ (5)			[Hf(GaTMP)_8_]^2+^ (6)		
	8 × Ti–Ga	8 × Ga–N	10 × Ga–Ga	8 × Ti–Ga	8 × Ga–N	9 × Ga–Ga	8 × Ti–Ga	8 × Ga–N	9 × Ga–Ga
*ρ*	0.048	0.137	0.040	0.049	0.139	0.036	0.051	0.139	0.037
∇^2^*ρ*	0.079	0.440	0.009	0.071	0.455	0.013	0.074	0.453	0.013
*H*	−0.012	−0.068	−0.013	−0.013	−0.069	−0.010	−0.014	−0.069	−0.011
*V*	−0.043	−0.246	−0.028	−0.043	−0.252	−0.023	−0.046	−0.252	−0.025
|*V*|/*G*	1.371	1.382	1.847	1.416	1.378	1.757	1.424	1.379	1.760

These findings highlight the significance of the diffuseness of the Ga AOs and the π-backbonding in stabilizing [TM(GaTMP)_7_] and [TM(GaTMP)_8_]^2+^ complexes, revealing the unique bonding characteristics of gallium-rich ligand environments, where the expected ligand–ligand steric repulsion is replaced by a soft attraction.

In an upcoming manuscript, we will expand this approach to homoleptic complexes across the 3d and 4d transition metal series, including a 19 VE complex of manganese, providing further insight into the stabilizing role of metalloligand-based interactions and a better understanding of the fundamental properties of the gallium(i) amide ligand and related systems.

## Conclusions

In summary, we have isolated the first homoleptic Ti^0^ and Zr^0^ complexes exclusively bearing monodentate ligands by substituting the η^6^-hydrocarbon ligand with gallylene donors. In addition, the eight-coordinate Zr^2+^ complex 5 could be obtained through the oxidation of 2 with Fc[BAr^F^_4_] and isolated. The stabilization of these highly coordinated species is enabled by a combination of through-space and through-bond Ga^I^⋯Ga^I^ interactions, the latter being mediated by π-backbonding from the metal centers. These interactions, which substantially overcome steric repulsions between the ligands, are facilitated by the diffuse nature of the gallium 4p orbitals, allowing for effective electron delocalization across the ligand sphere. This bonding scenario, which is not available for the formally isolobal carbonyls, phosphines, or N-heterocyclic carbenes, offers a new strategy for accessing highly reduced early transition metal complexes with structural and bonding features bridging to related sub-nanometer-sized mixed metal clusters and may be extended to other systems, including unprecedented all-Ga metalloligand coordinated lanthanide and actinide complexes, which we will pursue.

## Author contributions

I. A. performed the synthetic work and the characterization of the compounds. R. B. performed the computational bonding analysis. J. S. collected the SC-XRD data, solved and refined the structure. M. E. performed the synthetic work. O. C. and T. G. performed magnetometric measurements. S. K and J.-Y. S. performed computational work and supervised the bonding analysis. C. G. co-supervised the project. K. M. supervised magnetometric measurements. R. A. F developed the idea and supervised the project. All the authors discussed the results. R. B., I. A., C. G., J.-Y. S., K. M. and R. A. F. wrote the manuscript with the input from all authors.

## Conflicts of interest

There are no conflicts to declare.

## Supplementary Material

SC-017-D6SC00226A-s001

SC-017-D6SC00226A-s002

## Data Availability

The code for continuous shape measures can be obtained from the authors on request. All other generated data are available in the supplementary information (SI). Supplementary information: detailed experimental procedures, NMR spectra (Fig. S1–S25), HR-MS (LIFDI-MS) mass spectra (Fig. S26–S31), ATR-IR spectra (Fig. S32–S39), Raman spectra (Fig. S40–S44), UV-vis spectra (Fig. S45–S47), magnetic measurements (Fig. S48), crystallographie (Fig. S49), continuous shape measure (Table S1 and Fig. S50), computational results (Fig. S51–S55; Tables S2–S10). See DOI: https://doi.org/10.1039/d6sc00226a. CCDC 2484315 [Ti(GaTMP)_7_] (1), 2484316 [Zr(GaTMP)_7_] (2) and 2484317 [Zr(GaTMP)_8_][BArF_4_]_2_ (5) contain the supplementary crystallographic data for this paper.^49*a*–*c*^

## References

[cit1] Beaumier E. P., Pearce A. J., See X. Y., Tonks I. A. (2019). Modern applications of low-valent early transition metals in synthesis and catalysis. Nat. Rev. Chem..

[cit2] Eberhardt M. J., Baum M., Clewing S. F., Schubert H., Wesemann L. (2025). Formally Zerovalent Bis(arene) Germylene Complexes of Zirconium and Hafnium. Angew. Chem., Int. Ed..

[cit3] Parkin G. , in Comprehensive Organometallic Chemistry III, ed. M. P. Mingos and R. H. Crabtree, Elsevier, 2007, vol. 1, p. 1–57

[cit4] Kepp K. P. (2016). A Quantitative Scale of Oxophilicity and Thiophilicity. Inorg. Chem..

[cit5] Busby R., Klotzbuecher W., Ozin G. A. (1977). Titanium hexacarbonyl, Ti(CO)6, and titanium hexadinitrogen, Ti(N2)6. 1. Synthesis using titanium atoms and characterization by matrix infrared and ultraviolet-visible spectroscopy. Inorg. Chem..

[cit6] Deng G., Lei S., Pan S., Jin J., Wang G., Zhao L. (2020). *et al.*, Filling a Gap: The Coordinatively Saturated Group 4 Carbonyl Complexes TM(CO)8 (TM=Zr, Hf) and Ti(CO)7. Chem.–Eur. J..

[cit7] Meyer F. (1996). Sequential bond energies of Ti(CO)+ x , x = 1-7. Mol. Phys..

[cit8] Zhou M., Andrews L. (1999). Infrared Spectra and Density Functional Calculations of Small Vanadium and Titanium Carbonyl Molecules and Anions in Solid Neon. J. Phys. Chem. A.

[cit9] Zhou X., Cui J., Li Z. H., Wang G., Liu Z., Zhou M. (2013). Carbonyl Bonding on Oxophilic Metal Centers: Infrared Photodissociation Spectroscopy of Mononuclear and Dinuclear Titanium Carbonyl Cation Complexes. J. Phys. Chem. A.

[cit10] Unkrig W., Schmitt M., Kratzert D., Himmel D., Krossing I. (2020). Synthesis and characterization of crystalline niobium and tantalum carbonyl complexes at room temperature. Nat. Chem..

[cit11] Cloke F. G. N., Green M. L. H. (1979). Synthesis of zerovalent bis-(η-arene)(trimethylphosphine)-hafnium and -zirconium compounds using metal vapours. J. Chem. Soc., Chem. Commun..

[cit12] Cloke F. G. N., Green M. L. H. (1981). Synthesis of zerovalent bis(η-arene) compounds of zirconium, hafnium, niobium, tantalum, and tungsten using the metal vapours. J. Chem. Soc., Dalton Trans..

[cit13] Cloke F. G. N., Lappert M. F., Lawless G. A., Swain A. C. (1987). Synthesis of bis(η-1,3,5-tri-t-butylbenzene) sandwich complexes of titanium, zirconium, and hafnium, and of the hafnium(0) carbonyl complex [Hf(η-But3C6H3)2(CO)]. J. Chem. Soc., Chem. Commun..

[cit14] Bönnemann H., Korall B. (1992). Ether-Soluble Ti0 and Bis(η6-arene)titanium(0) Complexes from the Reduction of TiCl4 with Triethylhydroborate. Angew Chem. Int. Ed. Engl..

[cit15] Green J. C., Green M. L. H., Walker N. M. (1991). Synthesis, crystal structure and reactions of zerovalent 16-electron bis(η-cycloheptatriene)zirconium. J. Chem. Soc., Dalton Trans..

[cit16] Blackburn D. W., Chi K. M., Frerichs S. R., Tinkham M. L., Ellis J. E. (1988). [M(CO)4{CH3C(CH2PMe2)3}], M=Ti, Zr, Hf, Complexes of Zerovalent Titanium, Zirconium, and Hafnium. First Structural Characterization of a Zr0-Carbonyl Complex. Angew Chem. Int. Ed. Engl..

[cit17] Chi K. M., Frerichs S. R., Philson S. B., Ellis J. E. (1987). Hexacarbonylzirconate(2–), [Zr(CO)6]2–: The First Binary Carbonyl Complex of Zirconium. Angew Chem. Int. Ed. Engl..

[cit18] Chi K. M., Frerichs S. R., Philson S. B., Ellis J. E. (1988). Highly reduced organometallics. 23. Synthesis, isolation, and characterization of hexacarbonyltitanate(2-), (Ti(CO)62-). Titanium NMR spectra of carbonyltitanates. J. Am. Chem. Soc..

[cit19] Ellis J. E., Chi K. M. (1990). Highly reduced organometallics. 28. Synthesis, isolation, and characterization of [K(cryptand 2.2.2)]2[Hf(CO)6], the first substance to contain hafnium in a negative oxidation state. Structural characterization of [K(cryptand 2.2.2)]2[M(CO)6].cntdot.pyridine (M = Ti, Zr, and Hf). J. Am. Chem. Soc..

[cit20] Barybin M. V., Young V. G., Ellis J. E. (2000). First Paramagnetic Zerovalent Transition Metal Isocyanides. Syntheses, Structural Characterizations, and Magnetic Properties of Novel Low-Valent Isocyanide Complexes of Vanadium1. J. Am. Chem. Soc..

[cit21] Chakarawet K., Davis-Gilbert Z. W., Harstad S. R., Young Jr V. G., Long J. R., Ellis J. E. (2017). Ta(CNDipp)6: An Isocyanide Analogue of Hexacarbonyltantalum(0). Angew. Chem., Int. Ed..

[cit22] Cadenbach T., Bollermann T., Gemel C., Fernandez I., von Hopffgarten M., Frenking G. (2008). *et al.*, Twelve One-Electron Ligands Coordinating One Metal Center: Structure and Bonding of [Mo(ZnCH3)9(ZnCp*)3]. Angew. Chem., Int. Ed..

[cit23] Cadenbach T., Bollermann T., Gemel C., Tombul M., Fernandez I., Hopffgarten Mv (2009). *et al.*, Molecular Alloys, Linking Organometallics with Intermetallic Hume−Rothery Phases: The Highly Coordinated Transition Metal Compounds [M(ZnR)n] (n ≥ 8) Containing Organo−Zinc Ligands. J. Am. Chem. Soc..

[cit24] Garçon M., Bakewell C., Sackman G. A., White A. J. P., Cooper R. I., Edwards A. J. (2019). *et al.*, A hexagonal planar transition-metal complex. Nature.

[cit25] Hidalgo N., Romero-Pérez C., Maya C., Fernández I., Campos J. (2021). Reactivity of [Pt(PtBu3)2] with Zinc(I/II) Compounds: Bimetallic Adducts, Zn–Zn Bond Cleavage, and Cooperative Reactivity. Organometallics.

[cit26] Boronski J. T., Crumpton A. E., Aldridge S. (2024). A Crystalline NiX6 Complex. J. Am. Chem. Soc..

[cit27] Imabayashi T., Fujiwara Y., Nakao Y., Sato H., Sakaki S. (2005). Theoretical Study of Cp2Zr-, (MeO)2Zr-, and M(PH3)-Mediated Coupling Reactions of Acetylenes (M = Ni, Pt). Significant Differences between Early- and Late-Transition-Metal Complexes. Organometallics.

[cit28] Han J., Grofe A., Gao J. (2021). Variational Energy Decomposition Analysis of Charge-Transfer Interactions between Metals and Ligands in Carbonyl Complexes. Inorg. Chem..

[cit29] Bühler R., Weininger R. J. J., Stephan J., Muhr M., Bock B. M. T., Gemel C. (2024). *et al.*, Homoleptic hexa- and penta-coordinated gallium(i) amide complexes of ruthenium and molybdenum. Dalton Trans..

[cit30] Muhr M., Liang H., Allmendinger L., Bühler R., Napoli F. E., Ukaj D. (2023). *et al.*, Catalytic Alkyne Semihydrogenation with Polyhydride Ni/Ga Clusters. Angew. Chem., Int. Ed..

[cit31] Seifert A., Linti G. (2008). 2,2,6,6-Tetramethylpiperidinogallium as a Terminal and Bridging Ligand in Homo- and Heteroleptic Chromium, Nickel, and Cobalt Complexes. Inorg. Chem..

[cit32] Muhr M., Heiß P., Schütz M., Bühler R., Gemel C., Linden M. H. (2021). *et al.*, Enabling LIFDI-MS measurements of highly air sensitive organometallic compounds: a combined MS/glovebox technique. Dalton Trans..

[cit33] Cirera J., Ruiz E., Alvarez S. (2005). Continuous Shape Measures as a Stereochemical Tool in Organometallic Chemistry. Organometallics.

[cit34] Protchenko A. V., Saleh L. M. A., Vidovic D., Dange D., Jones C., Mountford P. (2010). *et al.*, Contrasting reactivity of anionic boron- and gallium-containing NHC analogues: E–C vs. E–M bond formation (E = B, Ga). Chem. Commun..

[cit35] Yang X.-J., Wang Y., Quillian B., Wei P., Chen Z., Schleyer PvR. (2006). *et al.*, Syntheses, Structures, and Bonding of Cp2M(ER)2 Compounds (Cp = C5H5; M = Ti, Zr; E = Ga, In; R = C6H3-2,6-(2,4,6-i-Pr3C6H2)2). Organometallics.

[cit36] Baker R. J., Jones C., Murphy D. M. (2005). Evidence for the first oxidative insertion of a transition metal into a digallane(4): synthesis, structural characterisation and EPR studies of [Cp2ZrIII{Ga[N(Ar)C(H)]2}2][Li(THF)4], Ar = C6H3Pri2-2,6. Chem. Commun..

[cit37] Schubert K., Meissner H. G., Pötzschke M., Rossteutscher W., Stolz E. (1962). Einige Strukturdaten metallischer Phasen (7). Naturwissenschaften.

[cit38] Bondi A. (1964). van der Waals Volumes and Radii. J. Phys. Chem..

[cit39] Bühler R., Schütz M., Andriani K. F., Quiles M. G., de Mendonça J. P. A., Ocampo-Restrepo V. K. (2025). *et al.*, A living library concept to capture the dynamics and reactivity of mixed-metal clusters for catalysis. Nat. Chem..

[cit40] Schütz M., Muhr M., Freitag K., Gemel C., Kahlal S., Saillard J.-Y. (2020). *et al.*, Contrasting Structure and Bonding of a Copper-Rich and a Zinc-Rich Intermetalloid Cu/Zn Cluster. Inorg. Chem..

[cit41] Hoffmann R. (1971). Interaction of orbitals through space and through bonds. Acc. Chem. Res..

[cit42] Bickelhaupt F. M. and Baerends E. J., in Rev. Comput. Chem., ed. K. B. Lipkowitz, D. B. Boyd, Wiley, New York, 2000, pp. 1–86

[cit43] Morokuma K. (1971). Molecular Orbital Studies of Hydrogen Bonds. III. C=O···H–O Hydrogen Bond in H2CO···H2O and H2CO···2H2O. J. Chem. Phys..

[cit44] Ziegler T., Rauk A. (1979). A theoretical study of the ethylene-metal bond in complexes between copper(1+), silver(1+), gold(1+), platinum(0) or platinum(2+) and ethylene, based on the Hartree-Fock-Slater transition-state method. Inorg. Chem..

[cit45] R. F. W. Bader , Atoms in Molecules-A Quantum Theory, Oxford University Press, Oxford, England, 1990

[cit46] Rodríguez J. I. (2013). An efficient method for computing the QTAIM topology of a scalar field: The electron density case. J. Comput. Chem..

[cit47] Rodríguez J. I., Bader R. F. W., Ayers P. W., Michel C., Götz A. W., Bo C. (2009). A high performance grid-based algorithm for computing QTAIM properties. Chem. Phys. Lett..

[cit48] Frenking G., Fernández I., Holzmann N., Pan S., Krossing I., Zhou M. (2021). Metal–CO Bonding in Mononuclear Transition Metal Carbonyl Complexes. JACS Au.

[cit49] (a) CCDC 2484315: Experimental Crystal Structure Determination, 2026, 10.5517/ccdc.csd.cc2pd46g

