# Supplementary material for: Homoleptic seven-coordinate Ti(0) and Zr(0) through a new stabilization mode
Source: Chem Sci. 2026 Mar 12. Online ahead of print. doi: 10.1039/d6sc00226a (PMC13001666; doi:10.1039/d6sc00226a)
Supplement: Supplementary file 1 [file SC-OLF-D6SC00226A-s001.pdf]

Supplementary Information (SI) for Chemical Science.
This journal is © The Royal Society of Chemistry 2026

Supplementary Information

Homoleptic seven-coordinate Ti(0) and Zr(0) through a new stabilization mode

Ivan Antsiburov+[a,b], Raphael Bühler+[a,b,c], Johannes Stephan[a,b], Maxim Erdyakov[a,b], Christian Gemel[a,b], Samia Kahlal[c], Olivier Cador[c], Thierry Guizouarn[c], Jean-Yves Saillard*[c], Karsten Meyer[d] and Roland A. Fischer*[a,b]

[a]Department of Chemistry, Technical University of Munich, TUM School of Natural Sciences, Chair of Inorganic and Metal-Organic Chemistry, Garching, Germany.
[b]Catalysis Research Center, Technical University of Munich, Garching, Germany
[c]CNRS, Univ Rennes, ISCR-UMR 6226, F-35000 Rennes, France
[d]Department of Chemistry and Pharmacy, Inorganic Chemistry, Friedrich-Alexander-Universität, Erlangen-Nürnberg (FAU), Erlangen, 91058, Germany
*Correspondence: roland.fischer@tum.de; jean-yves.saillard@univ-rennes.fr

+Ivan Antsiburov and Raphael Bühler contributed equally to this work.

Table of Content

1. Materials and methods 3
2. Synthesis of the new compounds 7
3. Synthesis of the starting materials 11
4. NMR Spectra 15
5. LIFDI-MS 29
6. IR Spectra 32
7. RAMAN Spectra 36
8. UV-Vis Spectra 39
9. Magnetic Measurements 41
10. Crystallography 42
11. Continuous Shape Measure 46
12. Computational Results 47
13. References 53

1. Materials and methods

General: Unless stated otherwise, all synthetic manipulations were carried out using standard Schlenk techniques under an atmosphere of argon 4.6 purified by a BTC-catalyst and dried over 3 Å molecular sieves or in a glovebox under an atmosphere of purified argon. All reactions were carried out in standardized *Schlenk* flasks and tubes which were passivated by rinsing with 1,1,1,3,3,3-hexamethyldisilyzane (HMDS), heated with a heat gun to 650 °C for 3 min and allowed to cool to room temperature under dynamic vacuum. Solvents, *i.e.* THF, toluene, *n*-hexane and *n*-pentane, were purified using a *MBraun Solvent Purification System (SPS) MB SPS-800* under argon and stored over dried 3 Å molecular sieves. Fluorobenzene was distilled under argon and stored over dried 3 Å molecular sieves. Deuterated solvents were degassed prior to their use and dried carefully over activated 3 Å molecular sieves. Cycloheptatriene was purchased from *Sigma-Aldrich* and purified by distillation under argon atmosphere. 2,2,6,6-tetramethyl-piperidine was purchased from *Sigma-Aldrich* and purified by distillation under reduced pressure and dried over activated alumina. Potassium hydride was purchased from *Sigma-Aldrich* as a 30% suspension in mineral oil, which was removed by washing with toluene and *n*-hexane and subsequent drying under dynamic vacuum. Ferrocenium [3,5-bis(trifluoromethyl)phenyl]borate was prepared according to literature procedure.[1] All other reagents were purchased from various commercial sources and used as such without further purification.

Analytical Methods: NMR spectra were recorded on a *Bruker AVIII 400 US* (^{1}H, 400 MHz; ^{13}C 101MHz) and Bruker AVHD400 (^{1}H, 400 MHz; ^{13}C 101 MHz, ^{19}F 376 MHz). Chemical shifts are given relative to: TMS for ^{1}H, ^{13}C; $CFCl_3$ for ^{19}F; 15% $BF_3.OEt_2$ in $CDCl_3$ for ^{11}B and were referenced to the residual solvent peak as internal standards. Chemical shifts are reported in parts per million, downfield shifted from TMS, and are consecutively reported as position (δH or δC), relative integral, multiplicity (s = singlet, d = doublet, t = triplet, q = quartet and m = multiplet) and assignment. Variable temperature (VT) NMR spectra were recorded on a *Bruker Avance 400* spectrometer (^{1}H, 400 MHz). FT-IR spectra were measured on an ATR setup with a *Bruker Alpha* FTIR spectrometer under an inert gas atmosphere in a glovebox. Raman spectra were recorded on *Renishaw inVia Reflex Raman System*. The high-resolution mass spectra were obtained using a liquid injection field desorption ionization (LIFDI) from *Linden CMS* as ionization source and a *ThermoFisher Scientific Exactive Plus* Orbitrap as detector. The sample application was performed *via* a fumed silica capillary from a glovebox under an argon atmosphere to enable the measurement of highly air-sensitive compounds.[2] The recorded mass spectra were

evaluated using the *FreeStyle 1.3* program from *ThermoFisher Scientific* and a customized fitting program developed by *Dr. Christian Gemel*. The source code will be made available upon request. No satisfactory elemental analysis data for all compounds were obtained due to the high air sensitivity.

Single-Crystal X-Ray Diffraction and Refinement: Data were collected on a Bruker D8 VENTURE Duo three-angle single crystal x-ray diffractometer equipped with a CPAD detector (Bruker Photon II), an IMS microsource with MoK_{α} radiation (λ = 0.71073 Å) and a Helios optic monochromator, using the APEX4 software package.[3] Measurements were performed on single crystals coated with perfluorinated ether. The crystals were fixed on top of a Kapton micro sampler and frozen under a stream of cold nitrogen. A matrix scan was used to determine the initial lattice parameters. Reflections were corrected for Lorentz and polarization effects, scan speed, and background using SAINT V8.40B.[4] Absorption correction, including odd and even ordered spherical harmonics was performed using the multi-scan method (SADABS 2016/1)[5] for $[Ti(GaTMP)_7]$ (**1**) as well as $[Zr(GaTMP)_8](BAr^F{}_4)_2$ (**5**) and TWINABS 2012/1[6] for the twinned structure of $[Zr(GaTMP)_7]$ (**2**). Space group assignments were based upon systematic absences, E statistics, and successful refinement of the structures. The structures were solved using SHELXT with the aid of successive difference Fourier maps and were refined against all data using SHELXL-2019/1 in conjunction with SHELXLE.[7-9] As compound **2** was twinned, the structure was refined against hklf5 data. Hydrogen atoms were calculated in ideal positions as follows: Methyl hydrogen atoms were refined as part of rigid rotating groups, with a C–H distance of 0.98 Å and $U_{iso(H)} = 1.5 \cdot U_{eq(C)}$. Other H atoms were placed in calculated positions and refined using a riding model, with methylene and aromatic C–H distances of 0.99 Å and 0.95 Å, respectively, other C–H distances of 1.00 Å, all with $U_{iso(H)} = 1.2 \cdot U_{eq(C)}$. Non-hydrogen atoms were refined with anisotropic displacement parameters. Full-matrix least-squares refinements were carried out by minimizing $\Sigma\ w(F_o^2 - F_c^2)^2$ with the SHELXL weighting scheme.[9] Neutral atom scattering factors for all atoms and anomalous dispersion corrections for the non-hydrogen atoms were taken from *International Tables for Crystallography*.[10] A split layer refinement was used for disordered groups and additional restraints on distances, angles and anisotropic displacement parameters were employed to ensure convergence within chemically reasonable limits, if necessary. Whole molecule disorder and rotational disorder of the amide ligand sphere was modelled using the *DSR* tool plugin within SHELXLE.[11] For compound **2**, 0.2 heavily disordered molecules of *n*-hexane as well as one fluorobenzene molecule for compound **5** were treated as a diffuse

contribution to the overall scattering without specific atom positions using the PLATON/SQUEEZE procedure.[12] Images of the crystal structures were created with Mercury (main article)[13] and coordination polyhedral using VESTA 3[14]. CIF files and crystallographic tables were generated using FinalCif.[15] CCDC 2429679–2429681 contain the supplementary crystallographic data for [Ti(GaTMP)$_7$] (**1**), [Zr(GaTMP)$_7$] (**2**) and [Zr(GaTMP)$_8$][BAr$^F{}_4$]$_2$ (**5**). These data can be obtained free of charge via www.ccdc.cam.ac.uk/data_request/cif, or by emailing data_request@ccdc.cam.ac.uk, or by contacting The Cambridge Crystallographic Data Centre, 12 Union Road, Cambridge CB2 1EZ, UK; fax: + 44 1223 336033.

Magnetic Measurements: The magnetic properties of **1** and **2** have been measured from powders sealed in quartz tubes with a *Quantum Design MPMSXL* SQUID magnetometer operating in the temperature range 2-300 K. The sealed tubes were prepared in a glovebox under inert atmosphere. All the data have been corrected from the intrinsic diamagnetism evaluated from Pascal's tables[16] and from the diamagnetism of the sample holder measured separately. Possible contamination with ferromagnetic impurities was verified with magnetization curves recorded at 20 K, 80 K and room temperature.

Magnetism data of powdered sample for **5** (17.9 mg), loaded within a polycarbonate gel capsule inside a plastic straw, was collected on a Quantum Design MPMS-3 SQUID magnetometer. The DC susceptibility was recorded in the temperature range of 2–300 K with an applied DC field of 1 T. Values of the magnetic susceptibility were corrected for core diamagnetism of the sample using tabulated Pascal's constants.[16]

Computational Details: Density Functional Theory (DFT) calculations were performed using the Amsterdam Density Functional code (ADF2020.101)[17] with the ZORA Hamiltonian[18, 19] for the incorporation of scalar relativistic corrections. The triple-ξ Slater basis set plus two polarization functions (STO-TZ2P)[20] was used for all calculations. The geometry optimizations for all complexes were performed using the PBE0 hybrid functional[21-23] and the Becke-Perdew (BP86)[24, 25] GGA exchange-correlation functional, for both with the addition of Grimme's D3 empirical corrections[26] to account for dispersion effects. Frequency calculations, performed on the BP86/TZ2P-D3 optimized geometries only for the sake of computational time, confirmed that the structures correspond to true minima. Further analyses were performed on the PBE0/TZ2P-D3 optimized geometry. Natural atomic orbital (NAO) populations and charges as well as Wiberg bond indices were computed using the natural bond orbital NBO6.0 program[27] implemented in the ADF2020.101 package. Quantum theory of atoms in molecules (QTAIM)[28-30] analysis was

performed as implemented in the ADF2020.101 package. Energy decomposition analysis (EDA) calculations were performed in accordance with the Morokuma-Ziegler formalism.[31-33] For the sake of computational time, time-dependent DFT (TD-DFT) calculations were performed using the Gaussian16 package[34] with the PBE0 functional and the Def2-TZVP basis set.[35, 36]

Continuous Shape Measure[37]: The method of continuous shape measure uses the *N* vertices of an experimentally determined coordination polyhedron normalized and centered in the origin of a three-dimensional cartesian coordinate system as their position vectors Q_i (i = 1,2,3 …, *N*). These are compared to the position vectors P_i (i = 1,2,3, …, *N*) of the *N* vertices of an ideal reference polyhedron that is equally centered and normalized. This is expressed as

$$S_Q(P) = \frac{1}{N} min \sum_{i=1}^{N} \left| \overrightarrow{Q_\iota} - \overrightarrow{P_\iota} \right|^2 \times 100$$

A value of $S_Q(P) = 0$ thus represents the exact ideal shape, with increasing values indicating increasing distortions. This value was calculated from most ideal superimposition, determined by minimizing the distance between the superimposed polyhedral vertices by a numerical rotation algorithm. $S_Q(P)$ thus is the global minimum of these permutations.

In order to identify the cartesian coordinates of two polyhedra with minimized average distances of their vertices, a Java program has been used (Java 15, openjdk-15). The program takes as input the cartesian coordinates of two polyhedra, both centered at the origin. It can then freely rotates, stretch and shrink one of the two polyhedra, finally locating the coordinates with minimized distances between the vertices of the two polyhedra in a recursive process. The code for both programs can be obtained from the authors on request. Continuous shape measures were performed against an idealized mono-capped trigonal prismatic structure as well as two reference compounds (Figure S46): $[(C_2H_4NH_3)_3N]_2[ZrF_7]_2{\cdot}H_2O$[38] (showcasing a mono-capped trigonal prismatic structure of the $[ZrF_7]^{3-}$ anion) and $K_4[V(CN)_7]{\cdot}2H_2O$[39] (where the $[V(CN)_7]^{4-}$ anion shows a pentagonal bipyramidal structure). In general, $S_Q(P) = 0$ indicates no distortion from the reference, $S_Q(P) < 1.00$ indicates minor distortions and $1.00 < S_Q(P) < 3.00$ indicates major distortions from the reference geometry.[37] In the case of **1**, $S_Q(P) = 3.27$ was found when compared to the idealized mono-capped trigonal prism and $S_Q(P) = 2.44$ when compared to the $[ZrF_7]^{3-}$ anion. For **2**, $S_Q(P) = 2.21$ and $S_Q(P) = 1.53$ where found for the comparison with the idealized mono-capped trigonal prism and the $[ZrF_7]^{3-}$ anion respectively. The higher $S_Q(P)$

values for **1** and **2** against the idealized mono-capped trigonal prismatic structure can be explained by its inherent asymmetry with regards to the distances between the vertices and the central metal atom (at the center of mass in the idealized structure). Nevertheless, the continuous shape measures clearly indicate a (highly) distorted mono-capped trigonal prismatic structure for both **1** and **2** as opposed to a pentagonal bipyramidal structure ($S_Q(P)$ = 3.33 and $S_Q(P)$ = 4.53 when compared to the $[V(CN)_7]^{4-}$ anion for **1** and **2** respectively), although the distortion is more pronounced for **1**. In the case of the $TiGa_7$ motif found in the unit cell of the Ti_5Ga_4 phase[40], $S_Q(P)$ = 1.74 was found when comparing it to the pentagonal bipyramidal $[V(CN)_7]^{4-}$ anion, clearly underlying its similar geometry. The results are summarized in Table S1.

2. Synthesis of the new compounds

Synthesis of [Ti(GaTMP)$_7$] (1): GaTMP (1.70 g, 8.08 mmol, 7.5 eq.) and bis(η^6-toluene)titanium (0.25 g, 1.08 mmol, 1.0 eq.) were placed in 100 mL Schlenk tube and dissolved in 20 mL THF. The brown solution was heated at 70 °C for 10 days. The reaction mixture was allowed to cool down to r. t. and filtered. The solvent was removed *in vacuo* and the residue was extracted with *n*-hexane (30 + 5 + 5 mL) and filtered. The resulting red-brown solution was concentrated to 5 mL and crystallized at -78 °C. The crude product separated as a precipitate (543 mg) and was collected by cold filtration and dried under vacuum. Further recrystallisation of the crude product in *n*-hexane (6 mL) at -78 °C afforded 0.270 g analytically pure product as orange microcrystalline powder (0.178 mmol, 17% yield).

^{1}H-NMR (400 MHz, 300 K, benzene-*d*$_6$): δ [ppm] = 1.81 (s, 84H, CH_3), 1.71 – 1.63 (m, 14H, γ-CH_2), 1.45 (t, ^3J = 6.1 Hz, 28H, β-CH_2).

^{13}C{^{1}H}-NMR (101 MHz, 300K, benzene-*d*$_6$): δ [ppm] = 55.55 (*C*Me$_2$), 40.83 (β-*C*H$_2$), 37.14 (*C*H$_3$), 18.91 (γ-*C*H$_2$).

^{13}C-NMR, DEPT-135 (101 MHz, 300K, benzene-*d*$_6$): δ [ppm] = 40.52 (β-*C*H$_2$), 36.83 (*C*H$_3$), 18.59 (γ-*C*H$_2$).

LIFDI-MS [m/z]: found $[M]^+$ 1517.4341 (calculated 1517.4332).

Synthesis of [Zr(GaTMP)$_7$] (2): GaTMP (1.39 g, 6.61 mmol, 7.0 eq.) and bis(η^6-cycloheptatriene)zirconium (0.26 g, 0.94 mmol, 1.0 eq.) were placed in 100 mL Schlenk tube and dissolved in 20 mL *n*-hexane. The brown solution was stirred and heated to 70 °C

in 10 °C/h steps and subsequently stirred for about 4 hours at 70 °C. The reaction solution was allowed to cool down to room temperature and stirred overnight. A red precipitation was observed, and the yellow-red filtrate was removed by Whatman filtration. The remaining red solid was extracted with *n*-hexane (4 x 10 mL) and filtered in order to remove metallic zirconium. The resulting filtrate was dried *in vacuo* providing 0.445 g (0.285 mmol, 30% yield) of product as red microcrystalline powder. ^{1}H-NMR-Analysis reveals a purity higher than 98%, but a small amount of GaTMP remains present. Further recrystallisation of the product in *n*-hexane at -35 °C yielded analytically pure product.

^{1}H-NMR (400 MHz, 300 K, benzene-d_6): δ [ppm] = 1.81 (s, 84H, CH_3), 1.73 – 1.58 (m, 14H, γ-CH_2), 1.44 (t, ^3J = 6.2 Hz, 28H, β-CH_2).

^{13}C{^{1}H}-NMR (101 MHz, 300 K, benzene-d_6): δ [ppm] = 55.79 (*C*Me$_2$), 40.96 (β-*C*H$_2$), 36.95 (*C*H$_3$), 18.95 (γ-*C*H$_2$).

^{13}C-NMR, DEPT-135 (101 MHz, 300 K, benzene-d_6): δ [ppm] = 40.67 (β-*C*H$_2$), 36.66 (*C*H$_3$), 18.65 (γ-*C*H$_2$).

LIFDI-MS [m/z]: found [M]$^+$ 1559.3866 (calculated 1559.3901).

***In situ* generation of [Hf(GaTMP)$_7$] (3):** A red oil, containing bis(η^6-cycloheptatriene)hafnium (10 mg, see details in the Supplementary Information) was dissolved in 1 mL of isoamyl ether and GaTMP (40 mg) were placed in 25 mL Schlenk tube and heated at 120 °C overnight. The reaction mixture was allowed to cool down to room temperature, filtered, diluted with toluene 1 mL and subjected to LIFDI-MS analysis.

LIFDI-MS [m/z]: found [M]$^+$ 1647.4265 (calculated 1647.4311).

Synthesis of [Ti(GaTMP)$_8$](BAr^F)$_2$ (4): A solution of [Ti(GaTMP)$_7$] (**1**) (100 mg, 66 µmol, 1.0 eq.) in fluorobenzene (5 mL) was cooled to -30 °C and solution of ferrocenium [3,5-bis(trifluoromethyl)phenyl]borate (69 mg, 66 µmol, 1.0 eq.) in 3 mL fluorobenzene was added dropwise with a syringe over 15 minutes. The resulting brown reaction mixture was allowed to warm up to room temperature overnight. Then it was filtered and the filtrate was layered with *n*-hexane (20 mL). After a week a dark oil was formed in a Schlenk tube, it was separated from supernatant, washed with *n*-hexane (2×5 mL) and dried *in vacuo* providing 20 mg (6 µmol, 18% yield) of product as dark brown powder. All attempts to obtain single crystals suitable for XRD-analysis by carefully layering with *n*-hexane or toluene of solutions containing **4** in fluorobenzene or THF were not successful.

^{1}H-NMR (400 MHz, 300 K, THF-d_8): δ [ppm] = 7.79 (m, 16H, BAr^F), 7.57 (s, 8H, BAr^F) 1.79 (s, 96H, CH_3) 1.70 – 1.59 (m, 16H, γ-CH_2), 1.55 (t, ^3J = 6.0 Hz, 32H, β-CH_2).

^{13}C{^{1}H}-NMR (101 MHz, 300 K, THF-d_8) δ [ppm] = 163.00 (q, J = 49.7 Hz, BAr^F), 135.78, 130.16 (q, J = 31.2 Hz, BAr^F), 125.70 (q, J = 272.2 Hz, BAr^F), 118.48 – 118.23 (m, BAr^F), 60.80 (CMe$_2$), 39.57 (β-CH$_2$), 37.27 (CH$_3$), 19.27 (γ-CH$_2$).

^{13}C-NMR, DEPT-135 (101 MHz, 300 K, THF-d_8) δ [ppm] = 134.61 (BAr^F), 117.20 (BAr^F), 38.34 (β-CH$_2$), 36.11 (CH$_3$), 18.11 (γ-CH$_2$).

^{19}F-NMR (376 MHz, THF-d_8) δ [ppm] = -63.41.

^{11}B-NMR (128 MHz, THF-d_8) δ [ppm] = -6.50.

LIFDI-MS [m/z]: found [Ti(GaTMP)$_8$]$^{2+}$ 863.2499 (calculated 863.2501).

Synthesis of [Zr(GaTMP)$_8$](BAr^F)$_2$ (5) – method I: A solution of [Zr(GaTMP)$_7$] (**2**) (100 mg, 64 µmol, 1.0 eq.) in fluorobenzene (5 mL) was cooled to -30 °C and solution of ferrocenium [3,5-bis(trifluoromethyl)phenyl]borate (67 mg, 64 µmol, 1.0 eq.) in 3 mL fluorobenzene was added dropwise with a syringe over 15 minutes. The resulting brown reaction mixture was allowed to warm up to room temperature overnight. Then it was filtered and the filtrate was layered with *n*-hexane (20 mL) and after 10 days, dark crystals suitable for SC-XRD analysis were obtained. Those were isolated by filtration, washed with *n*-hexane (2×5 mL) and dried *in vacuo* providing 74 mg (21 µmol, 33% yield) of product as dark crystals.

^{1}H-NMR (400 MHz, 300 K, THF-d_8): δ [ppm] = 7.79 (m, 16H, BAr^F), 7.57 (s, 8H, BAr^F) 1.75 (s, 96H, CH_3) 1.71 – 1.58 (m, 16H, γ-CH_2), 1.54 (t, ^3J = 6.0 Hz, 32H, β-CH_2).

^{13}C{^{1}H}-NMR (101 MHz, 300 K, THF-d_8) δ [ppm] = 163.00 (q, J = 49.7 Hz, BAr^F), 135.78, 130.16 (q, J = 31.7 Hz, BAr^F), 125.70 (q, J = 272.7 Hz, BAr^F), 118.48 – 118.23 (m, BAr^F), 60.83 (CMe$_2$), 39.72 (β-CH$_2$), 36.83 (CH$_3$), 18.40 (γ-CH$_2$).

^{13}C-NMR, DEPT-135 (101 MHz, 300 K, THF-d_8) δ [ppm] = 134.62 (BAr^F), 117.19 (BAr^F), 38.56 (β-CH$_2$), 35.67(CH$_3$), 17.24 (γ-CH$_2$).

^{19}F-NMR (376 MHz, THF-d_8) δ [ppm] = -63.36.

^{11}B-NMR (128 MHz, THF-d_8) δ [ppm] = -6.50.

LIFDI-MS [m/z]: found [Zr(GaTMP)$_8$]$^{2+}$ 885.2262 (calculated 885.2281).

Synthesis of [Zr(GaTMP)$_8$](BAr^F)$_2$ (5) – method II: A solution of [Zr(GaTMP)$_7$] (**2**) (50 mg, 32 µmol, 1.0 eq.) and GaTMP (6.7 mg, 32 µmol, 1.0 eq.) in fluorobenzene (2 mL) was cooled to -30 °C and solution of ferrocenium [3,5-bis(trifluoromethyl)phenyl]borate (67 mg,

64 μmol, 2.0 eq.) in 3 mL fluorobenzene was added dropwise with a syringe over 15 minutes. The resulting brown reaction mixture was allowed to warm up to room temperature overnight. Then it was filtered and the filtrate was layered with *n*-hexane (20 mL) and after 10 days, large dark crystals were obtained. Those were isolated by filtration, washed with *n*-pentane (2×10 mL) and dried *in vacuo* providing 86 mg (25 μmol, 77% yield) of product as dark crystals.

3. Synthesis of the starting materials

Synthesis of Gallium tetramethylpiperidide

GaTMP was synthesized according to a modified literature procedure.[41] GaCp* (6.00 g, 29.3 mmol, 1.0 eq.) was dissolved in THF (15 mL) in a glovebox, transferred to a 500 mL Schlenk flask, and diluted with additional THF (80 mL). Using a dropping funnel, LiTMP (4.74 g, 32.2 mmol, 1.1 eq.), obtained from TMPH and *n*-BuLi according to a literature procedure.[42] was slowly added to the reaction mixture cooled to -37 °C over two hours. After stirring the brown suspension at r.t. overnight, the solvent and volatiles were removed under vacuum at 30 °C. The crude product was extracted with *n*-hexane (250 mL) and filtered through a D4 frit. The filtrate was concentrated to 15 mL, transferred into a Schlenk tube and crystallized overnight at -78 °C. The solid was collected by cold filtration and then dissolved in *n*-pentane (15 mL) at r. t. and recrystallized overnight at -78°C. Then the cold filtration was performed again, and the collected crude product was dried in *vacuo*, firstly, allowed to warm up till r. t. and secondly heated at 70°C under vacuum for 2 hours to get rid of rest of GaCp* by evaporation which collects on the cold top of the Schlenk tube. The product was obtained as an analytically pure, light brown/dark orange powder (3.41 g, 16.2 mmol, 55%).

^{1}H-NMR (400 MHz, 300 K, benzene-*d*$_6$): δ [ppm] = 1.67 (s, 12H, CH_3), 1.61 – 1.53 (m, 2H, γ-CH_2), 1.33 (t, ^3J = 6.4 Hz, 4H, β-CH_2).

Synthesis of GaPMP

GaPMP was synthesized analogously to GaTMP, as described above. LiPMP was prepared according to literature procedure.[43] A solution of 2.75 g GaCp* (13.34 mmol, 1.00 eq.) in THF (50 mL) was cooled -78 °C, subsequently, a solution of 2.17 g (13.34 mmol, 1.00 eq.) LiPMP in THF (40 mL) was added dropwise over 30 minutes under continuous stirring. The reaction mixture was stirred overnight, allowing it to slowly warm up to r.t. The resulting dark brown solution containing a white precipitate was then dried in vacuo and the solid residue was extracted with n-hexane (3 x 35 mL) *via* cannula filtration. An attempt to recrystallize the product from hexane was unsuccessful due to high solubility, instead the residual GaCp* was largely removed by heating the sample to 70 °C in a water bath *in vacuo*. The product of sufficient purity was obtained as a dark brown crystalline solid (1.98 g, 8.80 mmol, 66.0%).

^{1}**H-NMR** (400 MHz, 300 K, benzene-d_6): δ [ppm] = 1.85 (s, 6H, C(C*H*$_3$)$_2$), 1.80–1.71 (m, 1H, γ-C*H*), 1.54 (s, 6H, C(C*H*$_3$)$_2$), 1.35 (dd, J_{gem} = 12.8, ^3J = 3.4 Hz, 2H, equatorial CH*H*), 0.91 (t, 2H, J_{gem} = ^3J = 12.8 Hz, 2H, axial CH*H*), 0.84 (d, ^3J = 6.4 Hz, 3H, γ-CH$_3$).

^{13}**C{^{1}H}-NMR** (101 MHz, 300 K, benzene-d_6) δ [ppm] = 56.34 *C*(CH$_3$)$_2$, 50.34(β-*C*H$_2$), 39.30 C(*C*H$_3$)$_2$, 34.65 C(*C*H$_3$)$_2$, 24.18 CH*C*H$_3$, 23.18 (γ-*C*H$_2$).

Synthesis of potassium triethylborohydride

Potassium triethylborohydride was synthesized according to a modified literature procedure.[44] Potassium hydride powder (3.56 g, 88.8 mmol, 1.25 eq.) was suspended in 70 mL of toluene. Triethylborane (10 mL, 71.0 mmol, 1.00 eq.) was then added dropwise (very exotherm reaction!) and the reaction mixture was stirred at r.t. for 2 hours. The resulting colorless, clear solution was separated from the precipitate *via* cannula filtration. The concentration of potassium triethylborohydride in toluene was determined by acid-base titration. A 0.5 mL aliquot of the product solution was quenched with 6 mL of water and titrated with 0.1 M HCl. Based on this analysis, the concentration of the product in toluene was calculated to be 0.94 M, with a total volume of 70 mL (65.8 mmol, 93%).

Synthesis of Bis(η^6-toluene)titanium

Bis(η^6-toluene)titanium was synthesized according to a literature procedure.[45] Titanium tetrachloride (3.3 mL, 30.1 mmol, 1.00 eq.) was dissolved in 75 mL of toluene. A 0.5 M solution of potassium triethylborohydride in toluene (241 mL, 120.5 mmol, 4.00 eq.) was added dropwise to the titanium tetrachloride solution over 2 hours in an ultrasonic bath (*Sonorex* Fa. *Bandelin*) at r.t. During the addition, a black precipitate formed with a release of hydrogen gas. The reaction mixture was further sonicated for 1 hour until gas release ended. The black precipitate was separated from the dark red filtrate using a D4 frit and extracted with toluene (3×40 mL). The toluene was removed under high vacuum and the residue was extracted with *n*-pentane (5×100 mL). The black precipitate was separated from the dark red extracts by using a D4 frit, and the combined filtrates were concentrated to 20 mL under dynamic vacuum and crystallized at –78 °C. The obtained dark red precipitate was separated by cold filtration from the mother liquor and dried under vacuum, yielding bis(η^6-toluene)titanium as dark red plates (720 mg, 3.10 mmol, 10%).

^{1}**H-NMR** (400 MHz, 300 K, benzene-d_6): δ [ppm] = 5.00 – 4.81 (m, 10H, C*H*), 2.02 (s, 6H,

CH_3).

$^{13}C\{^1H\}$-NMR (101 MHz, 300 K, benzene-d_6) δ [ppm] = 94.45 (CCH_3), 82.40 (*m*-CH), 81.42 (*p*-CH), 79.60 (*o*-CH), 22.34 (CH_3).

Synthesis of Bis(η^6-cycloheptatriene)zirconium

Bis(η^6-cycloheptatriene)zirconium was synthesised according to a literature procedure.[46] Sodium (1.00 g, 43.5 mmol, 5.1 eq.) cut into small pieces was added slowly to mercury (10 ml, 675.5 mmol, 78.7 eq.) at 0 °C in a Schlenk tube and stirred manually with a glass rod. The amalgamation process was completed by putting the tube into ultrasonic bath (*Sonorex* Fa. *Bandelin*). Zirconium (IV) chloride (2.00 g, 8.58 mmol, 1.0 eq.) was suspended in cold THF (70 mL) at –78 °C. After stirring for 1 hour, the orange suspension was added to the pre-cooled (–78 °C) Na/Hg amalgam and vessel was rinsed with THF (15 mL) to ensure complete transfer. (Note: A large, strong magnetic stir bar is essential to ensure effective stirring at the Na/Hg interface.) After stirring for 15 minutes, cycloheptatriene (5.00 mL, 48.2 mmol, 5.6 eq.) was added. The reaction mixture allowed to warm up gradually to +10 °C over 3 hours, then the solvent was removed *in vacuo* without heating above + 10 °C. The crude product was extracted at room temperature with *n*-pentane (6 x 60 mL) using the ultrasonic bath and was filtered through a celite pad. The red-brown filtrate was concentrated to one-third of its volume, canula filtered to remove residual mercury and solids, and stored overnight at –78 °C to crystallize. The obtained dark microcrystalline precipitate was isolated by cold filtration and dried *in vacuo*, yielding bis(η^6-cycloheptatriene) zirconium as black powder (0.369 g, 57.0 mmol, 16%).

^{1}H-NMR (400 MHz, 300 K, benzene-d_6): δ [ppm] = 5.99 – 5.80 (m, 4H, CH), 5.52 (td, J = 10.2, 2.1 Hz, 2H, CH), 5.13 – 4.99 (m, 2H, CH), 4.80 – 4.68 (m, 2H, CH), 3.13 – 3.03 (m, 2H, CH), 2.71 (dt, J = 12.3, 7.9 Hz, 2H, CH_2), 1.30 (dt, J = 12.1, 1.8 Hz, 2H, CH_2).

$^{13}C\{^1H\}$-NMR (101 MHz, 300 K, benzene-d_6): δ [ppm] = 119.94 (CH), 119.29 (CH), 98.21 (CH), 96.93 (CH), 77.58 (CH), 67.20 (CH), 32.29 (CH_2).

Synthesis of Bis(η^6-cycloheptatriene)hafnium

Bis(η^6-cycloheptatriene)hafnium was synthesised according to a modified literature procedure.[46] Hafnium (IV) chloride (2.00 g, 6.24 mmol, 1.0 eq.) was suspended in THF (50 mL) and cooled to –78 °C, then a cold suspension of KC_8 (4.31 g, 31.85 mmol, 5.1 eq.)

in THF (20 mL) was added *via* canula and the Schlenk tube was rinsed with THF (2×10 mL) to ensure complete transfer. After stirring for 15 minutes, cycloheptatriene (3.63 mL, 35.0 mmol, 5.6 eq.) was added. The reaction mixture allowed to warm up gradually to +10 °C over 3 hours, then the solvent was removed *in vacuo* without heating above + 10 °C. The crude product was extracted at room temperature with toluene (3 x 60 mL) and was filtered through a celite pad. The solvent was removed *in vacuo* providing a red oil (247 mg), which contains bis(η^6-cycloheptatriene) hafnium according to LIFDI-MS analysis.

LIFDI-MS [m/z]: found $[Hf(C_7H_8)_2]^+$ 364.0704 (calculated 364.0712); found $[Hf(C_7H_8)_3]^+$ 456.1329 (calculated 456.1338); found $[Hf(C_7H_8)_4]^+$ 548.1965 (calculated 548.1964).

4. NMR Spectra

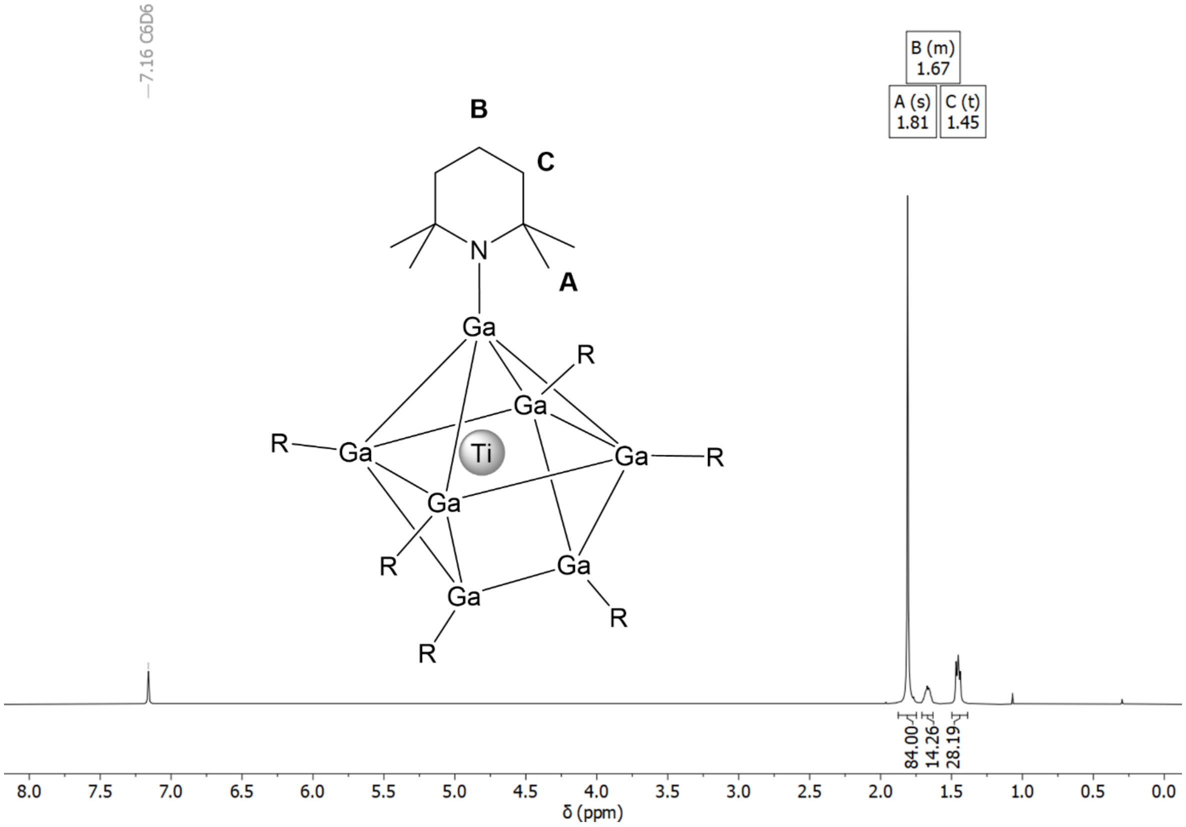

Figure S1: ^{1}H-NMR (benzene-d_6) spectrum of $[Ti(GaTMP)_7]$ (**1**).

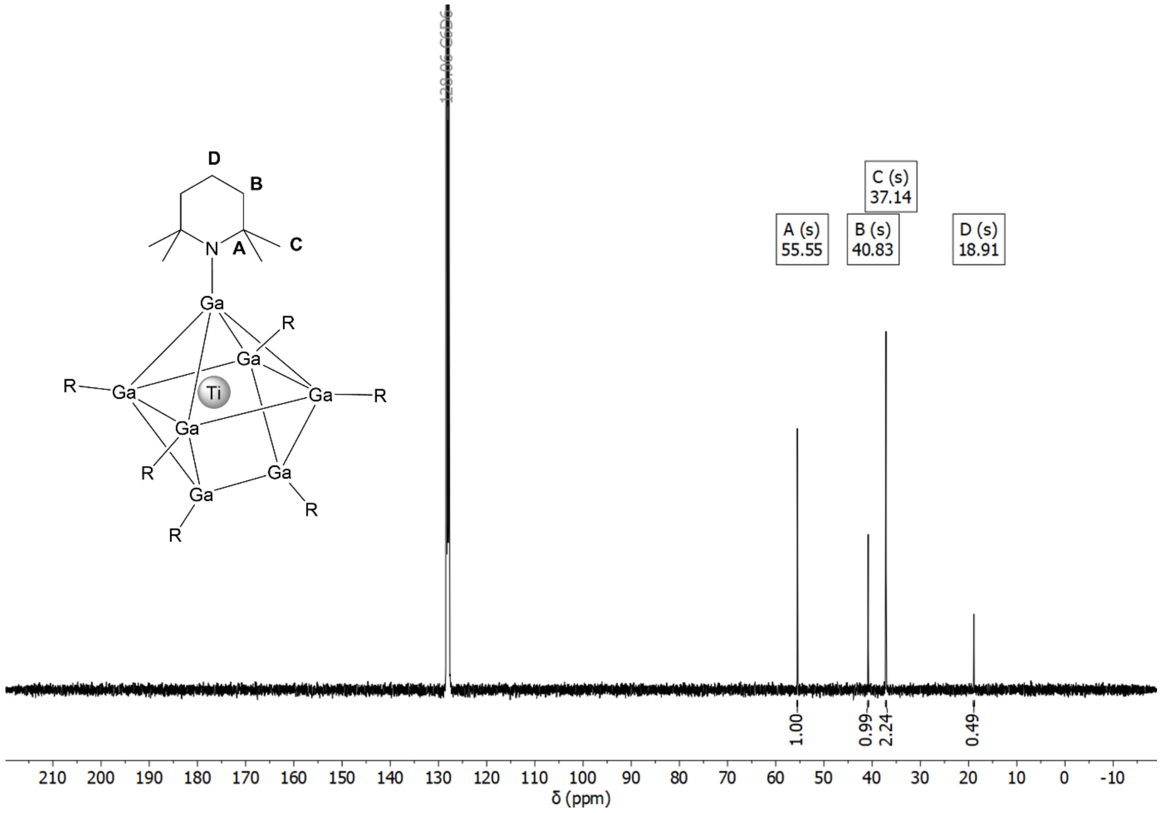

Figure S2: ^{13}C{^{1}H}-NMR (benzene-d_6) spectrum of $[Ti(GaTMP)_7]$ (**1**).

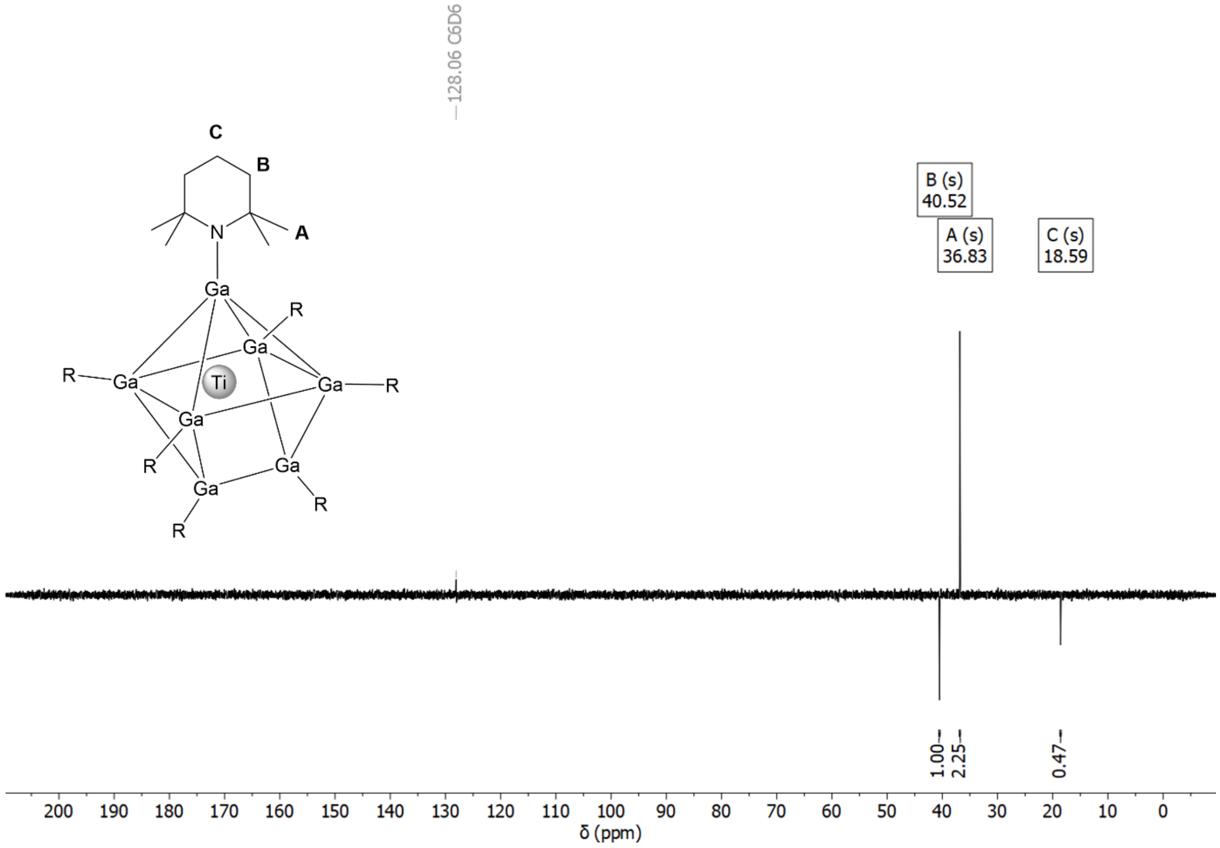

Figure S3: ^{13}C DEPT-135 NMR (benzene-d_6) spectrum of [Ti(GaTMP)$_7$] (**1**).

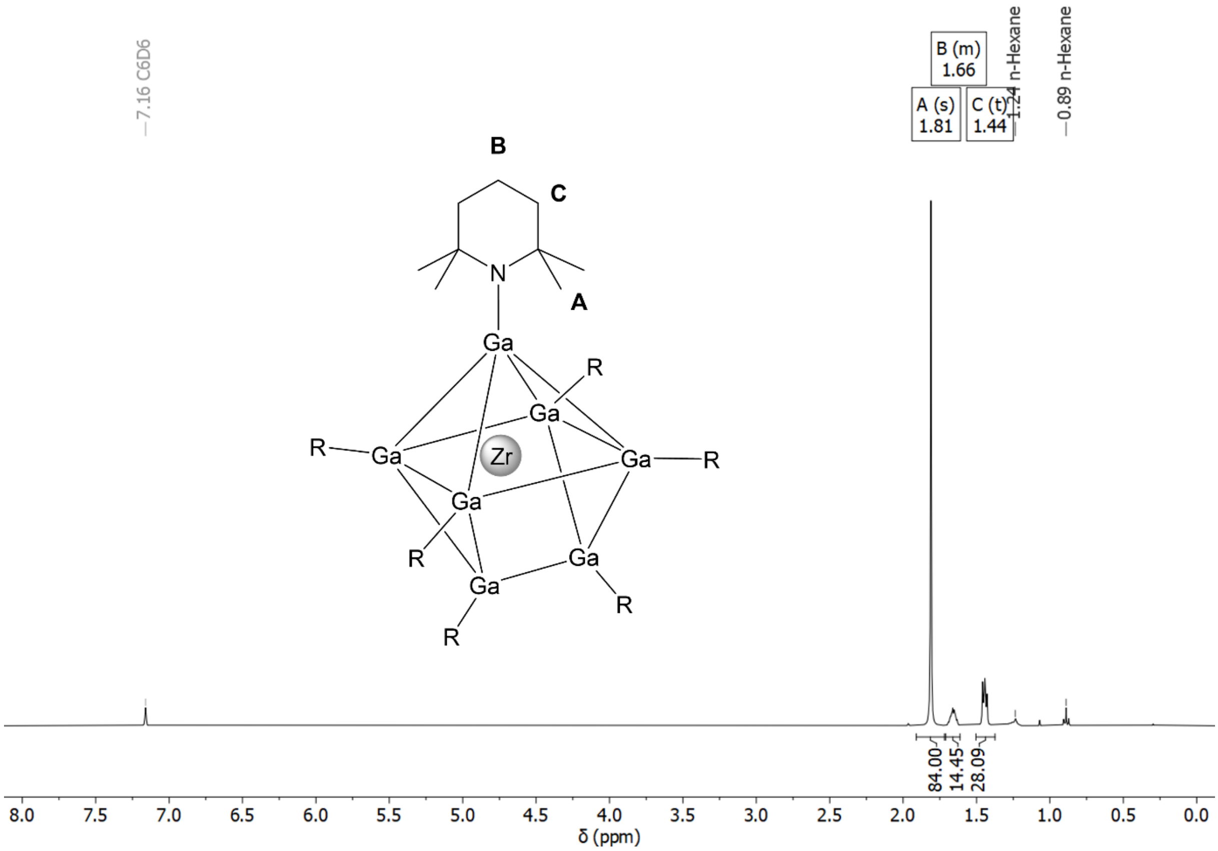

Figure S4: ^{1}H-NMR (benzene-d_6) spectrum of [Zr(GaTMP)$_7$] (**2**).

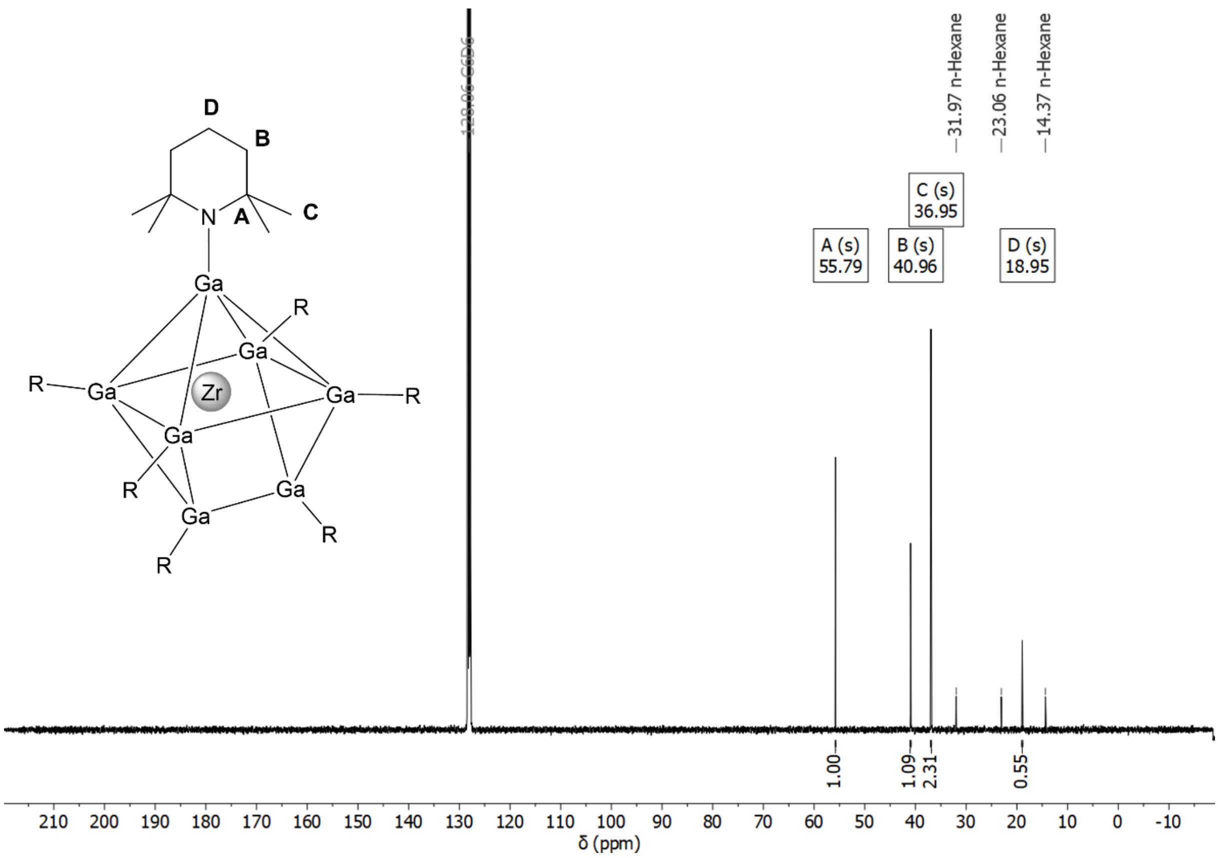

Figure S5: $^{13}C\{^{1}H\}$-NMR (benzene-d_6) spectrum of [Zr(GaTMP)$_7$] (**2**).

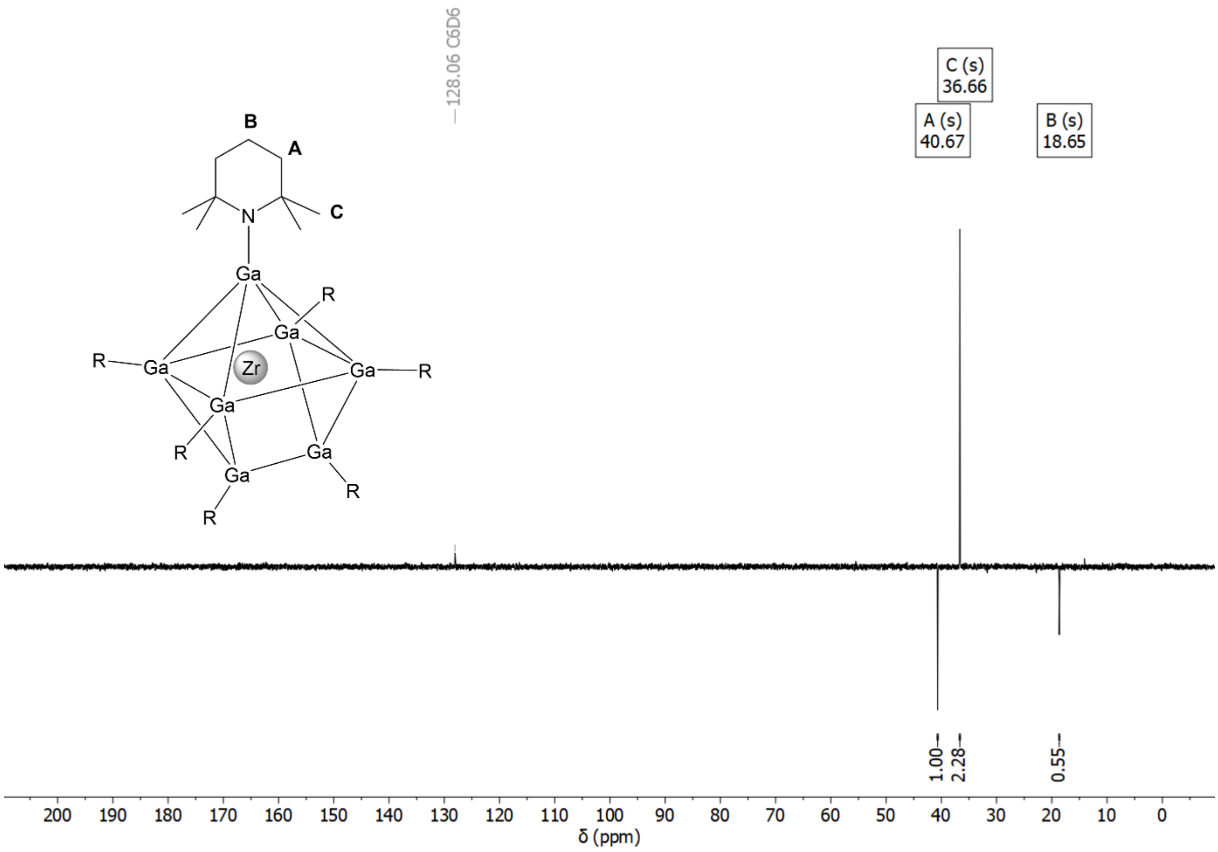

Figure S6: ^{13}C DEPT 135-NMR (benzene-d_6) spectrum of [Zr(GaTMP)$_7$] (**2**).

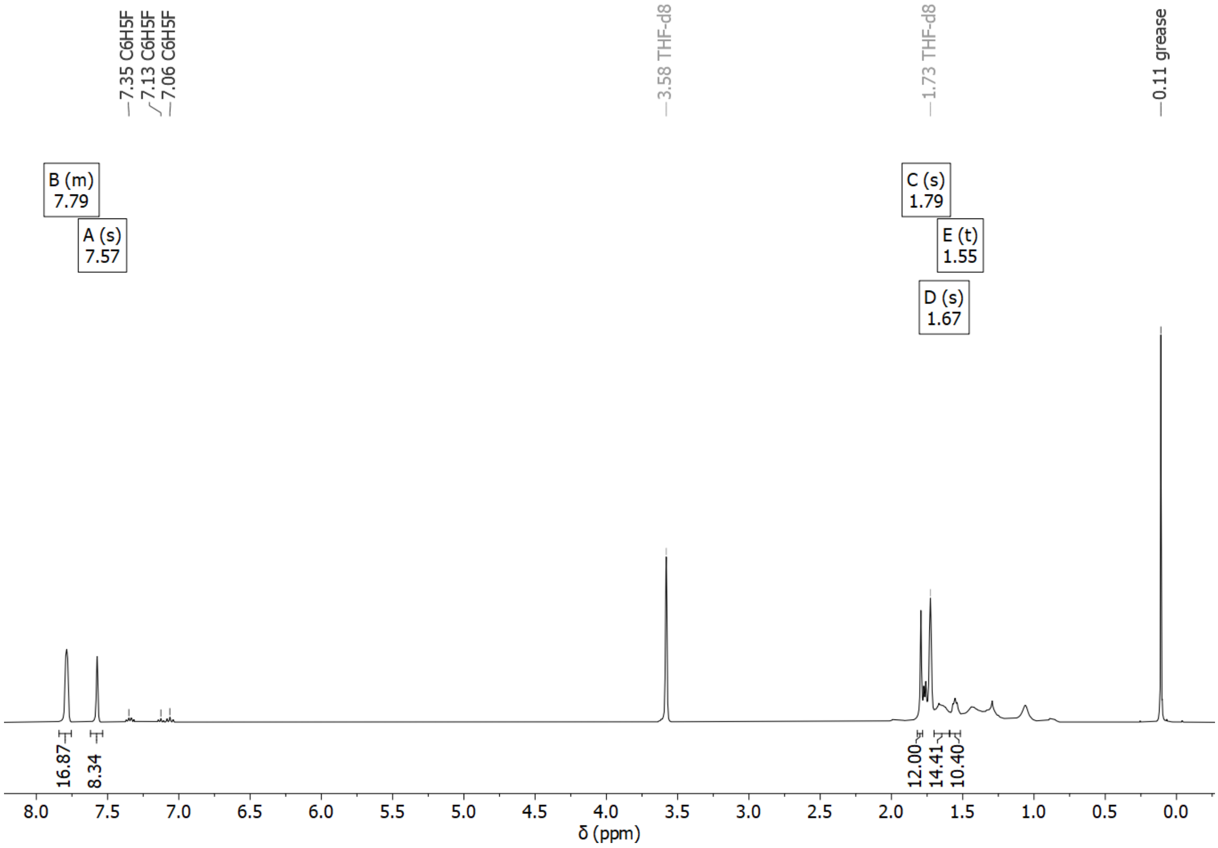

Figure S7: ^{1}H-NMR (THF-d_8) spectrum of $[Ti(GaTMP)_8][BAr^F{}_4]_2$ (**4**).

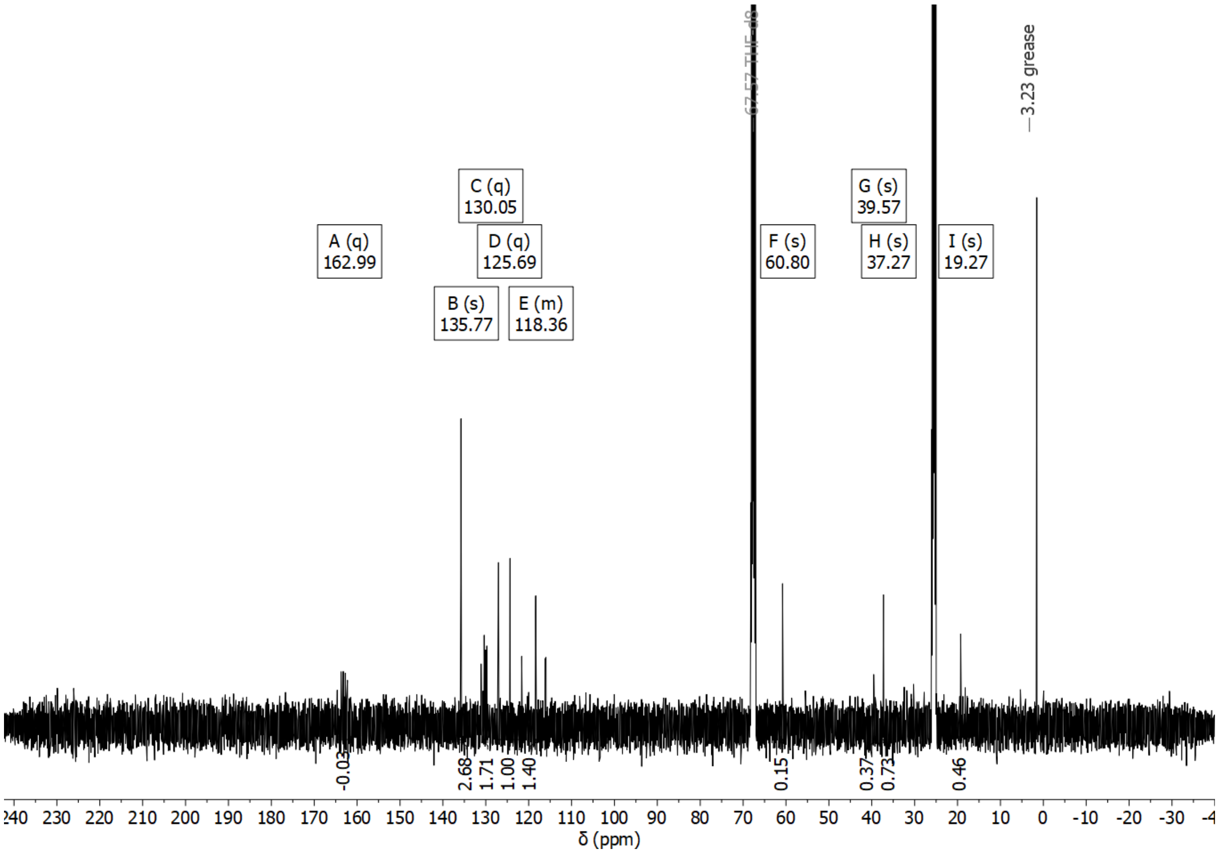

Figure S8: $^{13}C\{^{1}H\}$-NMR (THF-d_8) spectrum of $[Ti(GaTMP)_8][BAr^F{}_4]_2$ (**4**).

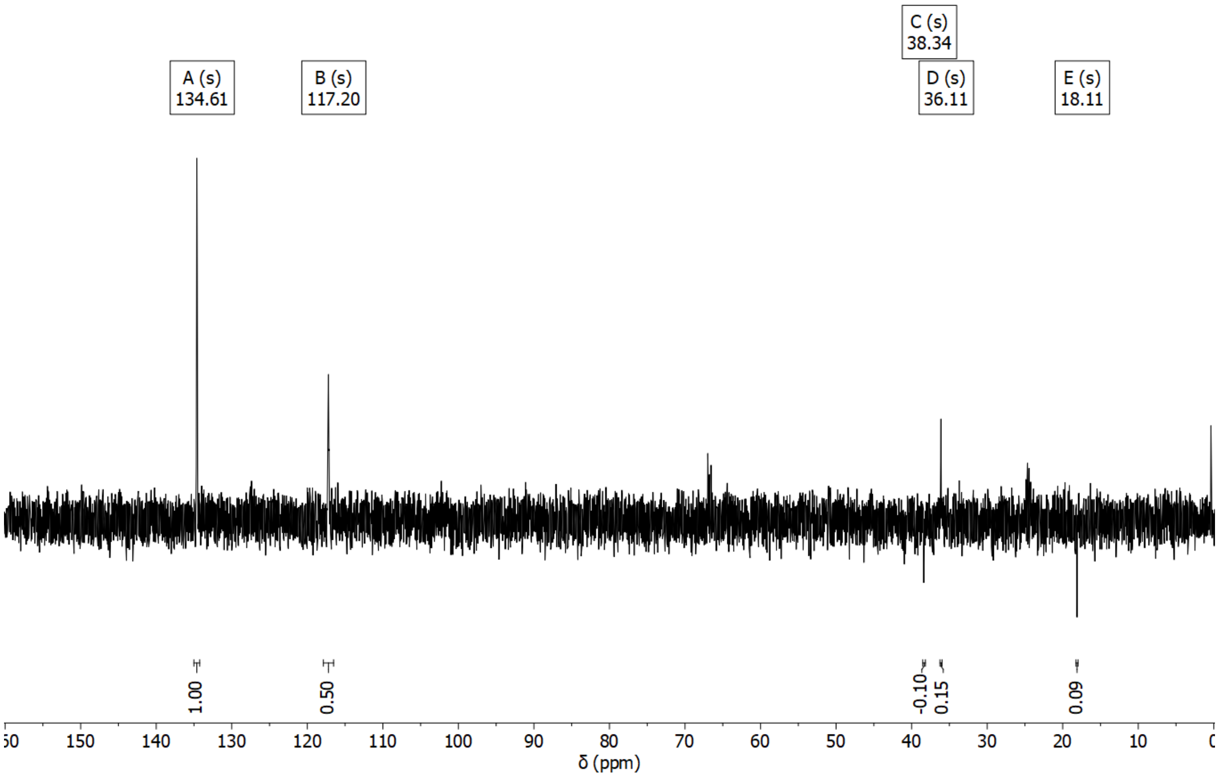

Figure S9: ^{13}C DEPT 135-NMR (THF-d_8) spectrum of $[Ti(GaTMP)_8][BAr^F{}_4]_2$ (**4**).

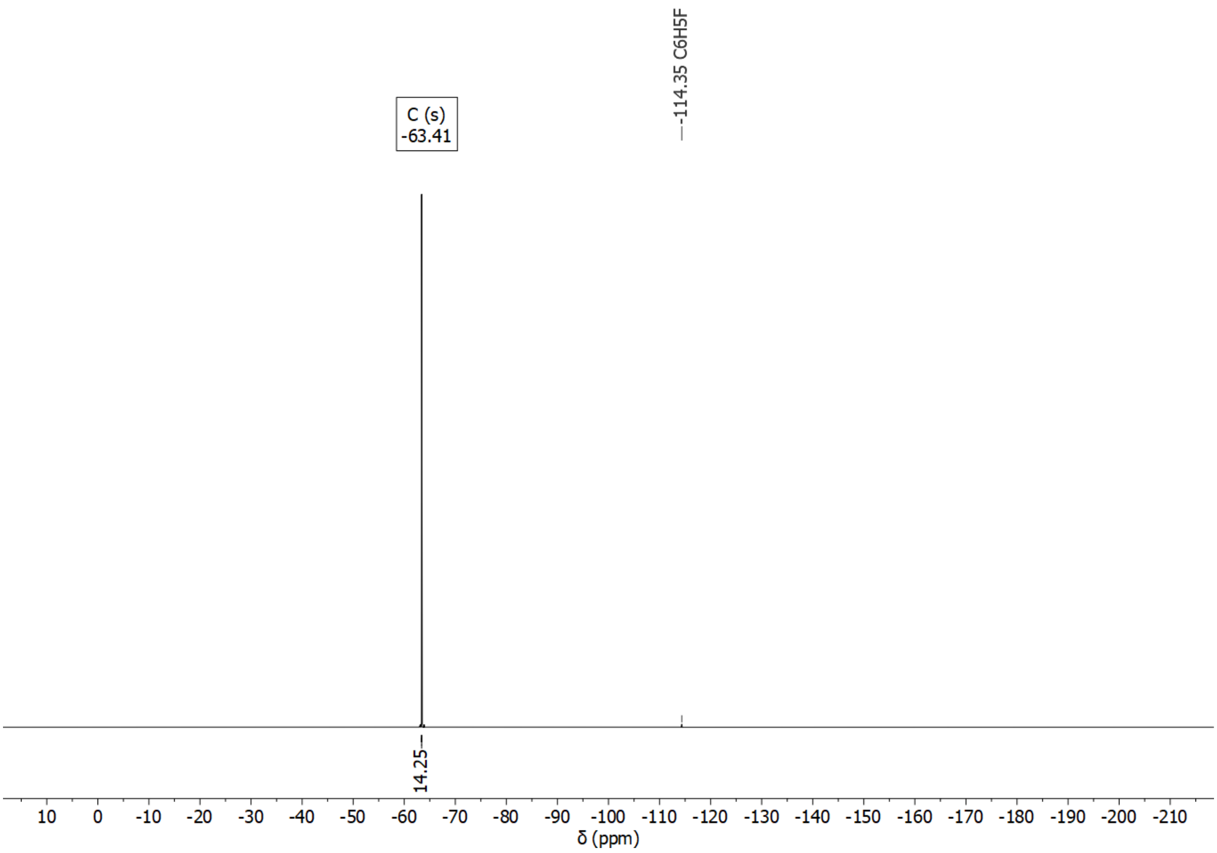

Figure S10: ^{19}F-NMR (THF-d_8) spectrum of $[Ti(GaTMP)_8][BAr^F{}_4]_2$ (**4**).

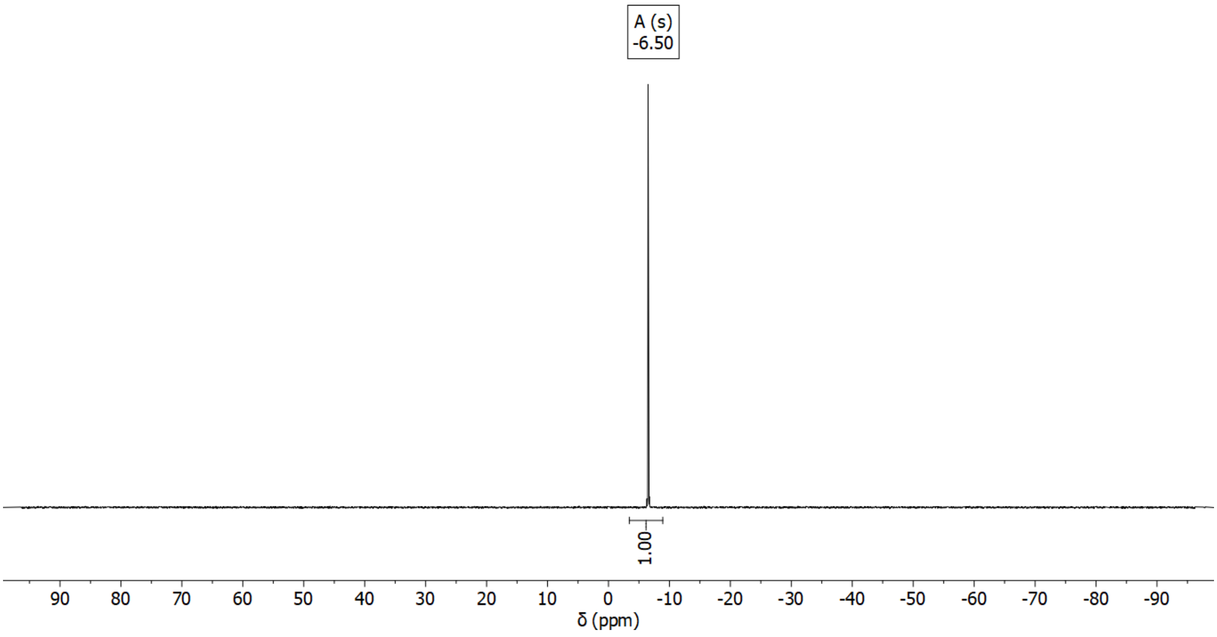

Figure S11: ^{11}B-NMR (THF-d_8) spectrum of $[Ti(GaTMP)_8][BAr^F{}_4]_2$ (**4**).

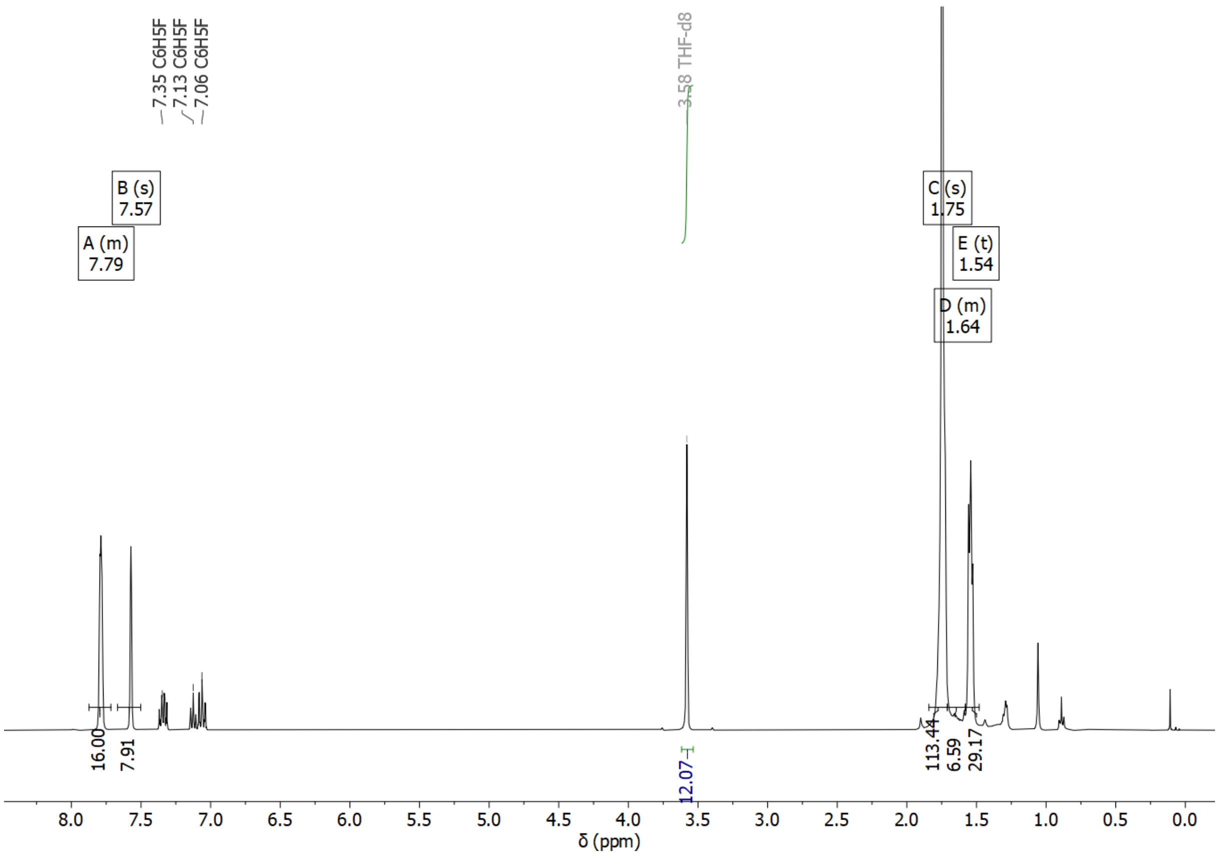

Figure S12: ^{1}H-NMR (THF-d_8) spectrum of $[Zr(GaTMP)_8][BAr^F{}_4]_2$ (**5**).

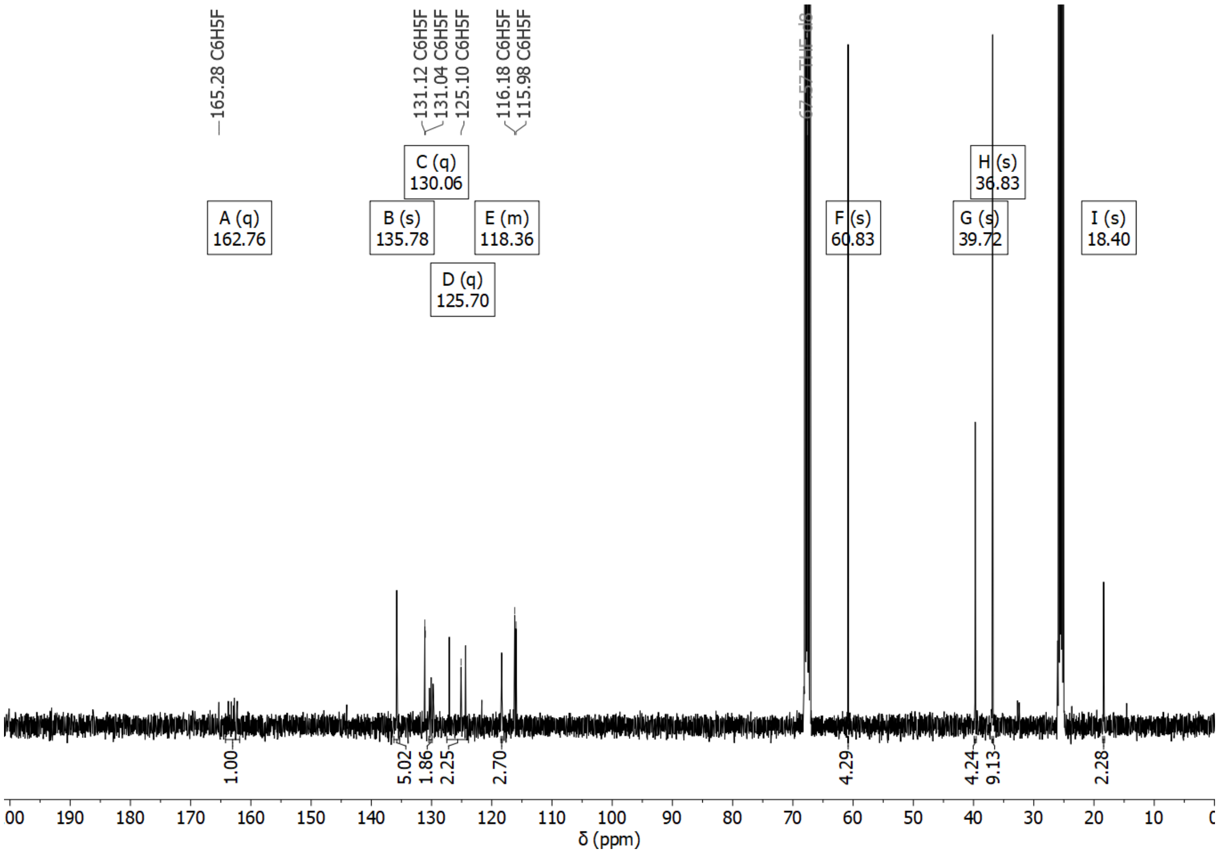

Figure S13: $^{13}C\{^{1}H\}$-NMR (THF-d_8) spectrum of $[Zr(GaTMP)_8][BAr^F{}_4]_2$ (**5**).

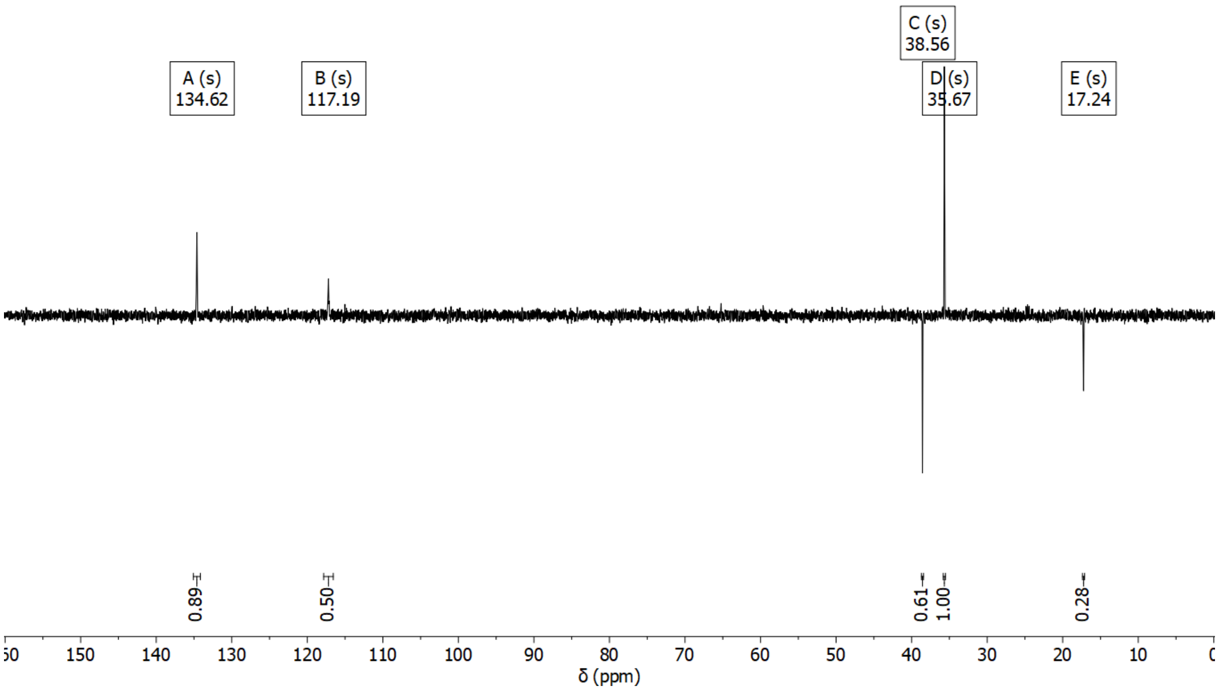

Figure S14: ^{13}C DEPT 135-NMR (THF-d_8) spectrum of $[Zr(GaTMP)_8][BAr^F{}_4]_2$ (**5**).

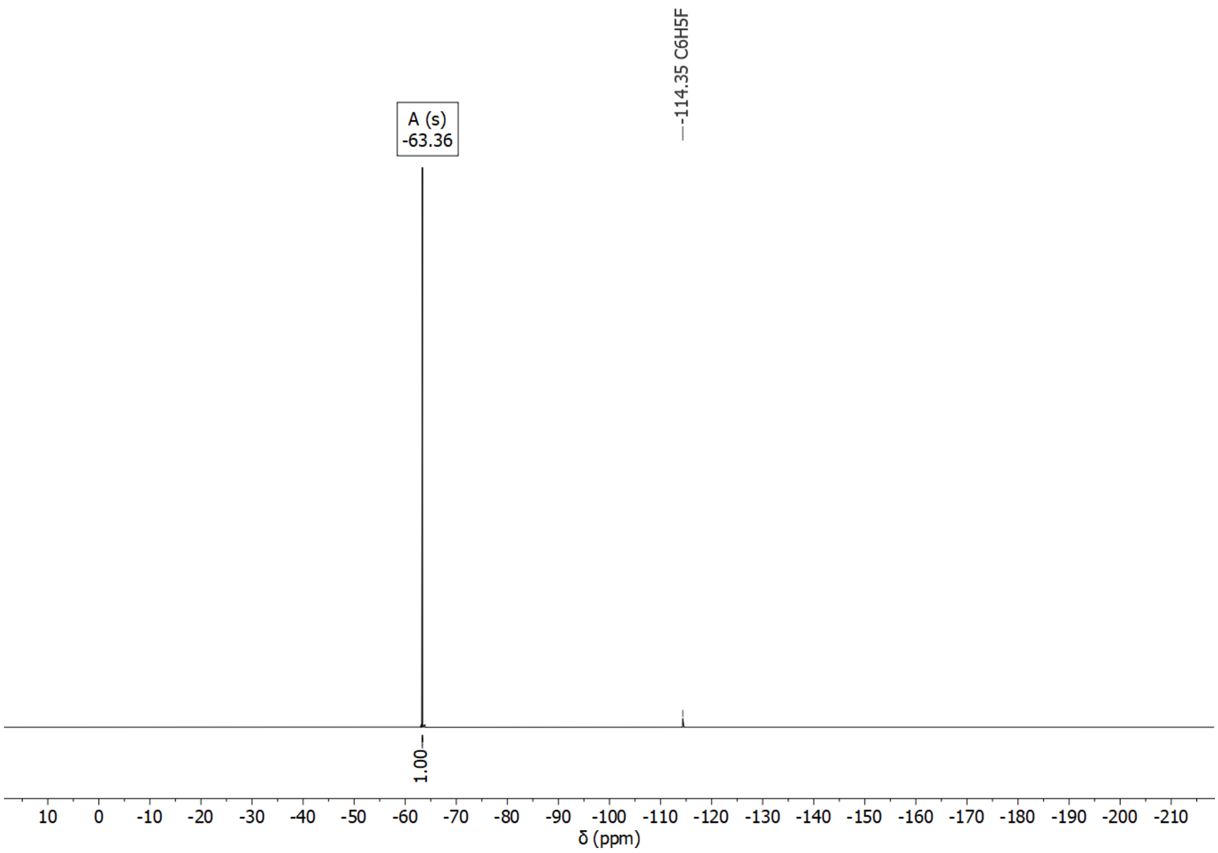

Figure S15: ^{19}F-NMR (THF-d_8) spectrum of $[Zr(GaTMP)_8][BAr^F{}_4]_2$ (**5**).

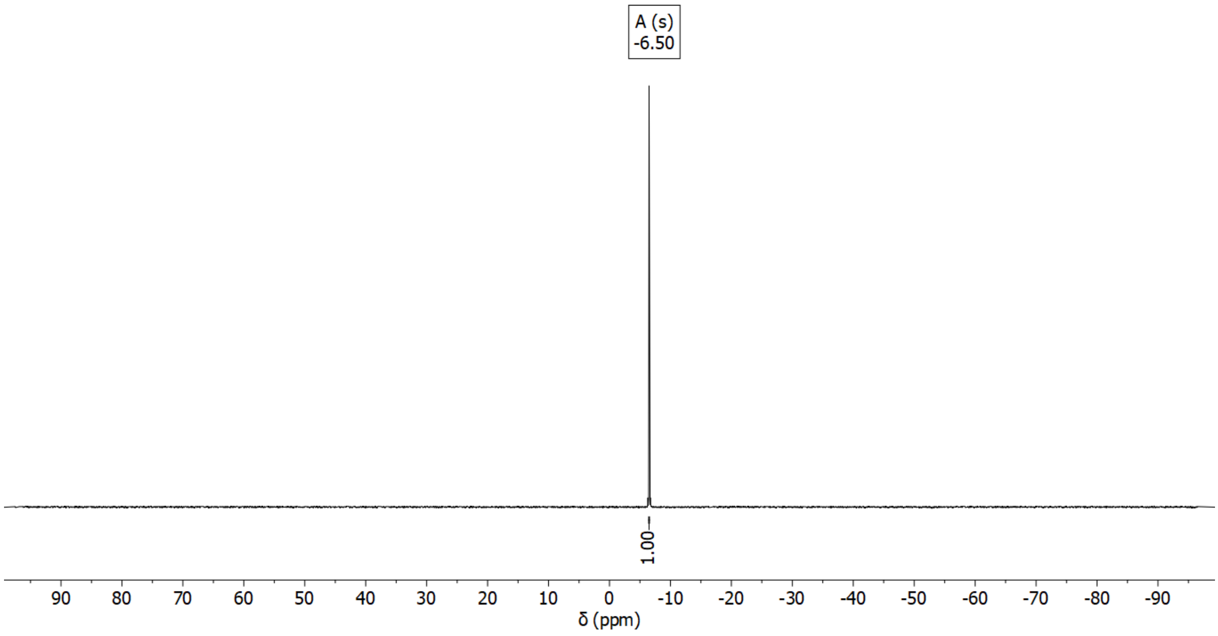

Figure S16: ^{11}B-NMR (THF-d_8) spectrum of $[Zr(GaTMP)_8][BAr^F{}_4]_2$ (**5**).

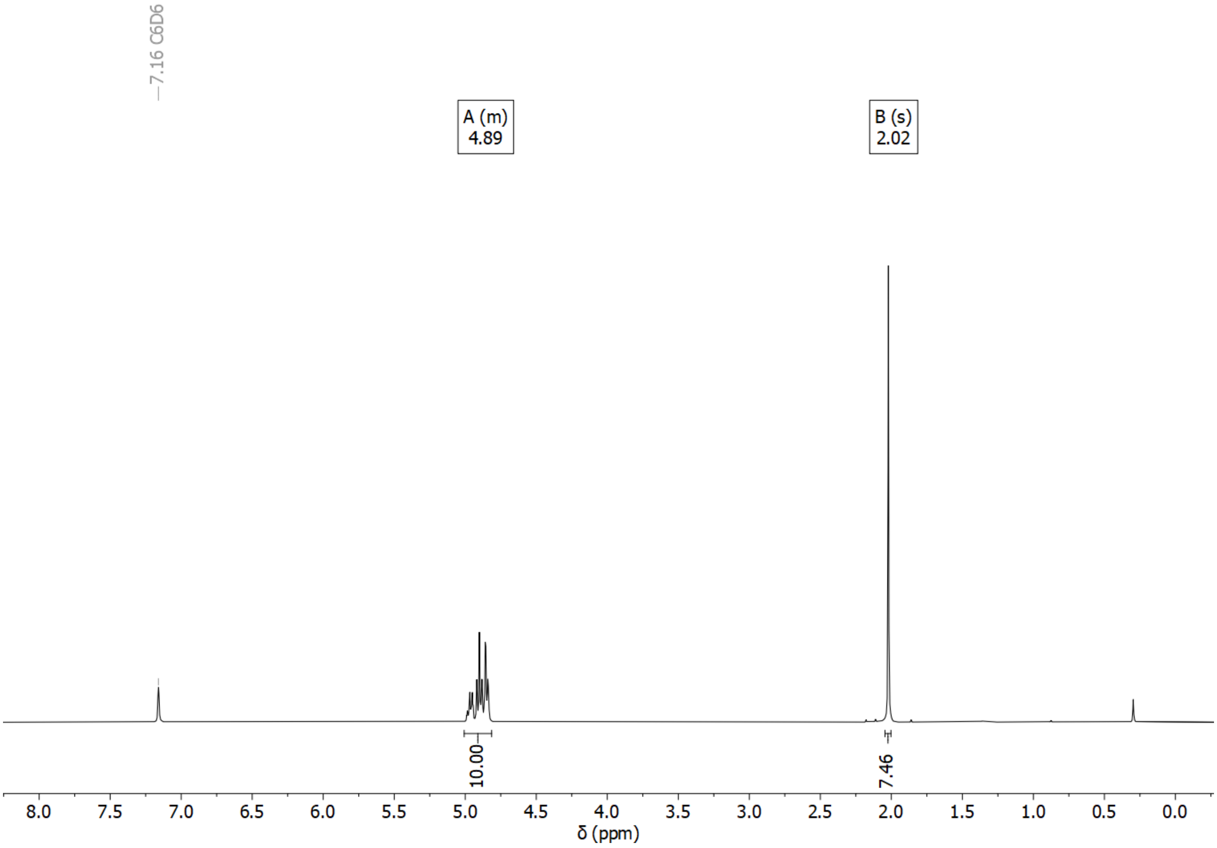

Figure S17: ^{1}H-NMR (benzene-d_6) spectrum of bis(η^6-toluene)titanium.

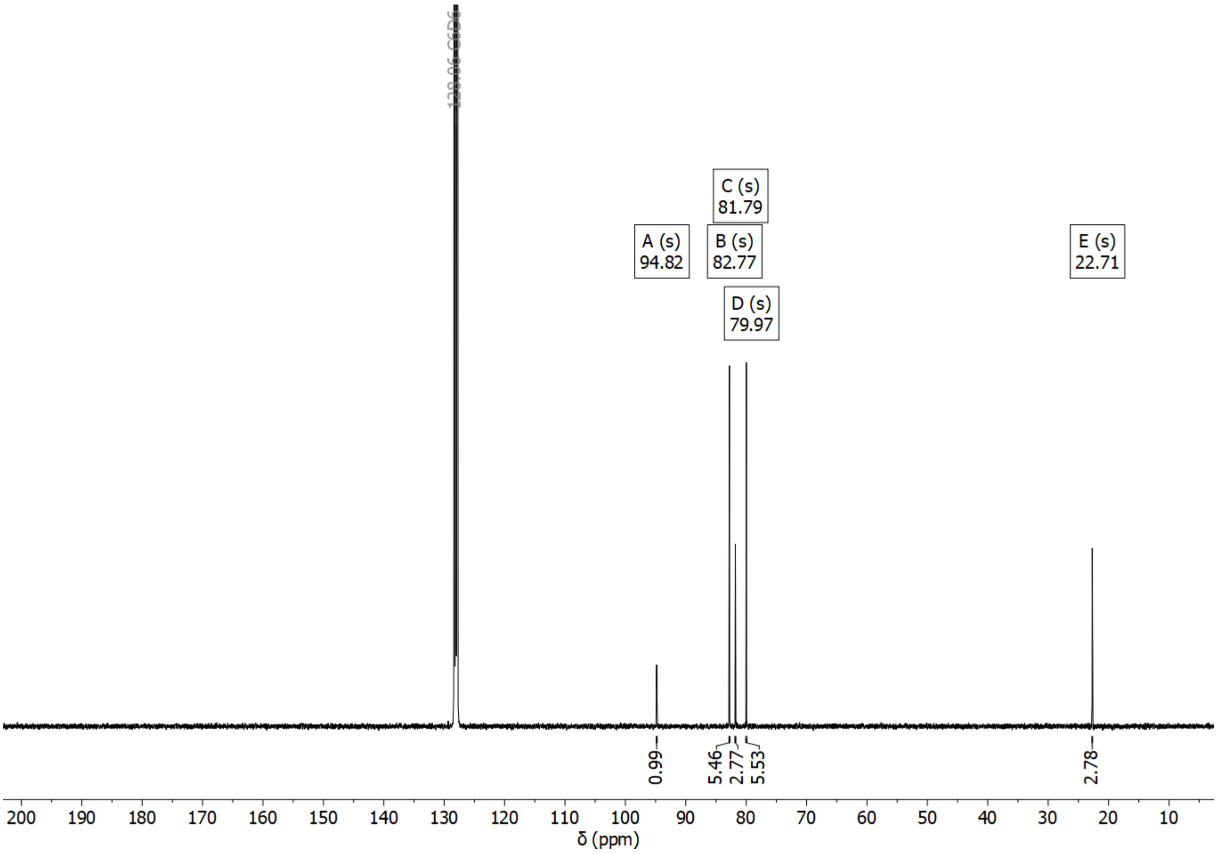

Figure S18: ^{13}C{^{1}H}-NMR (benzene-d_6) spectrum of bis(η^6-toluene)titanium.

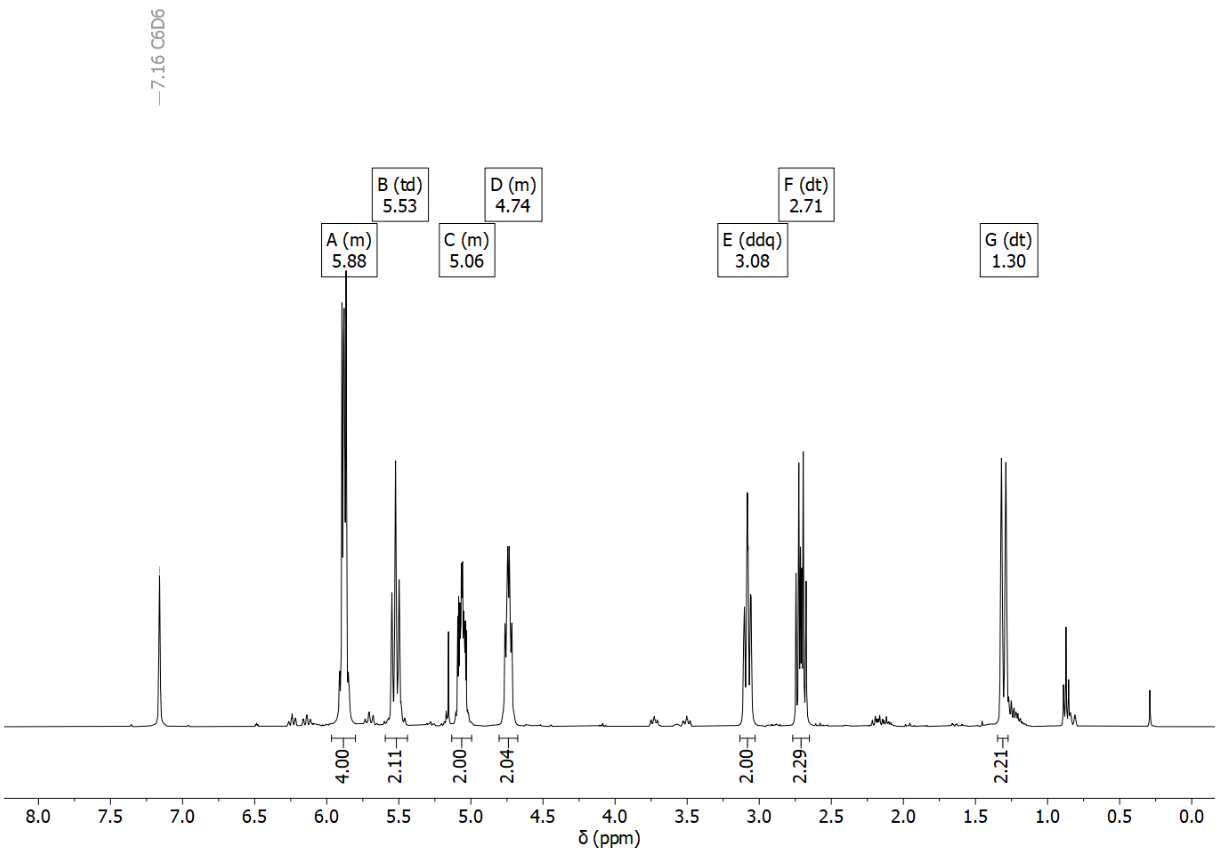

Figure S19: ^{1}H-NMR (benzene-d_6) spectrum of bis(η^6-cycloheptatriene)zirconium.

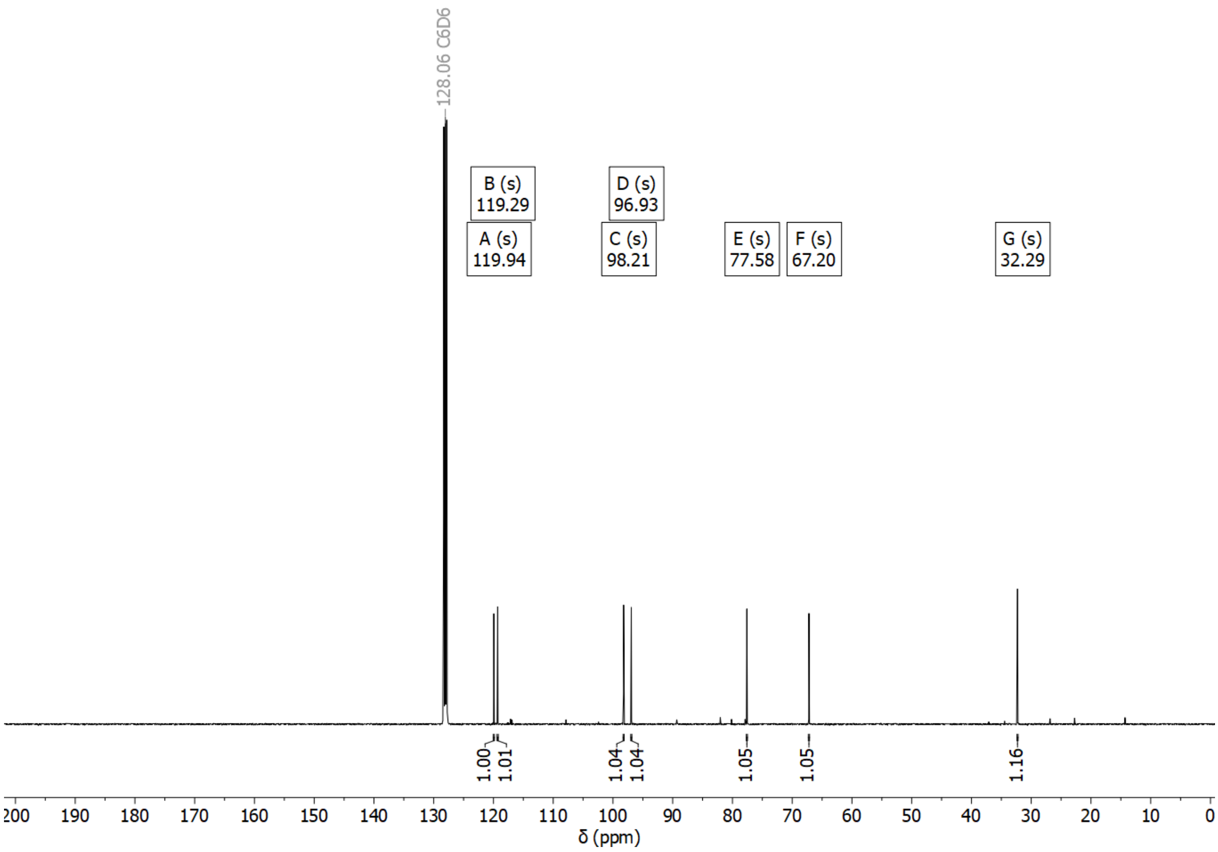

Figure S20: ^{13}C{^{1}H}-NMR (benzene-d_6) spectrum of bis(η^6-cycloheptatriene)zirconium.

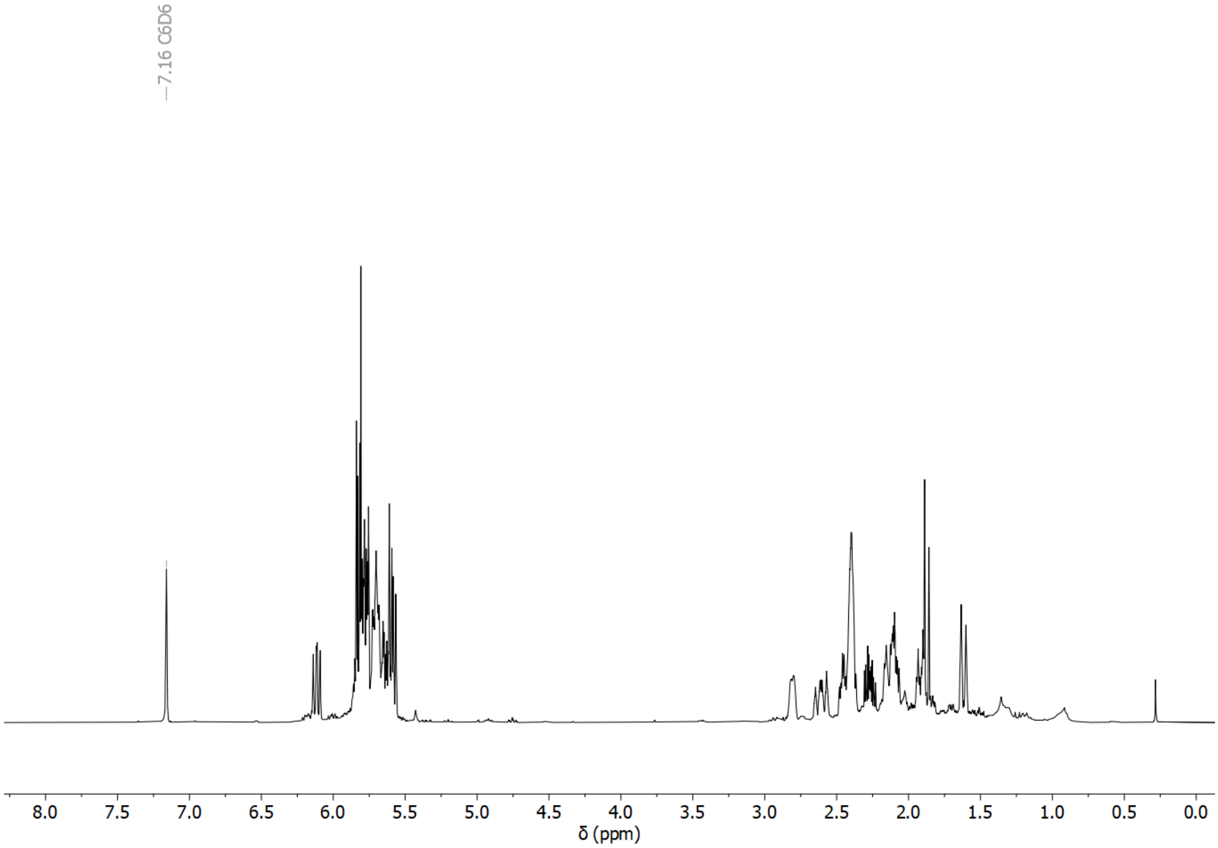

Figure S21: ^{1}H-NMR (benzene-d_6) spectrum of crude bis(η[6]-cycloheptatriene)hafnium.

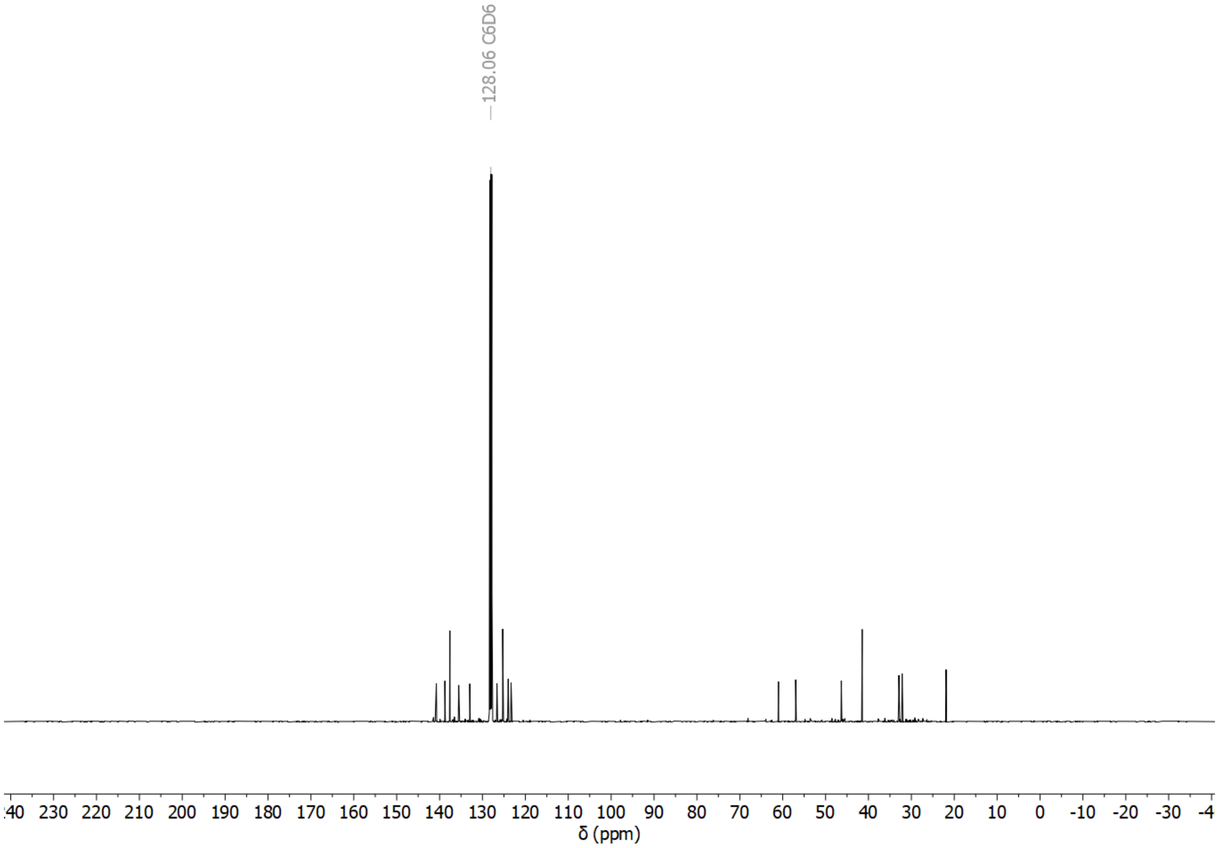

Figure S22: ^{13}C{^{1}H}-NMR (benzene-d_6) spectrum of crude bis(η[6]-cycloheptatriene)hafnium.

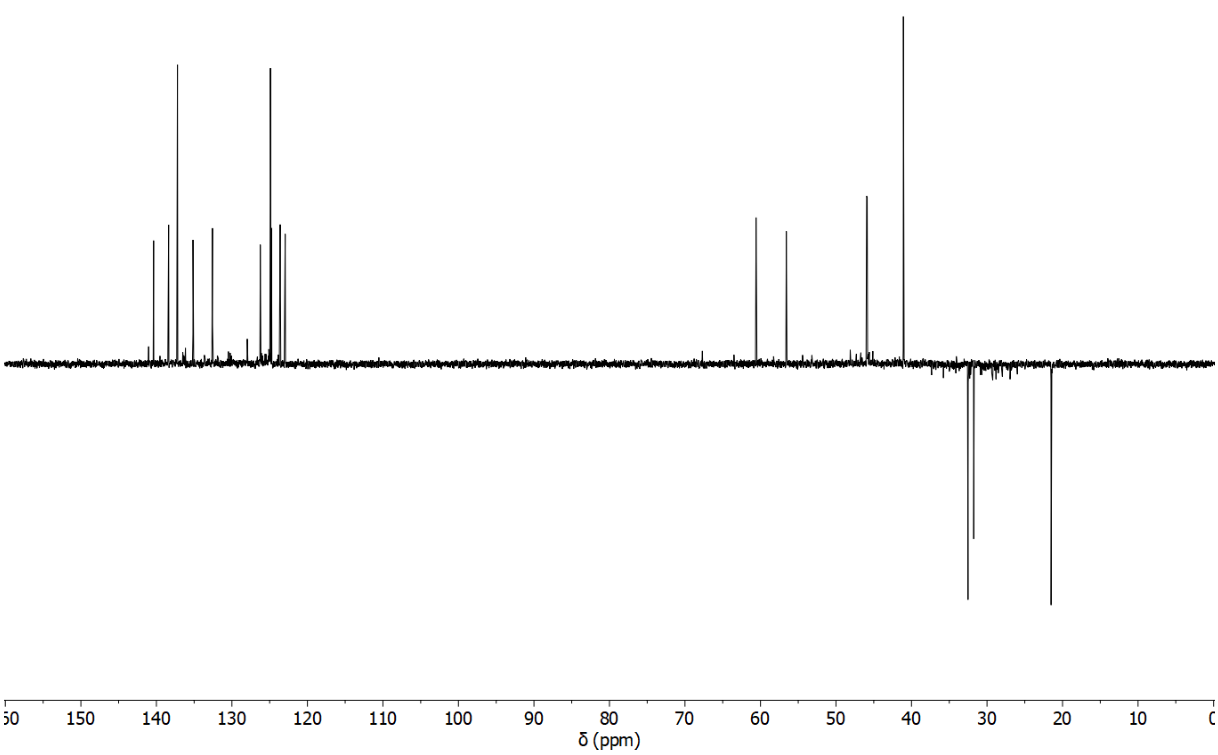

Figure S23: ^{13}C DEPT 135-NMR (benzene-d_6) spectrum of crude bis(η^6-cycloheptatriene)hafnium.

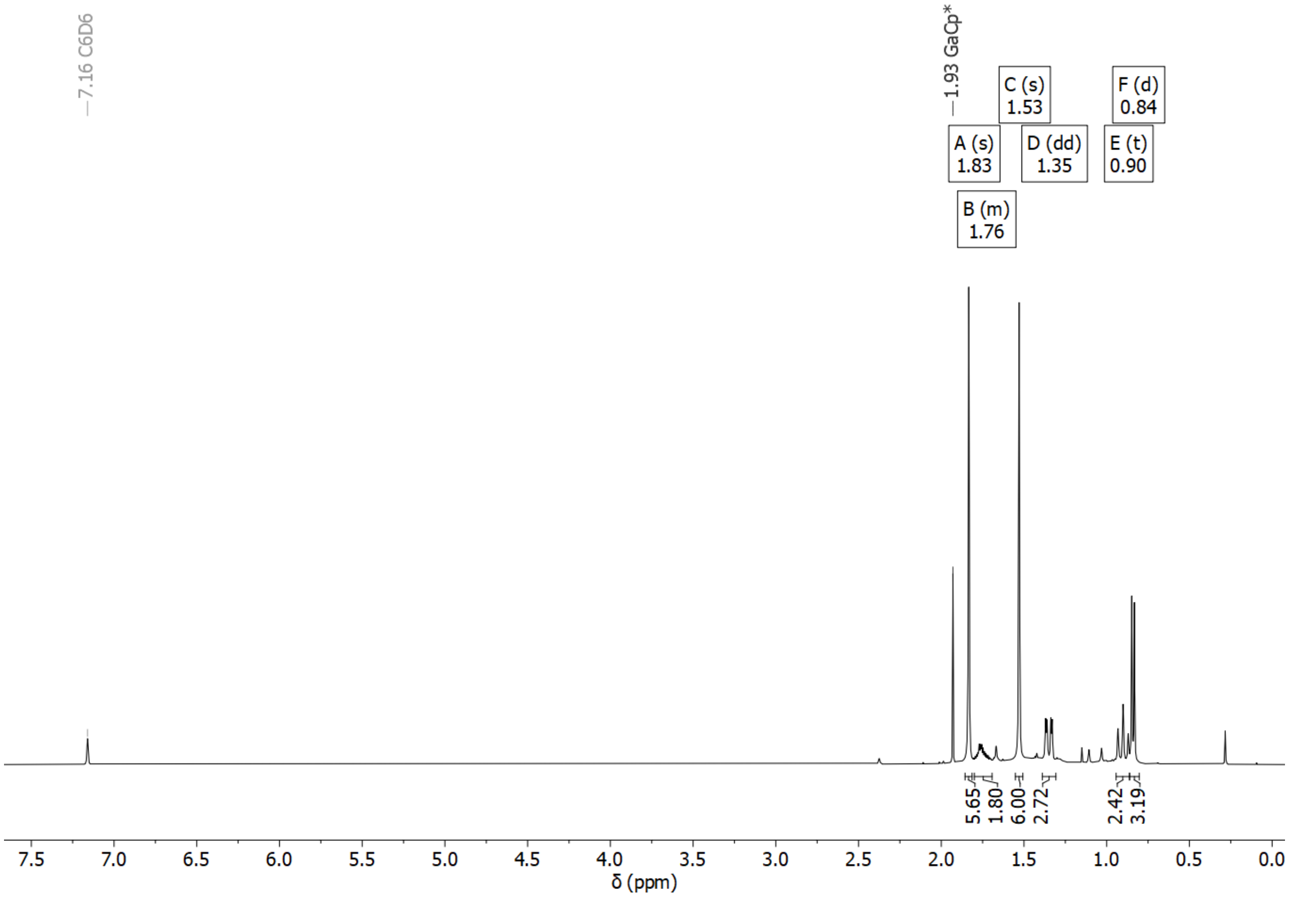

Figure S24: ^{1}H-NMR (benzene-d_6) spectrum of GaPMP.

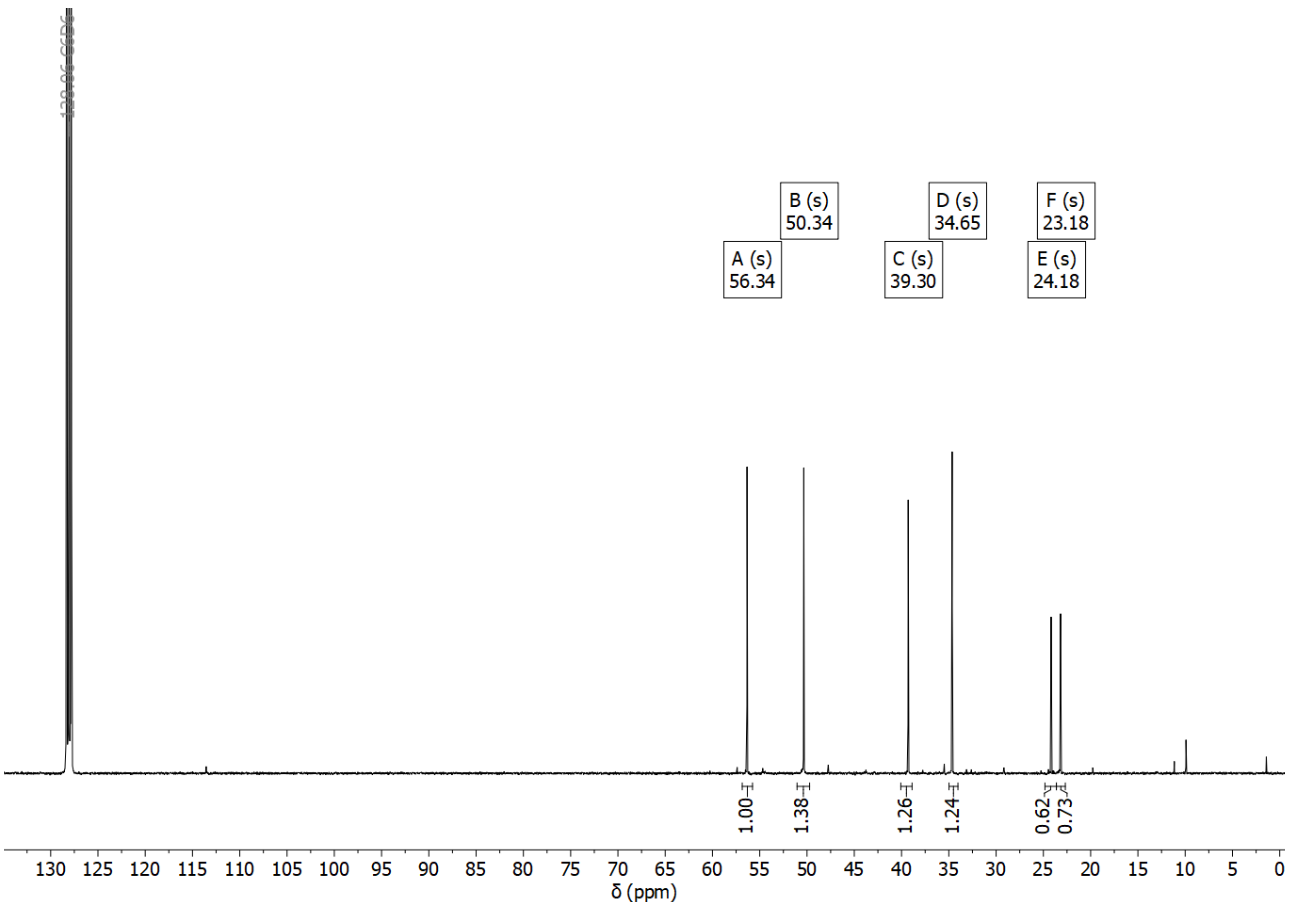

Figure S25: $^{13}C\{^{1}H\}$-NMR (benzene-d_6) spectrum of GaPMP.

5. LIFDI-MS

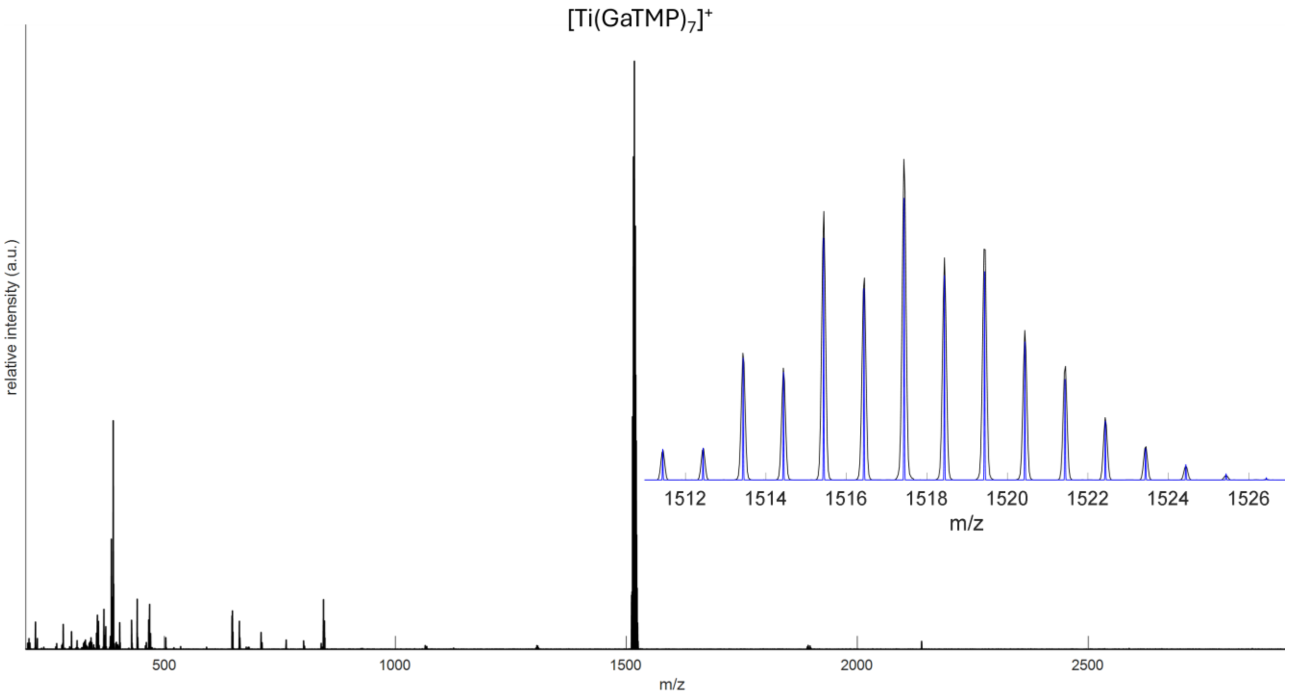

Figure S26: HR LIFDI-MS of [Ti(GaTMP)$_7$] (**1**) and comparison of calculated and experimental patterns (inset); [Ti(GaTMP)$_7$]$^+$ (exp.) 1517.4341 m/z (calc.) 1517.4332 m/z.

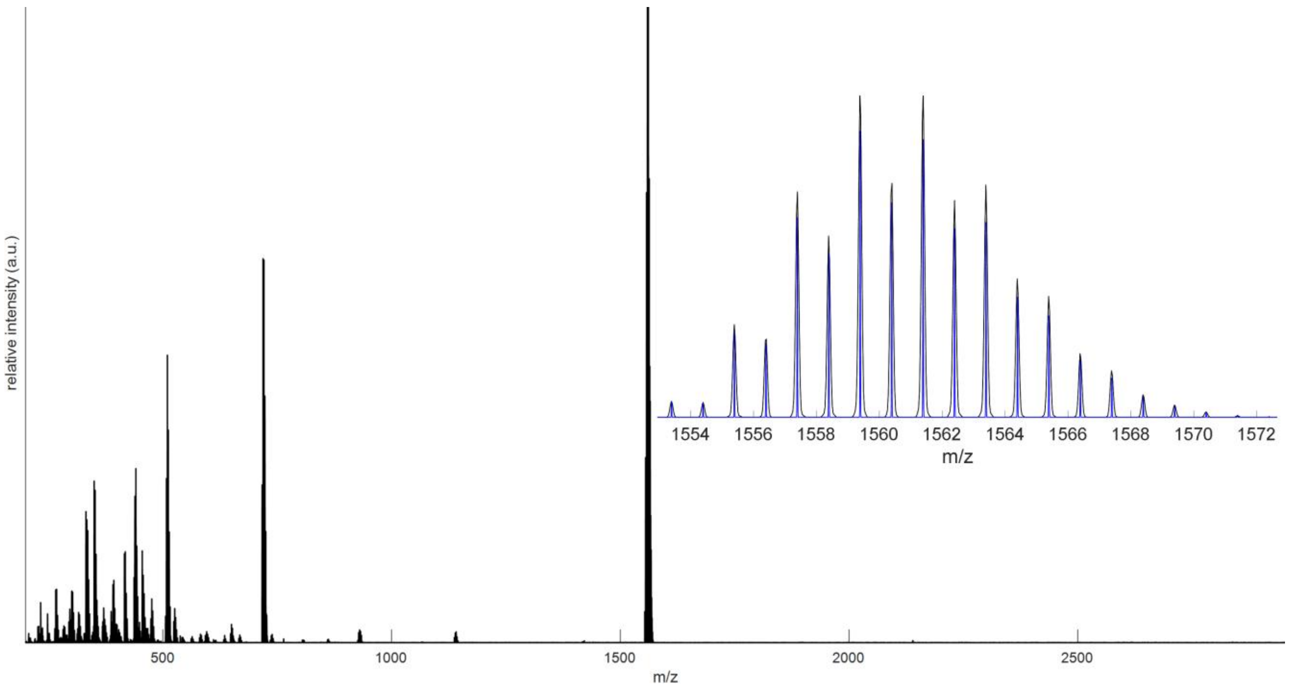

Figure S27: HR LIFDI-MS of [Zr(GaTMP)$_7$] (**2**) and comparison of calculated and experimental patterns; (inset); [Zr(GaTMP)$_7$]$^+$ exp.: 1559.3866 m/z; calc.: 1559.3901 m/z.

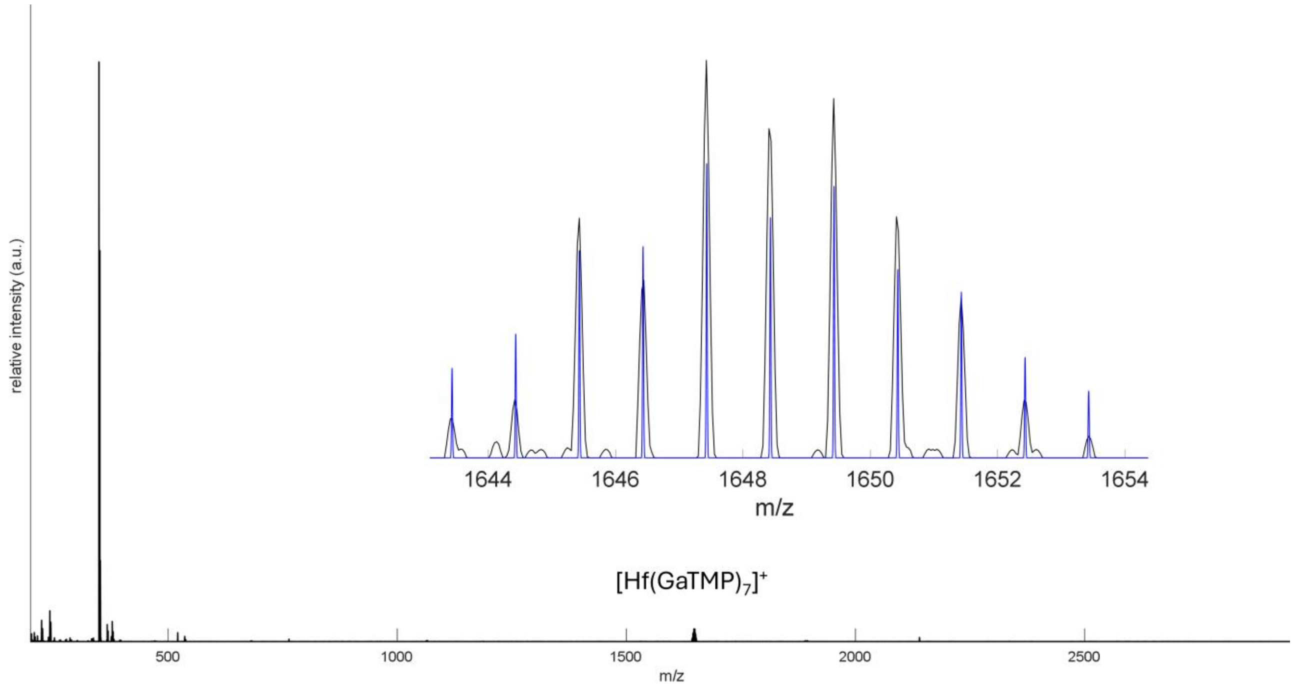

Figure S28: HR LIFDI-MS of [Hf(GaTMP)$_7$] (**3**) and comparison of calculated and experimental patterns; (inset); [Hf(GaTMP)$_7$]$^+$ exp.: 1647.4265 m/z; calc.: 1647.4311 m/z.

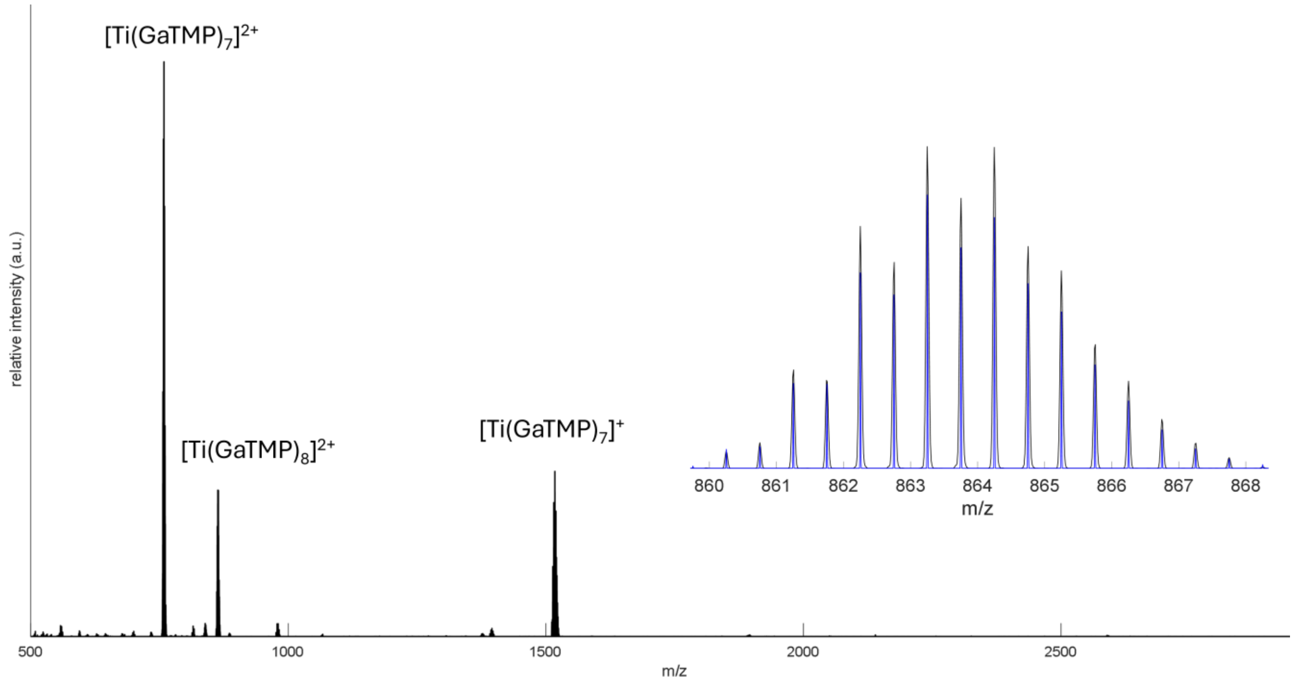

Figure S29: HR LIFDI-MS of [Ti(GaTMP)$_8$](BAr^F)$_2$ (**4**) and comparison of calculated and experimental patterns; (inset); [Ti(GaTMP)$_8$]$^{2+}$ exp.: 863.2499 m/z; calc.: 863.2501 m/z.

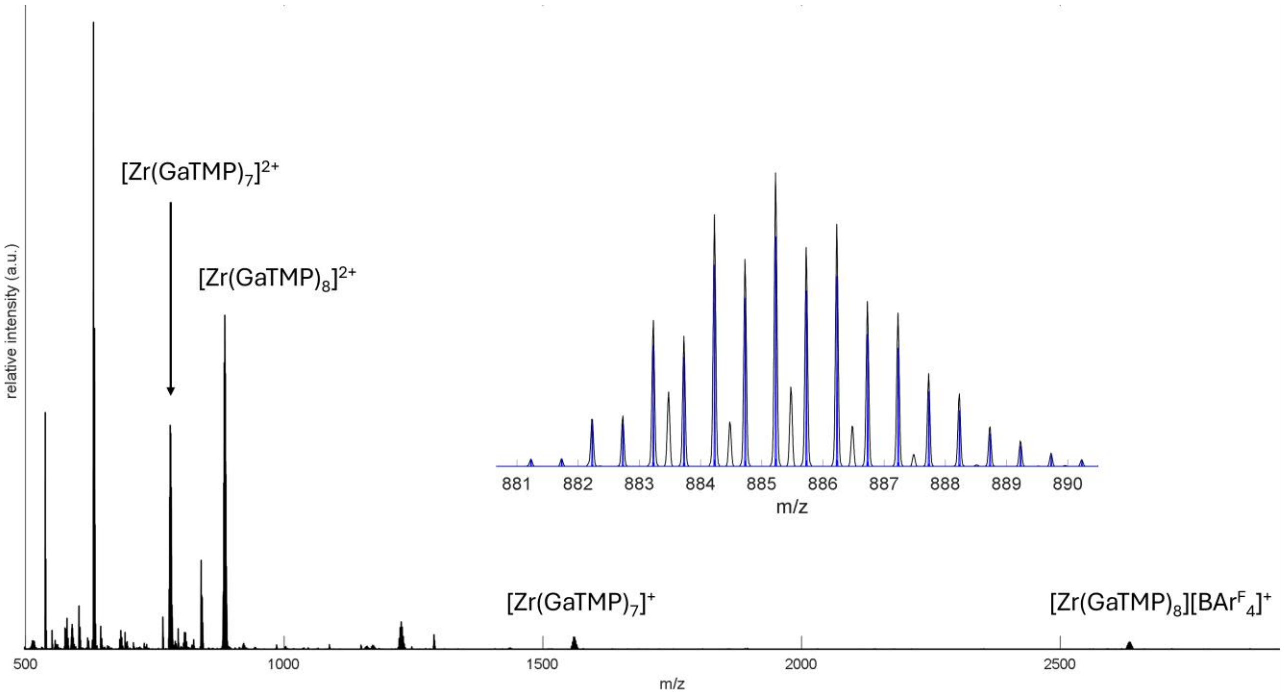

Figure S30: HR LIFDI-MS of $[Zr(GaTMP)_8](BAr^F)_2$ (**5**) and comparison of calculated and experimental patterns; (inset); $[Zr(GaTMP)_8]^{2+}$ exp.: 885.2262 m/z; calc.: 885.2281 m/z.

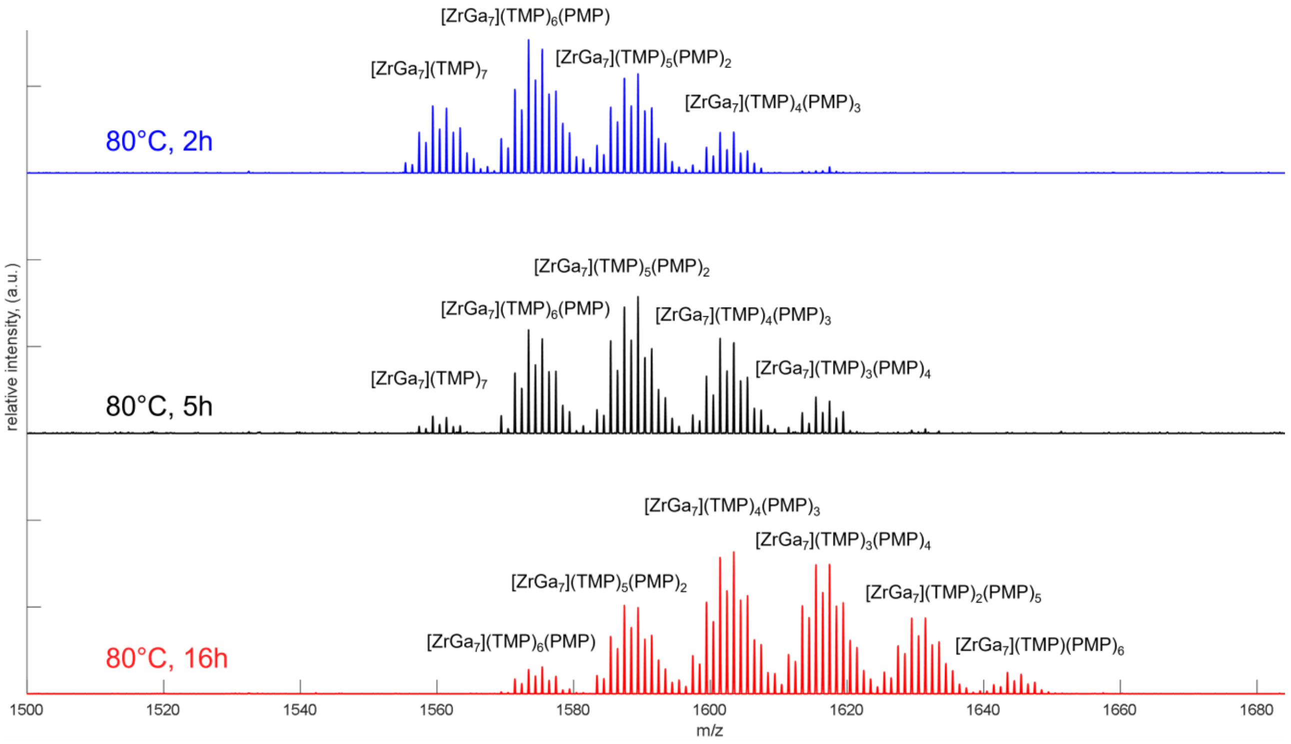

Figure S31: *In situ* HR LIFDI-MS of the ligand exchange between **2** and GaPMP in toluene at 80°C.

6. IR Spectra

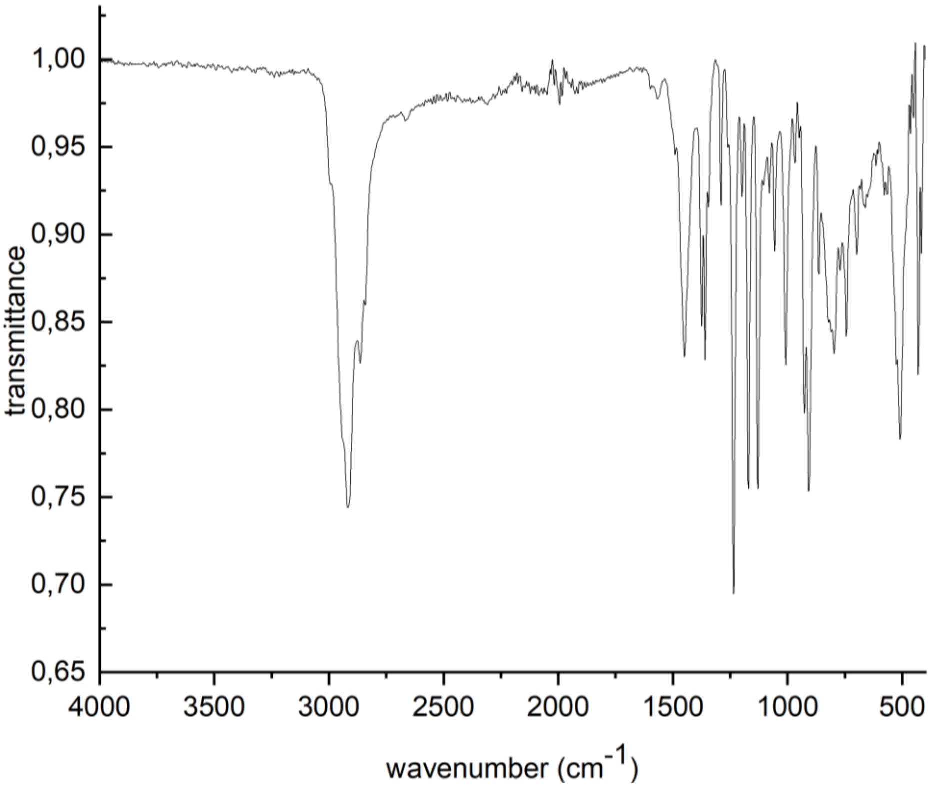

Figure S32: ATR-IR spectrum of [Ti(GaTMP)$_7$] (**1**).

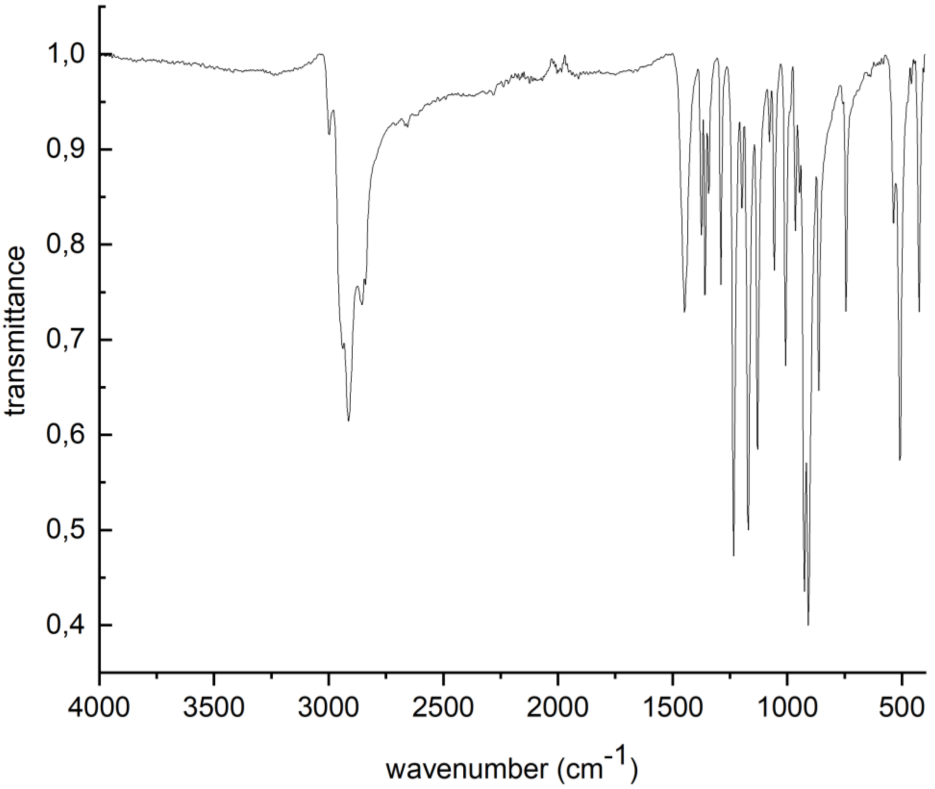

Figure S33: ATR-IR spectrum of [Zr(GaTMP)$_7$] (**2**).

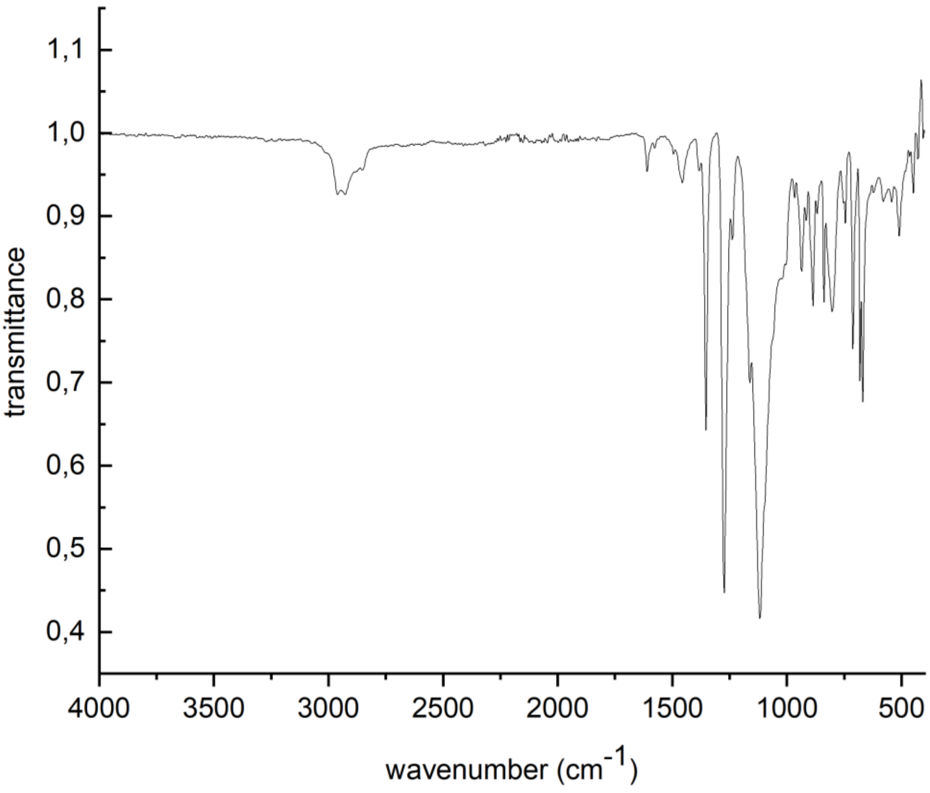

Figure S34: ATR-IR spectrum of $[Ti(GaTMP)_8](BAr^F)_2$ (**4**).

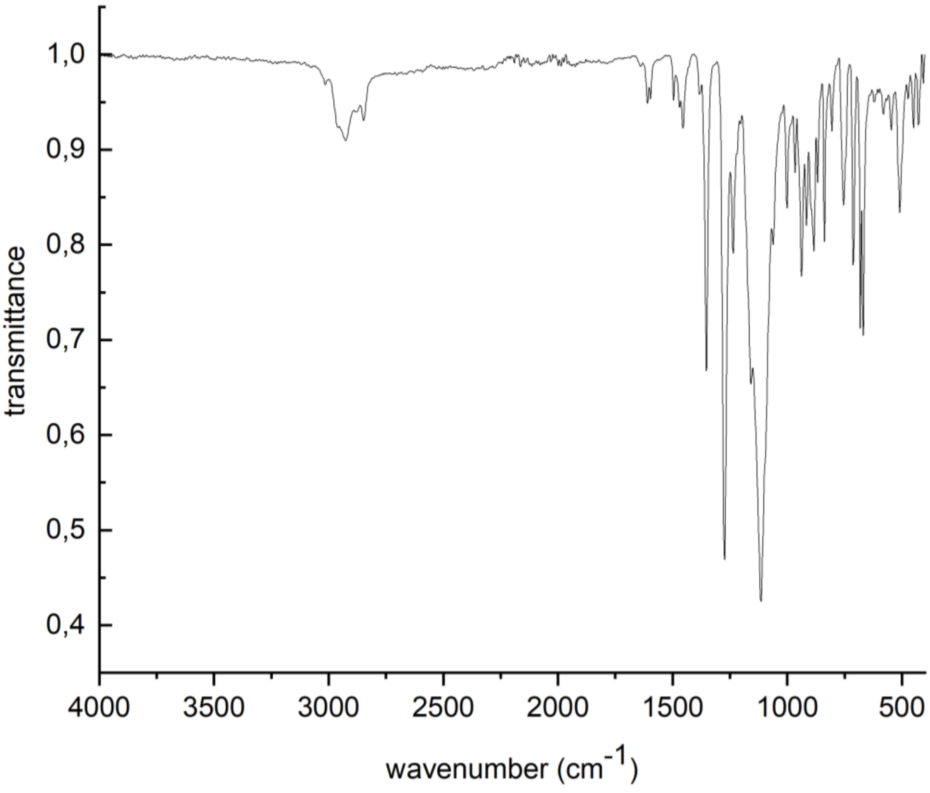

Figure S35: ATR-IR spectrum of $[Zr(GaTMP)_8](BAr^F)_2$ (**5**).

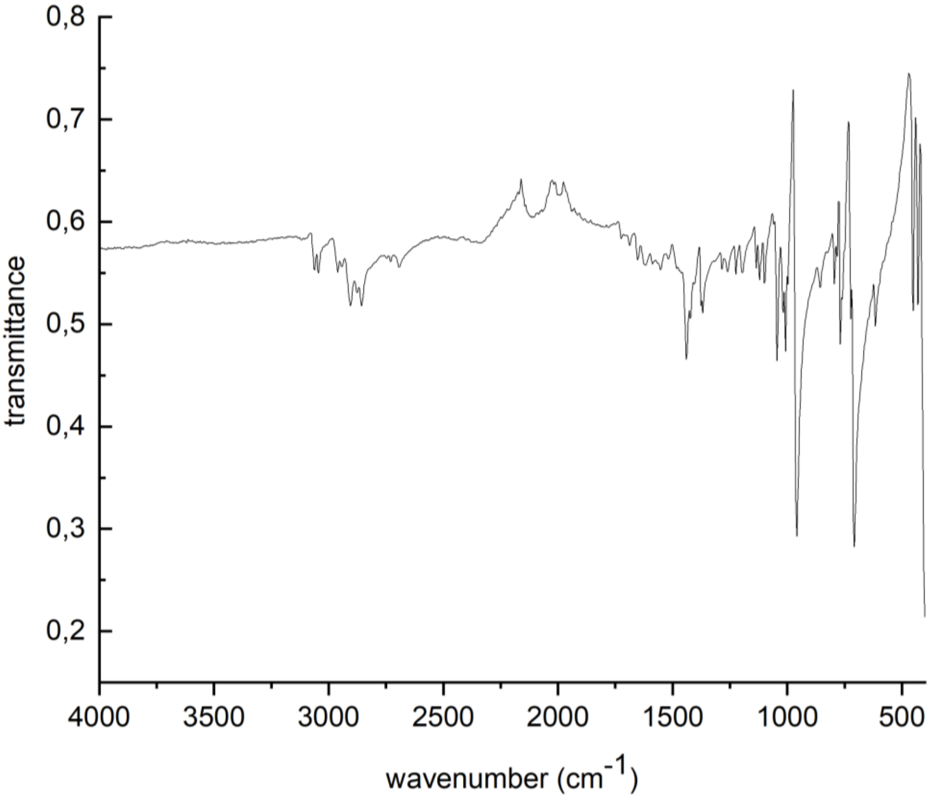

Figure S36: ATR-IR spectrum of bis(η^6-toluene)titanium.

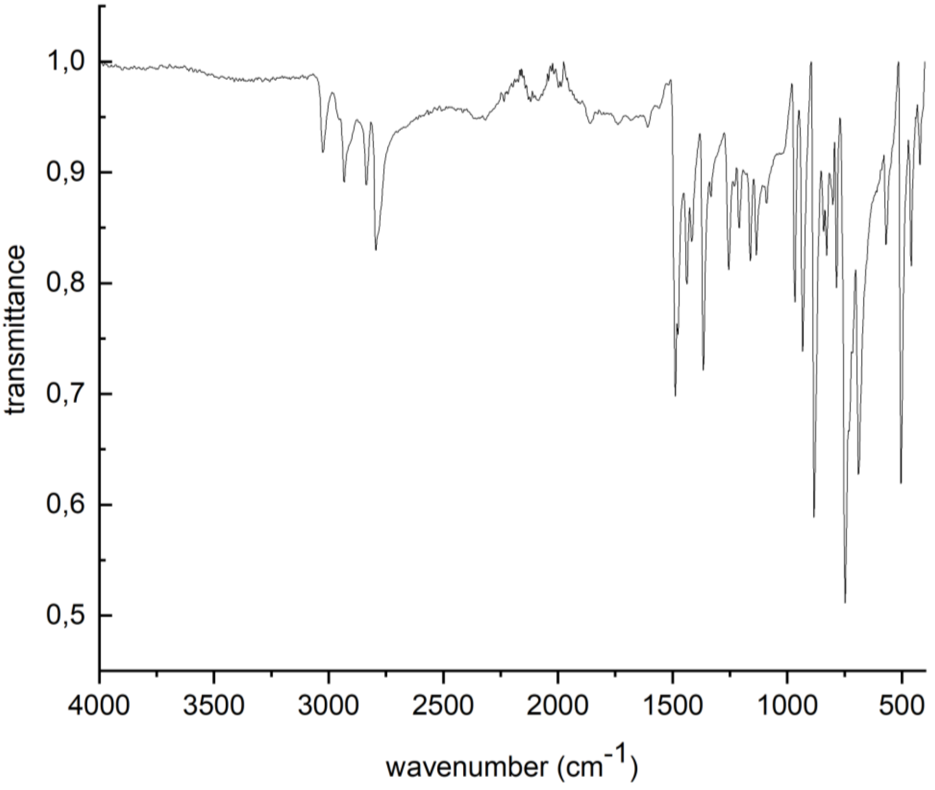

Figure S37: ATR-IR spectrum of bis(η^6-cycloheptatriene)zirconium.

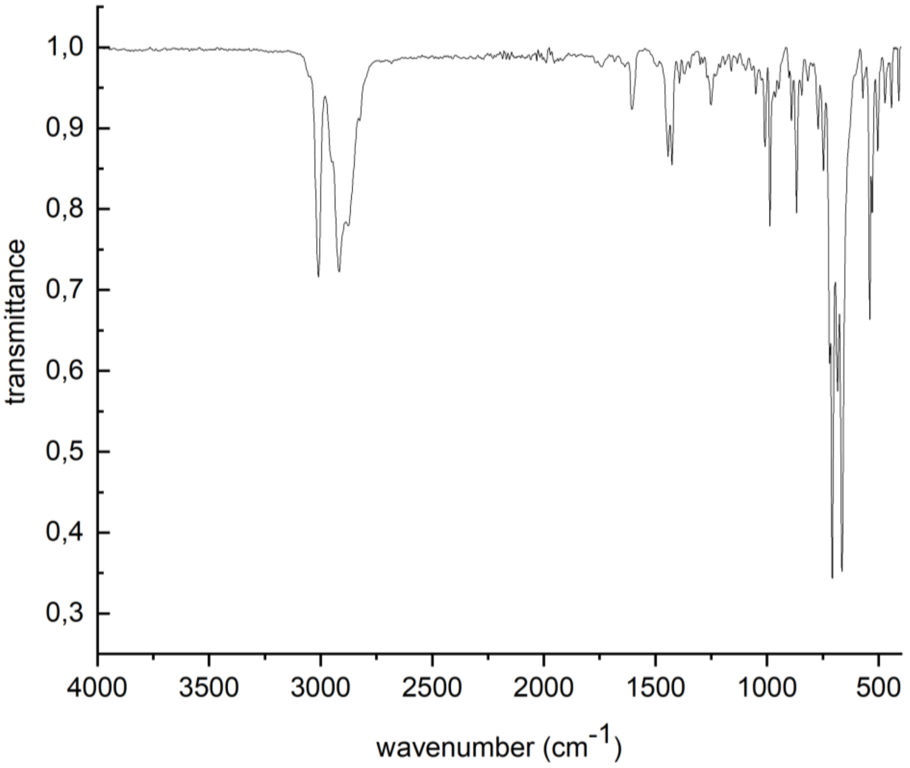

Figure S38: ATR-IR spectrum of crude bis(η[6]-cycloheptatriene)hafnium.

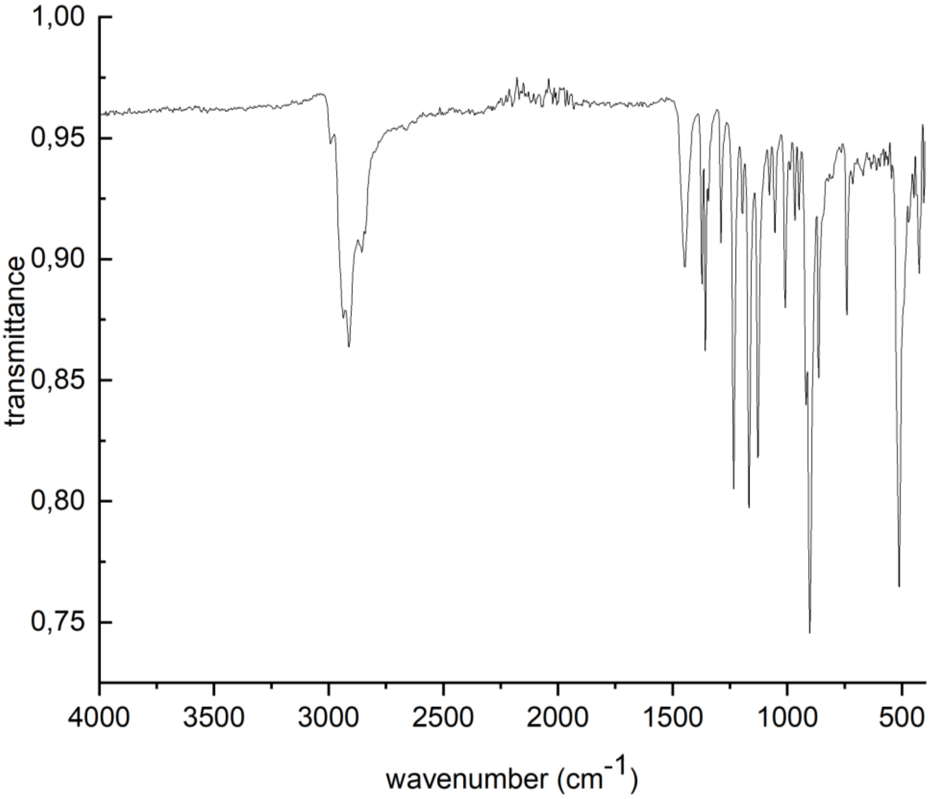

Figure S39: ATR-IR spectrum of GaTMP.

7. RAMAN Spectra

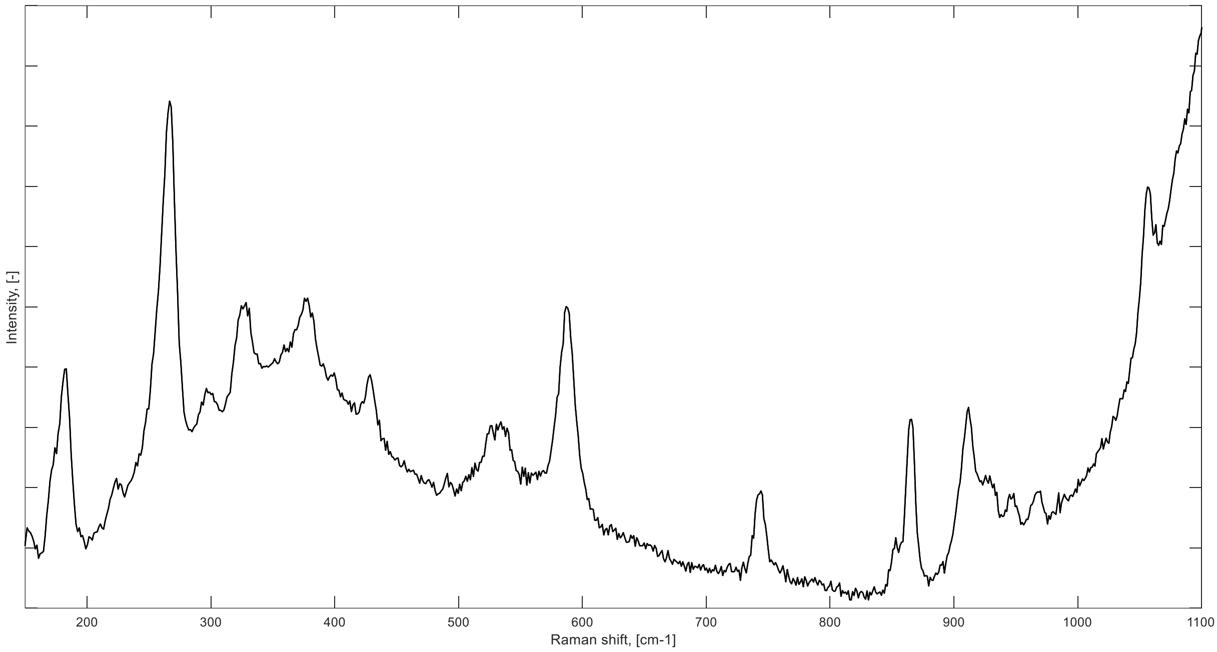

Figure S40: Raman spectrum of [Ti(GaTMP)$_7$] (**1**) obtained with 785 nm laser.

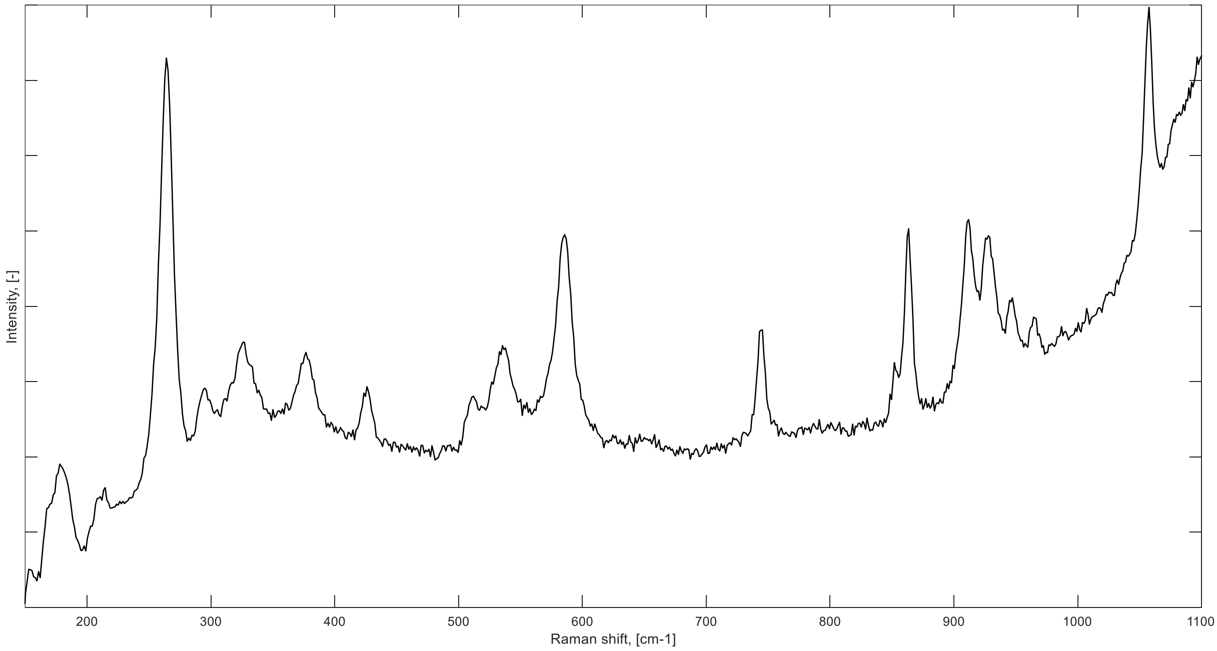

Figure S41: Raman spectrum of [Zr(GaTMP)$_7$] (**2**) obtained with 785 nm laser.

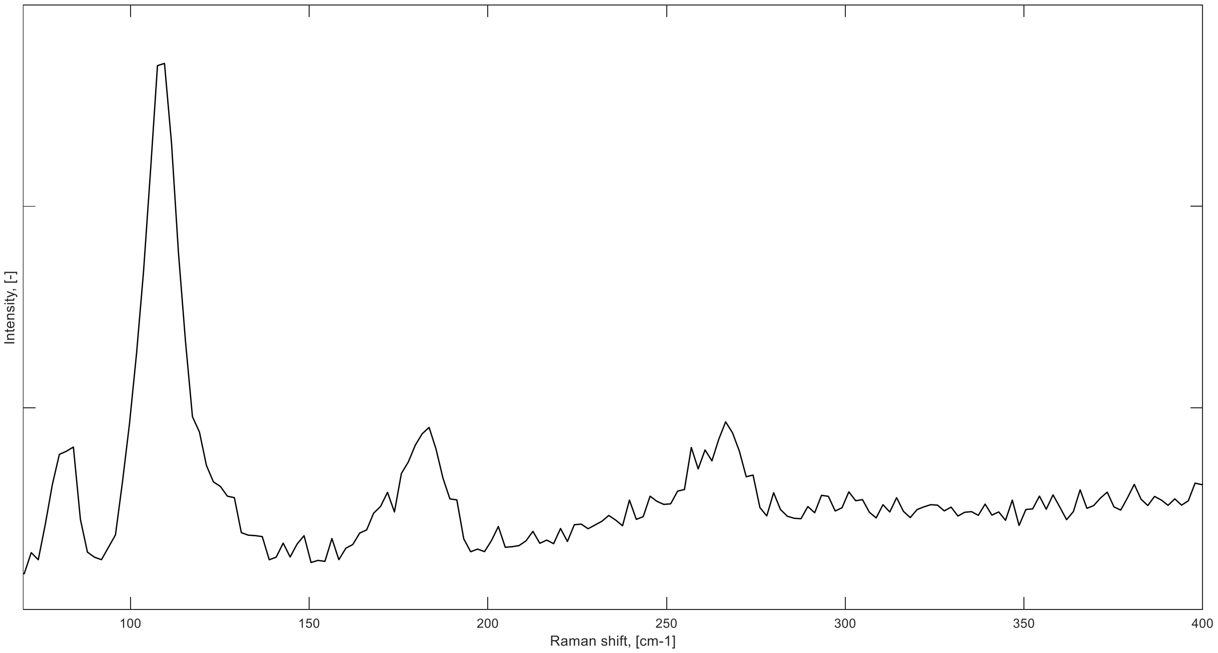

Figure S42: Raman spectrum of [Ti(GaTMP)$_7$] (**1**) obtained with 532 nm laser.

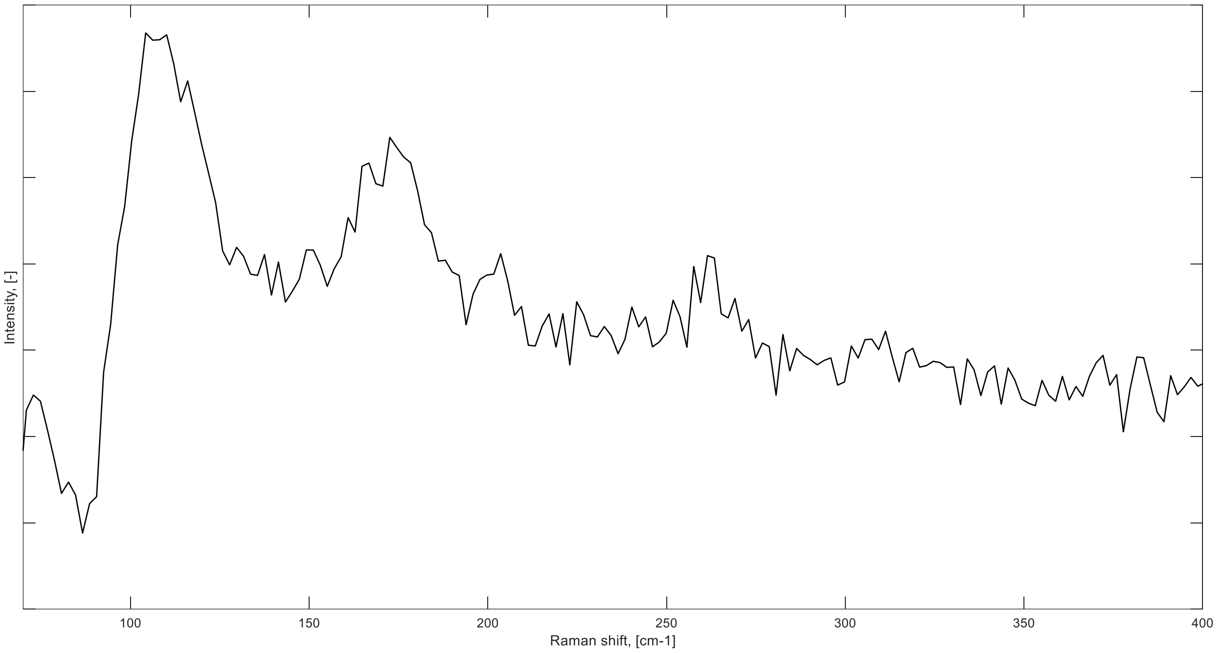

Figure S43: Raman spectrum of [Zr(GaTMP)$_7$] (**2**) obtained with 532 nm laser.

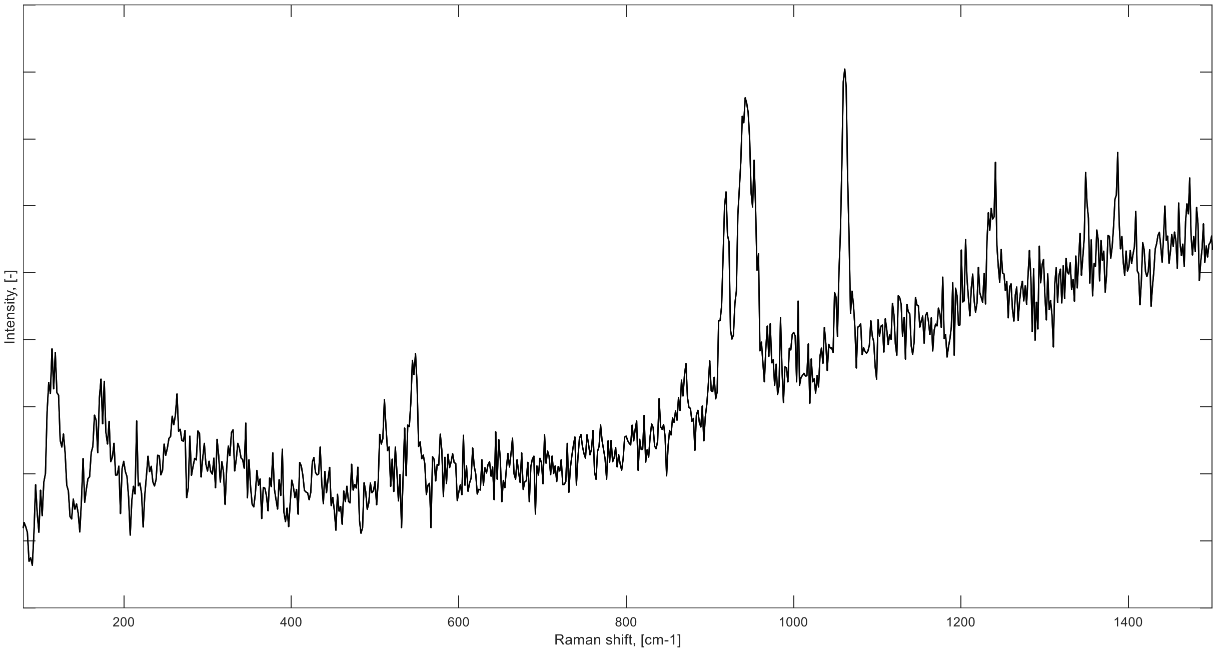

Figure S44: Raman spectrum of $[Zr(GaTMP)_8](BAr^F)_2$ (**5**) obtained with 532 nm laser.

8. UV-Vis Spectra

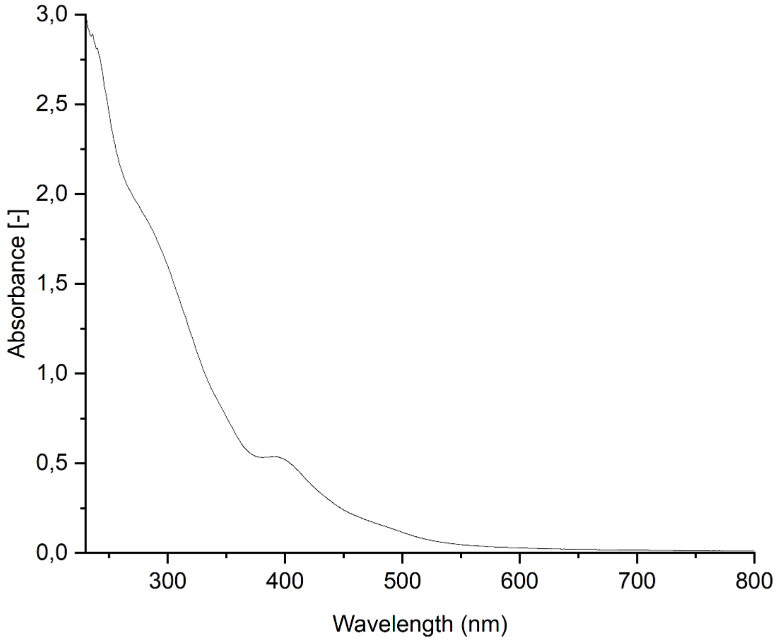

Figure S45: UV-Vis spectrum of [Ti(GaTMP)$_7$] (**1**) in *n*-hexane.

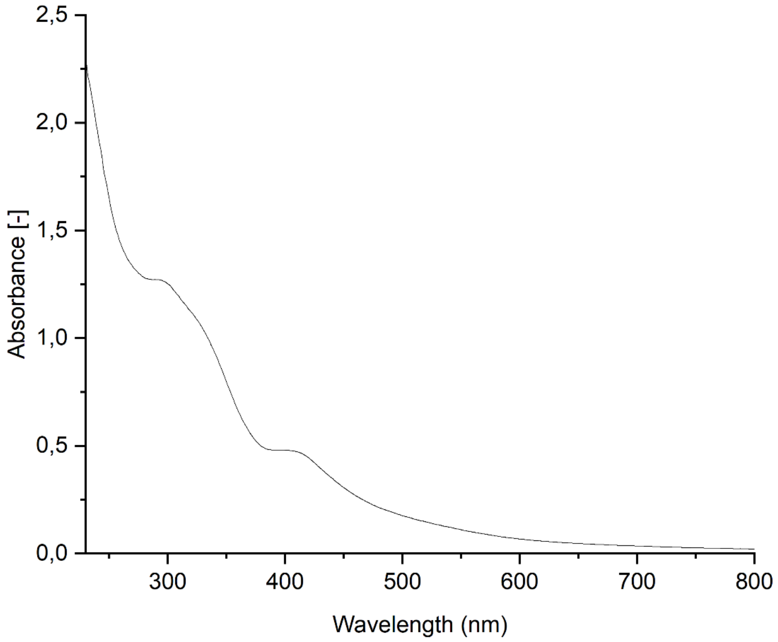

Figure S46: UV-Vis spectrum of [Zr(GaTMP)$_7$] (**2**) in *n*-hexane.

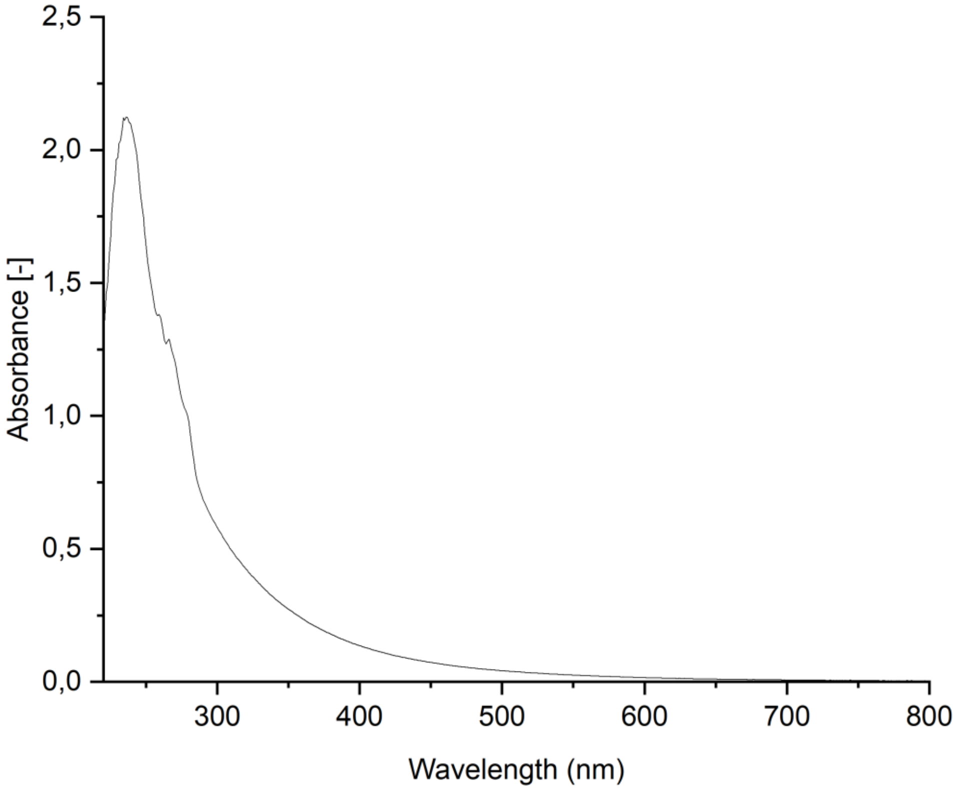

Figure S47: UV-Vis spectrum of $[Zr(GaTMP)_8](BAr^F)_2$ (**5**) in THF.

9. Magnetic Measurements

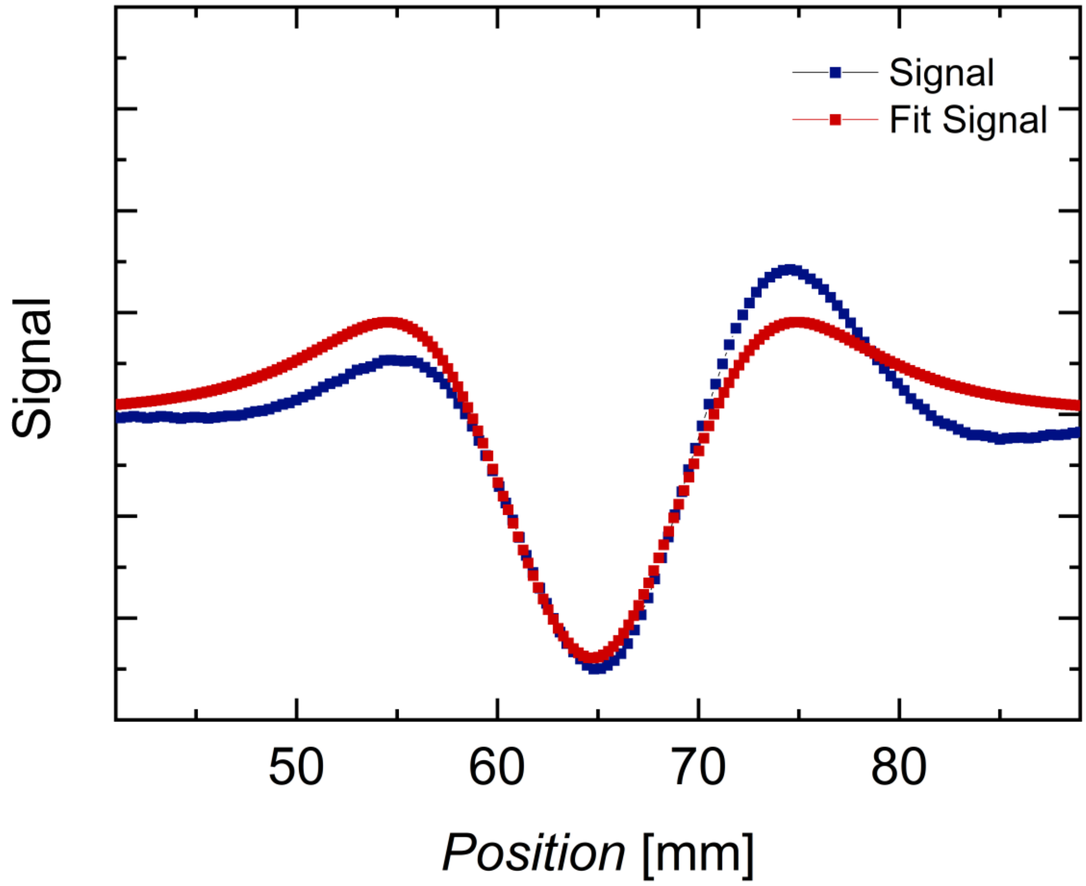

Figure S48: Centering of respective samples in a constant field of H_c = 1 T at a Temperature of 300K with a resulting diamagnetic signal for the compound **5**.

10. Crystallography

[Ti(GaTMP)$_7$] (**1**)

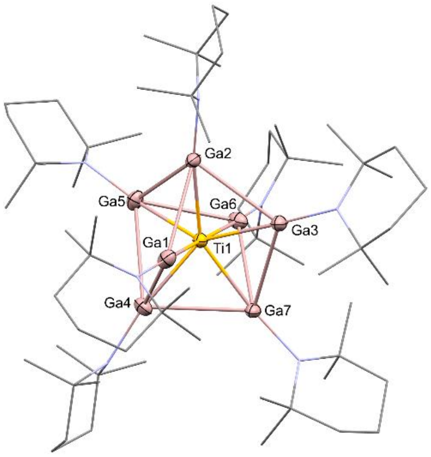

CCDC number	2484315
Empirical formula	$C_{63}H_{126}Ga_7N_7Ti$
Formula weight	1517.64
Temperature [K]	100(2)
Crystal system	monoclinic
Space group (number)	Cc (9)
a [Å]	15.2706(8)
b [Å]	21.8702(12)
c [Å]	23.1045(12)
α [°]	90
β [°]	109.273(2)
γ [°]	90
Volume [Å^3]	7283.8(7)
Z	4
ρ_{calc} [gcm^{-3}]	1.384
μ [mm^{-1}]	2.691
F(000)	3168
Crystal size [mm^3]	0.129×0.142×0.215
Crystal colour	orange
Crystal shape	block
Radiation	MoK_α (λ=0.71073 Å)
2θ range [°]	3.72 to 52.79 (0.80 Å)
Index ranges	−19 ≤ h ≤ 19<br>−27 ≤ k ≤ 27<br>−28 ≤ l ≤ 28
Reflections collected	247717
Independent reflections	14879<br>R_{int} = 0.0610<br>R_{sigma} = 0.0206
Completeness to θ = 25.242°	99.9 %
Data / Restraints / Parameters	14879 / 5626 / 1381
Goodness-of-fit on F^2	1.035
Final *R* indexes [$I \geq 2\sigma(I)$]	R_1 = 0.0252<br>wR_2 = 0.0628
Final *R* indexes [all data]	R_1 = 0.0308<br>wR_2 = 0.0655
Largest peak/hole [eÅ^{-3}]	0.98/−0.64
Flack X parameter	–0.008(2)

[Zr(GaTMP)$_7$] (**2**)

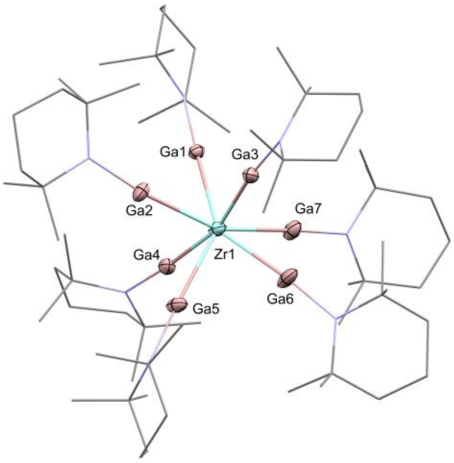

CCDC number	2484316
Empirical formula	$C_{63}H_{126}Ga_7N_7Zr$
Formula weight	1560.96
Temperature [K]	100(2)
Crystal system	triclinic
Space group (number)	$P\overline{1}$ (2)
a [Å]	12.5960(10)
b [Å]	12.7436(9)
c [Å]	24.9845(19)
α [°]	100.625(3)
β [°]	97.016(3)
γ [°]	103.616(2)
Volume [Å^3]	3773.0(5)
Z	2
ρ_{calc} [gcm^{-3}]	1.374
μ [mm^{-1}]	2.630
F(000)	1620
Crystal size [mm^3]	0.075×0.133×0.174
Crystal colour	orange
Crystal shape	rod
Radiation	MoK_α (λ=0.71073 Å)
2θ range [°]	4.03 to 52.83 (0.80 Å)
Index ranges	−15 ≤ h ≤ 15<br>−15 ≤ k ≤ 15<br>0 ≤ l ≤ 31
Reflections collected	15720
Independent reflections	15720<br>R_{int} = 0.088<br>R_{sigma} = 0.0522
Completeness to θ = 25.242°	98.9
Data / Restraints / Parameters	15720 / 1714 / 1212
Goodness-of-fit on F^2	1.219
Final *R* indexes [I≥2σ(I)]	R_1 = 0.0556<br>wR_2 = 0.1139
Final *R* indexes [all data]	R_1 = 0.1156<br>wR_2 = 0.1622
Largest peak/hole [eÅ^{-3}]	1.09/−1.14

Compound **2** was refined as a two-component twin against hklf5 data.

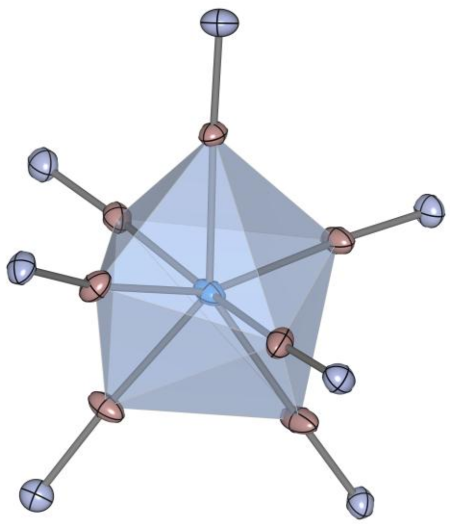

Figure S49: Coordination polyhedron calculated from crystal structure data for and [Zr(GaTMP)$_7$] (**2**).

[Zr(GaTMP)$_8$][BAr$^F{}_4$]$_2$ (**5**)

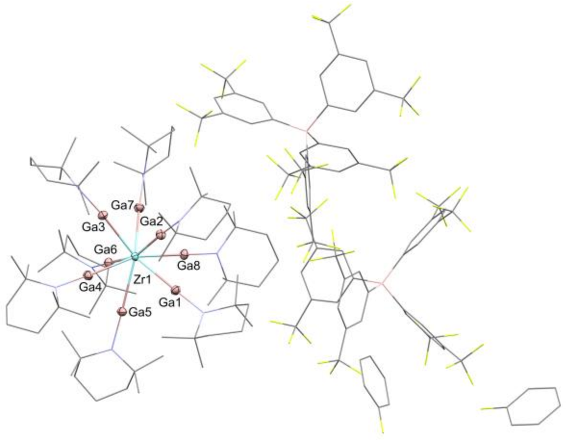

CCDC number	2484317
Empirical formula	$C_{290}H_{351}B_4F_{99}Ga_{16}N_{16}Zr_2$
Formula weight	7283.05
Temperature [K]	100(2)
Crystal system	monoclinic
Space group (number)	$P2_1/n$ (14)
a [Å]	17.7049(10)
b [Å]	32.5007(18)
c [Å]	28.0612(15)
α [°]	90
β [°]	92.954(2)
γ [°]	90
Volume [Å^3]	16125.6(15)
Z	2
ρ_{calc} [gcm^{-3}]	1.500
μ [mm^{-1}]	1.483
F(000)	7380
Crystal size [mm^3]	0.247×0.278×0.325
Crystal colour	orange
Crystal shape	block
Radiation	MoK_α (λ=0.71073 Å)
2θ range [°]	3.83 to 56.57 (0.75 Å)
Index ranges	−23 ≤ h ≤ 23<br>−43 ≤ k ≤ 43<br>−37 ≤ l ≤ 37
Reflections collected	439682
Independent reflections	40040<br>R_{int} = 0.0769<br>R_{sigma} = 0.0342
Completeness to θ = 25.242°	100.0
Data / Restraints / Parameters	40040 / 1564 / 2166
Goodness-of-fit on F^2	1.247
Final *R* indexes [I≥2σ(I)]	R_1 = 0.0513<br>wR_2 = 0.1251
Final *R* indexes [all data]	R_1 = 0.0951<br>wR_2 = 0.1680
Largest peak/hole [eÅ^{-3}]	1.41/−1.18

11. Continuous Shape Measure

Table S1: Summary of Continuous shape measures for compounds **1** and **2**.

	$S_Q(P)$ vs. idealized mono-capped trigonal prismatic structure	$S_Q(P)$ vs. $[ZrF_7]^{3-}$	$S_Q(P)$ vs. $[V(CN)_7]^{4-}$
$[Ti(GaTMP)_7]$ (**1**)	3.27	2.44	3.33
$[Zr(GaTMP)_7]$ (**2**)	2.21	1.53	4.53

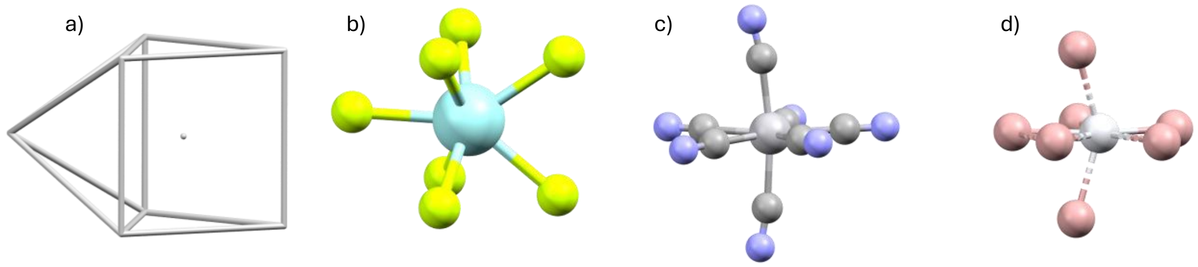

Figure S50: Geometries of a) the idealized mono-capped trigonal prismatic structure, b) the $[ZrF_7]^{3-}$ anion, c) the $[V(CN)_7]^{4-}$ anion and d) $TiGa_7$ motif extracted from the Ti_5Ga_4 phase.

12. Computational Results

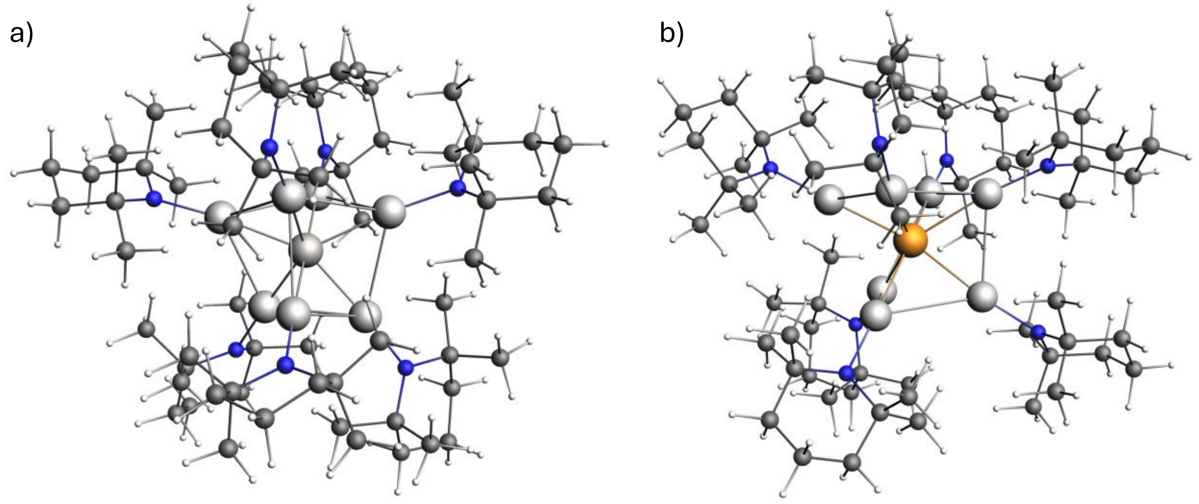

Figure S51: DFT optimized structures of a) [Ti(GaTMP)$_7$] (**1**) and b) [Zr(GaTMP)$_7$] (**2**). The structures were optimized at eh PBE0/TZ2P-D3 level of theory.

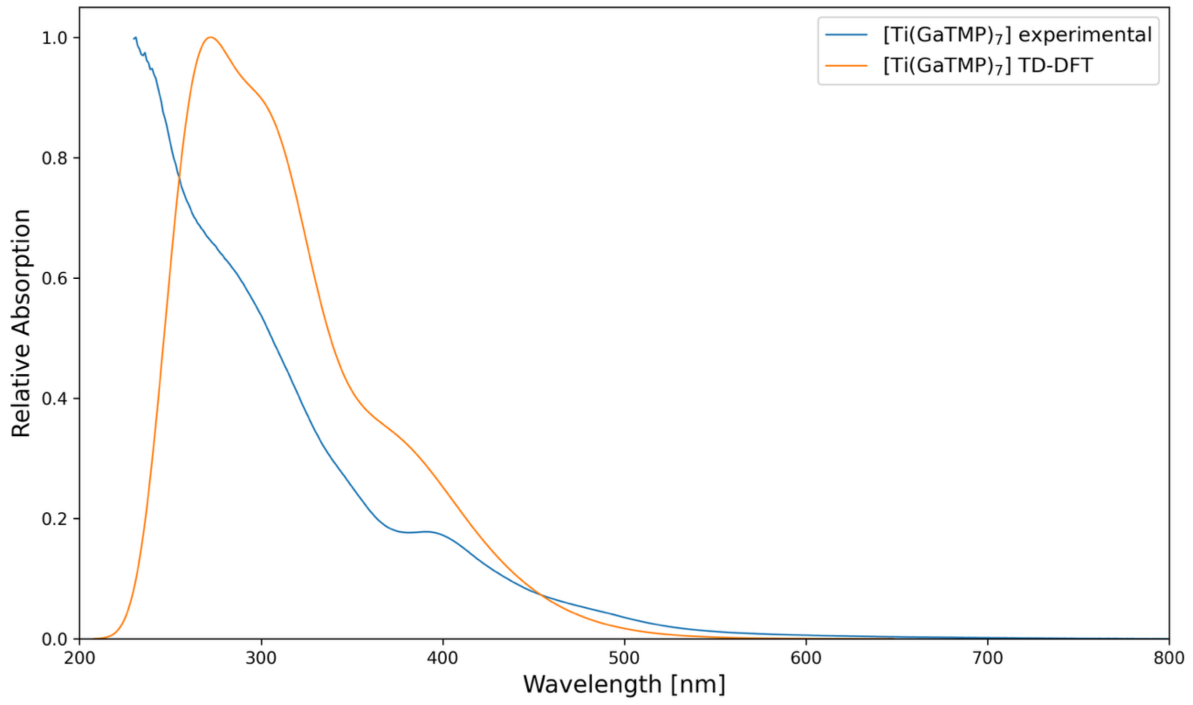

Figure S52: TD-DFT simulated and experimental UV-Vis spectra of [Ti(GaTMP)$_7$] (**1**). The low-energy shoulder computed at ~ 380 nm is of MLCT (Ti→Ga) nature, whereas the high-energy peak at ~ 300nm is dominated by LLCT (Ga→Ga) transitions.

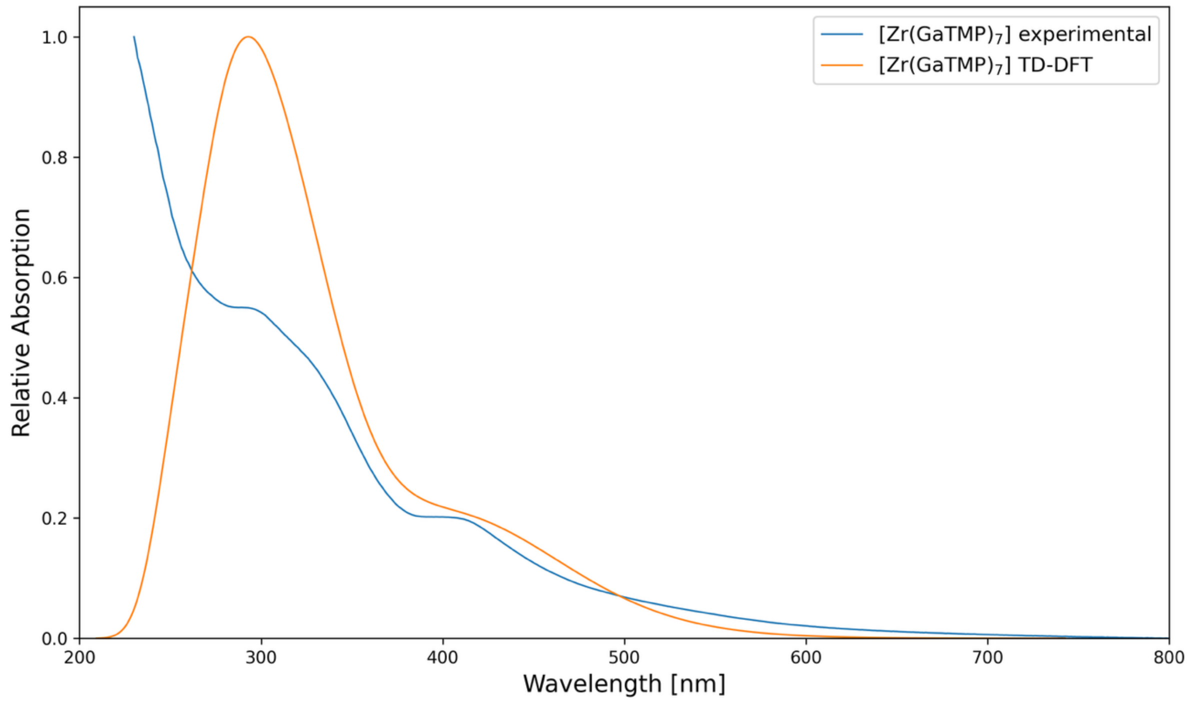

Figure S53: TD-DFT simulated and experimental UV-Vis spectra of [Zr(GaTMP)$_7$] (**2**). The low-energy shoulder computed at ~ 420 nm is of MLCT (Zr→Ga) nature, whereas the high-energy peak at ~ 300nm is dominated by LLCT (Ga→Ga) transitions.

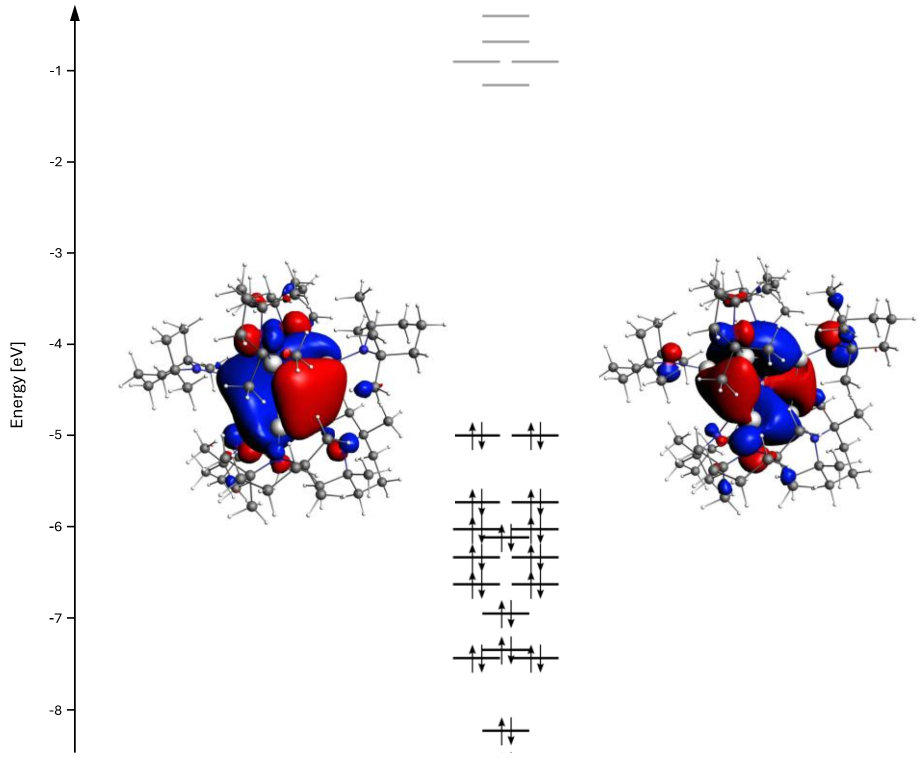

Figure S54: Kohn-Sham frontier orbitals of [Ti(GaTMP)$_7$] (**1**). The HOMO and HOMO-1 show the π-backbonding into bonding combinations of 4p atomic orbitals of gallium.

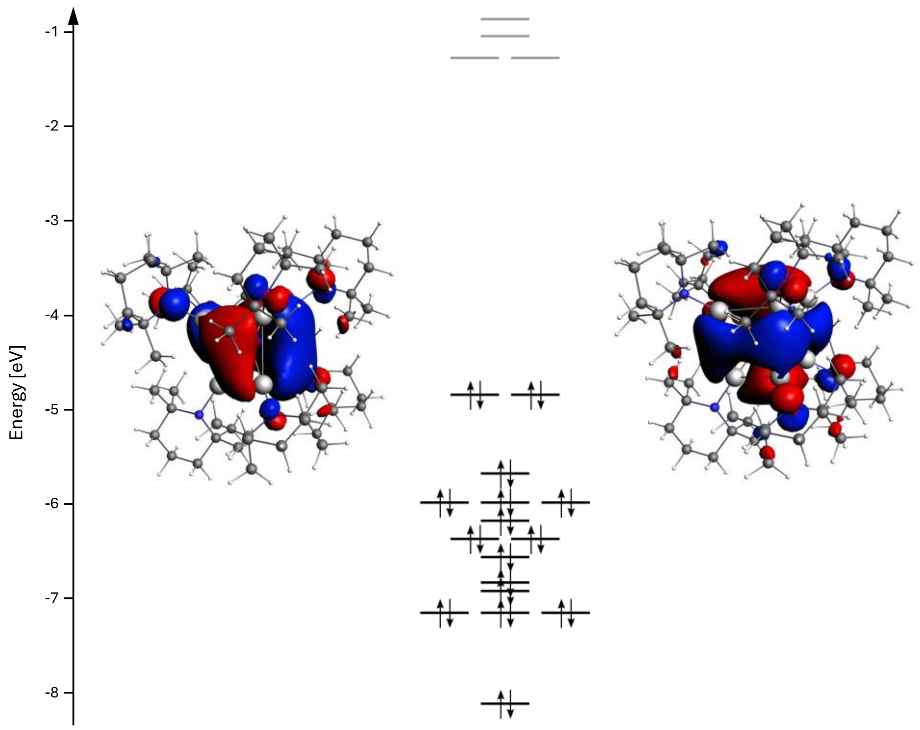

Figure S55: Kohn-Sham frontier orbitals of $[Zr(GaTMP)_7]$ (**2**). The HOMO and HOMO-1 show the π-backbonding into bonding combinations of 4p atomic orbitals of gallium.

Table S2: Morokuma-Ziegler Energy Decomposition Analysis of $[Ti(GaTMP)_7]$ (**1**), $[Zr(GaTMP)_7]$ (**2**) and $[Zr(GaTMP)_8]^{2+}$ (**5**) as well as the theoretical structure of $[Hf(GaTMP)_7]$ (**3**), $[Ti(GaTMP)_8]^{2+}$ (**4**) and $[Hf(GaTMP)_8]^{2+}$ (**6**). The total bonding energy (TBE) is the sum of four components: E_{Pauli} (Pauli repulsion), E_{elstat} (electrostatic interaction), E_{orb} (orbital interaction) and E_{disp} (dispersion forces). All values in eV

Fragmentation	Ti + $(GaTMP)_7$	Zr + $(GaTMP)_7$	Hf + $(GaTMP)_7$	Ti^{2+} + $(GaTMP)_8$	Zr^{2+} + $(GaTMP)_8$	Hf^{2+} + $(GaTMP)_8$
E_{pauli}	65.19	70.87	75.49	18.41	27.01	34.64
E_{elstat}	-29.03	-30.14	-31.98	-7.17	-10.20	-17.35
E_{orb}	-50.47	-55.26	-57.87	-34.84	-40.42	-40.75
E_{disp}	-0.49	-0.24	-0.26	-0.55	-0.58	-0.55
TBE	-14.79	-14.76	-14.63	-24.15	-24.18	-24.01

Table S3: Averaged computed NBO and QTAIM atom charges for the DFT optimized structures of $[Ti(GaTMP)_7]$ (**1**), $[Zr(GaTMP)_7]$ (**2**) and $[Zr(GaTMP)_8]^{2+}$ (**5**) as well as the theoretical structure of $[Hf(GaTMP)_7]$ (**3**), $[Ti(GaTMP)_8]^{2+}$ (**4**) and $[Hf(GaTMP)_8]^{2+}$ (**6**).

		NBO Charges	QTAIM Charges
$[Ti(GaTMP)_7]$ (**1**)	Ti	-4.00	0.89
	Ga	1.18	0.44
	N	-1.04	-1.19
$[Zr(GaTMP)_7]$ (**2**)	Zr	-4.25	0.95
	Ga	1.23	0.44
	N	-1.05	-1.19
$[Hf(GaTMP)_7]$ (**3**)	Hf	-4.22	0.99
	Ga	1.22	0.43
	N	-1.05	-1.20
$[Ti(GaTMP)_8]^{2+}$ (**4**)	Ti	-1.24	1.03
	Ga	-0.39	0.55
	N	-0.44	-1.19
	Zr	-2.09	1.08

$[Zr(GaTMP)_8]^{2+}$ (**5**)	Ga	-1.10	0.55
	N	0.37	-1.19
$[Hf(GaTMP)_8]^{2+}$ (**6**)	Hf	0.71	1.14
	Ga	-1.23	0.54
	N	0.67	-1.19

Table S4: Averaged computed Ga···Ga and Ga-N Wiberg bond indices and QTAIM delocalization indices for the $(GaTMP)_{7/8}$ shell extracted from the DFT optimized structures of $[Ti(GaTMP)_7]$ (**1**), $[Zr(GaTMP)_7]$ (**2**) and $[Zr(GaTMP)_8]^{2+}$ (**5**) as well as the theoretical structure of $[Hf(GaTMP)_7]$ (**3**), $[Ti(GaTMP)_8]^{2+}$ (**4**) and $[Hf(GaTMP)_8]^{2+}$ (**6**).

		Wiberg-Bond-Indices	QTAIM Delocalization Indices
L_7 from **1**	Ga–N	0.256	0.756
	Ga–Ga	0.353	0.312
L_7 from **2**	Ga–N	0.240	0.773
	Ga–Ga	0.347	0.284
L_7 from **3**	Ga–N	0.235	0.771
	Ga–Ga	0.354	0.301
L_8 from **4**	Ga–N	0.445	0.755
	Ga–Ga	0.665	0.442
L_8 from **5**	Ga–N	0.646	0.769
	Ga–Ga	0.632	0.399
L_8 from **6**	Ga–N	0.439	0.768
	Ga–Ga	0.561	0.404

Table S5: Averaged computed NBO and QTAIM atom charges for the $(GaTMP)_{7/8}$ shell extracted from the DFT optimized structures of $[Ti(GaTMP)_7]$ (**1**), $[Zr(GaTMP)_7]$ (**2**) and $[Zr(GaTMP)_8]^{2+}$ (**5**) as well as the theoretical structure of $[Hf(GaTMP)_7]$ (**3**), $[Ti(GaTMP)_8]^{2+}$ (**4**) and $[Hf(GaTMP)_8]^{2+}$ (**6**).

		NBO Charges	QTAIM Charges
L_7 from **1**	Ga	0.67	0.57
	N	-1.09	-1.18
L_7 from **2**	Ga	0.70	0.58
	N	-1.12	-1.18
L_7 from **3**	Ga	0.70	0.58
	N	-1.12	-1.18
L_8 from 4	Ga	-1.11	0.57
	N	0.43	-1.17
L_8 from **5**	Ga	-0.95	0.58
	N	0.30	-1.17
L_8 from **6**	Ga	-1.36	0.58
	N	0.50	-1.17

Table S6: Averaged topological Ga···Ga BCP descriptors in $[Ti(GaTMP)_7]$ (**1**), $[Zr(GaTMP)_7]$ (**2**) and $[Zr(GaTMP)_8]^{2+}$ (**5**), the theoretical structure of $[Hf(GaTMP)_7]$ (**3**), $[Ti(GaTMP)_8]^{2+}$ (**4**) and $[Hf(GaTMP)_8]^{2+}$ (**6**) as well as in their respective extracted L_7 coordination spheres. ρ, $\nabla^2\rho$, H, V and G are the electron density, Laplacian of ρ density, energy density, potential energy density and kinetic energy density values at the bcp, respectively. All values in a.u.

BCP	$[Ti(GaTMP)_7]$ (**1**) 3 x Ga–Ga	L_7 from **1** 11 x Ga–Ga	$[Zr(GaTMP)_7]$ (**1**) 2 x Ga–Ga	L_7 from **2** 11 x Ga–Ga	$[Hf(GaTMP)_7]$ (**3**) 0 x Ga–Ga	L_7 from **3** 11 x Ga–Ga
ρ	0.041	0.028	0.038	0.021	n/a	0.022
$\nabla^2\rho$	0.016	0.008	0.015	0.010	n/a	0.013
H	-0.013	-0.007	-0.011	-0.004	n/a	-0.008
V	-0.030	-0.016	-0.026	-0.011	n/a	-0.011
|*V*|/ *G*	1.766	1.712	1.751	1.547	n/a	1.575

BCP	$[Ti(GaTMP)_8]^{2+}$ (**4**) 10 x Ga–Ga	L_7 from **4** 10 x Ga–Ga	$[Zr(GaTMP)_8]^{2+}$ (**5**) 9 x Ga–Ga	L_7 from **5** 10 x Ga–Ga	$[Hf(GaTMP)_8]^{2+}$ (**6**) 9 x Ga–Ga	L_7 from **6** 10 x Ga–Ga

ρ	0.040	0.037	0.036	0.031	0.037	0.031
$\nabla^2\rho$	0.009	0.002	0.013	0.005	0.013	0.004
H	-0.013	-0.012	-0.010	-0.008	-0.011	-0.009
V	-0.028	-0.024	-0.023	-0.018	-0.025	-0.018
$\|V\|/G$	1.847	1.953	1.757	1.869	1.760	1.888

Table S7: Morokuma-Ziegler Energy Decomposition Analysis of three $(GaTMP)_2$ fragments extracted out of the optimized structure of $[Ti(GaTMP)_7]$ (**1**). The total bonding energy (TBE) is the sum of four components: E_{Pauli} (Pauli repulsion), E_{elstat} (electrostatic interaction), E_{orb} (orbital interaction) and E_{disp} (dispersion forces). All values in eV.

Fragmentation	TMPGa + GaTMP Ga2–Ga3 (2.837 Å)	TMPGa + GaTMP Ga4–Ga7 (2.905 Å)	TMPGa + GaTMP Ga3–Ga6 (3.343 Å)
E_{pauli}	2.47	2.10	0.87
E_{elstat}	-1.34	-1.14	-0.40
E_{orb}	-0.90	-0.76	-0.32
E_{disp}	-0.22	-0.20	-0.16
TBE	0.00	0.00	-0.02

Table S8: Ga···Ga Wiberg bond indices in three different $(GaTMP)_2$ fragments with different Ga-Ga distances extracted from the optimized structure of $[Ti(GaTMP)_7]$ (**1**) and within the optimized structure of $[Ti(GaTMP)_7]$ (**1**).

		Distance [Å]	Wiberg-Bond-Indices
Ga(2)-Ga(3)	$(GaTMP)_2$	2.837	0.597
	$[Ti(GaTMP)_7]$ (**1**)		0.305
Ga(4)-Ga(7)	$(GaTMP)_2$	2.905	0.558
	$[Ti(GaTMP)_7]$ (**1**)		0.296
Ga(3)-Ga(6)	$(GaTMP)_2$	3.343	0.331
	$[Ti(GaTMP)_7]$ (**1**)		0.179

Table S9: Averaged computed distances for $[Ti(CO)_7]$, $[Zr(CO)_7]$, $[Hf(CO)_7]$, $[Ti(CO)_8]^{2+}$, $[Zr(CO)_8]^{2+}$ and $[Hf(CO)_8]^{2+}$ as well as the corresponding averaged Wiberg bond indices and QTAIM delocalization indices.

		DFT-Optimized Distances	Wiberg-Bond-Indices	QTAIM Delocalization Indices
$[Ti(CO)_7]$	Ti–C	2.096	0.721	0.526
	C–C	2.647	0.076	0.150
$[Zr(CO)_7]$	Zr–C	2.274	0.689	0.515
	C–C	2.803	0.065	0.129
$[Hf(CO)_7]$	Hf–C	2.232	0.708	0.525
	C–C	2.701	0.071	0.147
$[Ti(CO)_8]^{2+}$	Ti–C	2.235	0.611	0.408
	C–C	2.621	0.052	0.115
$[Zr(CO)_8]^{2+}$	Zr–C	2.387	0.574	0.408
	C–C	2.768	0.035	0.093
$[Hf(CO)_8]^{2+}$	Hf–C	2.343	0.596	0.418
	C–C	2.725	0.041	0.103

Table S10: Morokuma-Ziegler energy decomposition analysis of $[Ti(CO)_7]$, $[Zr(CO)_7]$, $[Hf(CO)_7]$, $[Ti(CO)_8]^{2+}$, $[Zr(CO)_8]^{2+}$ and $[Hf(CO)_8]^{2+}$. The total bonding energy (TBE) is the sum of four components: E_{Pauli} (Pauli repulsion), E_{elstat} (electrostatic interaction), E_{orb} (orbital interaction) and E_{disp} (dispersion forces). All values in eV.

Fragmentation	Ti + $(CO)_7$	Zr + $(CO)_7$	Hf + $(CO)_7$
E_{pauli}	67.33	66.29	76.69
E_{elstat}	-23.15	-22.26	-24.76
E_{orb}	-59.25	-58.18	-66.21
E_{disp}	-0.12	-0.14	-0.11

TBE	-15.18	-14.29	-14.39

Fragmentation	Ti^{2+} + $(CO)_8$	Zr^{2+} + $(CO)_8$	Hf^{2+} + $(CO)_8$
E_{pauli}	18.12	23.71	34.54
E_{elstat}	-7.02	-8.48	-14.97
E_{orb}	-27.93	-31.19	-35.79
E_{disp}	-0.12	-0.14	-0.11
TBE	-16.95	-16.11	-16.33

13. References

1. I. Chávez, A. Alvarez-Carena, E. Molins, A. Roig, W. Maniukiewicz, A. Arancibia, V. Arancibia, H. Brand and J. Manuel Manríquez, Selective oxidants for organometallic compounds containing a stabilising anion of highly reactive cations: (3,5(CF3)2C6H3)4B−)Cp2Fe+ and (3,5(CF3)2C6H3)4B−)Cp*2Fe+, *J. Organomet. Chem.*, 2000, **601**, 126-132.
2. M. Muhr, P. Heiß, M. Schütz, R. Bühler, C. Gemel, M. H. Linden, H. B. Linden and R. A. Fischer, Enabling LIFDI-MS measurements of highly air sensitive organometallic compounds: a combined MS/glovebox technique, *Dalton Trans.*, 2021, **50**, 9031-9036.
3. *APEX4 Suite of Crystallographic Software*, Bruker AXS Inc., Madison, Wisconsin, USA, Version 2021-10.0 edn., 2021.
4. *Bruker, SAINT*, Bruker AXS Inc., Madison, Wisconsin, USA, V8.40B edn., 2021.
5. *SADABS*, Bruker AXS Inc., Madison, Wisconsin, USA, Version 2021 edn., 2021.
6. *TWINABS*, Bruker AXS Inc., Madison, Wisconsin, USA, Version 2012/1 edn., 2021.
7. C. B. Hübschle, G. M. Sheldrick and B. Dittrich, ShelXle: a Qt graphical user interface for SHELXL, *J. Appl. Cryst.*, 2011, **44**, 1281-1284.
8. G. Sheldrick, SHELXT - Integrated space-group and crystal-structure determination, *Acta Crystallogr. A*, 2015, **71**, 3-8.
9. G. M. Sheldrick, *Acta Crystallogr. C*, 2015, **71**, 3–8.
10. *International Tables for Crystallography Volume C, Mathematical, Physical and Chemical Tables*, International Union of Crystallography, Chester, England, 2006.
11. D. Kratzert, J. J. Holstein and I. Krossing, *Acta Crystallogr. C*, 2015, **48**, 933–938.
12. A. Spek, *Acta Crystallogr. C*, 2015, **71**, 9-18.
13. C. F. Macrae, I. J. Bruno, J. A. Chisholm, P. R. Edgington, P. McCabe, E. Pidcock, L. Rodriguez-Monge, R. Taylor, J. van de Streek and P. A. Wood, Mercury CSD 2.0 - new features for the visualization and investigation of crystal structures, *J. Appl. Cryst.*, 2008, **41**, 466-470.
14. K. Momma and F. Izumi, VESTA 3 for three-dimensional visualization of crystal, volumetric and morphology data, *Journal of Applied Crystallography*, 2011, **44**, 1272-1276.
15. D. Kratzert, *FinalCif, V106, https://dkratzert.de/finalcif.html.*
16. O. Kahn, *Molecular Magnetism*, Wiley-VCH, 1993.
17. G. te Velde, F. M. Bickelhaupt, E. J. Baerends, C. Fonseca Guerra, S. J. A. van Gisbergen, J. G. Snijders and T. Ziegler, Chemistry with ADF, *J. Comput. Chem.*, 2001, **22**, 931-967.
18. E. van Lenthe, E. J. Baerends and J. G. Snijders, Relativistic regular two-component Hamiltonians, *J. Chem. Phys.*, 1993, **99**, 4597-4610.
19. E. van Lenthe, E. J. Baerends and J. G. Snijders, Relativistic total energy using regular approximations, *J. Chem. Phys.*, 1994, **101**, 9783-9792.
20. E. van Lenthe and E. J. Baerends, Optimized Slater-type basis sets for the elements 1–118, *J. Comput. Chem.*, 2003, **24**, 1142-1156.
21. C. Adamo and V. Barone, Toward reliable density functional methods without adjustable parameters: The PBE0 model, *J. Chem. Phys.*, 1999, **110**, 6158-6170.
22. J. P. Perdew, K. Burke and M. Ernzerhof, Generalized Gradient Approximation Made Simple, *Phys. Rev. Lett.*, 1996, **77**, 3865-3868.
23. J. P. Perdew, K. Burke and M. Ernzerhof, *Phys. Rev. Lett.*, 1997, **78**, 1396.
24. A. D. Becke, Density-functional exchange-energy approximation with correct asymptotic behavior, *Phys. Rev. A*, 1988, **38**, 3098-3100.
25. J. P. Perdew, Density-functional approximation for the correlation energy of the inhomogeneous electron gas., *Phys. Rev. B*, 1986, **33**, 8822-8824.
26. S. Grimme, Semiempirical GGA-type density functional constructed with a long-range dispersion correction, *J. Comput. Chem.*, 2006, **27**, 1787-1799.
27. E. D. Glendening, J. K. Badenhoop, A. E. Reed, J. E. Carpenter, J. A. Bohmann, C. M. Morales and F. Weinhold.*Journal*.

28. R. F. W. Bader, *Atoms in Molecules-A Quantum Theory*, Oxford University Press, Oxford, England, 1990.
29. J. I. Rodríguez, An efficient method for computing the QTAIM topology of a scalar field: The electron density case, *J. Comput. Chem.*, 2013, **34**, 681-686.
30. J. I. Rodríguez, R. F. W. Bader, P. W. Ayers, C. Michel, A. W. Götz and C. Bo, A high performance grid-based algorithm for computing QTAIM properties, *Chem. Phys. Lett.*, 2009, **472**, 149-152.
31. F. M. Bickelhaupt and E. J. Baerends, *Rev. Comput. Chem.* , Wiley, New York 2000.
32. K. Morokuma, Molecular Orbital Studies of Hydrogen Bonds. III. C=O···H–O Hydrogen Bond in H2CO···H2O and H2CO···2H2O, *J. Chem. Phys.*, 1971, **55**, 1236-1244.
33. T. Ziegler and A. Rauk, A theoretical study of the ethylene-metal bond in complexes between copper(1+), silver(1+), gold(1+), platinum(0) or platinum(2+) and ethylene, based on the Hartree-Fock-Slater transition-state method, *Inorg. Chem.*, 1979, **18**, 1558-1565.
34. M. J. Frisch, G. W. Trucks, H. B. Schlegel, G. E. Scuseria, M. A. Robb, J. R. Cheeseman, G. Scalmani, V. Barone, G. A. Petersson, H. Nakatsuji, X. Li, M. Caricato, A. V. Marenich, J. Bloino, B. G. Janesko, R. Gomperts, B. Mennucci, H. P. Hratchian, J. V. Ortiz, A. F. Izmaylov, J. L. Sonnenberg, Williams, F. Ding, F. Lipparini, F. Egidi, J. Goings, B. Peng, A. Petrone, T. Henderson, D. Ranasinghe, V. G. Zakrzewski, J. Gao, N. Rega, G. Zheng, W. Liang, M. Hada, M. Ehara, K. Toyota, R. Fukuda, J. Hasegawa, M. Ishida, T. Nakajima, Y. Honda, O. Kitao, H. Nakai, T. Vreven, K. Throssell, J. A. Montgomery Jr., J. E. Peralta, F. Ogliaro, M. J. Bearpark, J. J. Heyd, E. N. Brothers, K. N. Kudin, V. N. Staroverov, T. A. Keith, R. Kobayashi, J. Normand, K. Raghavachari, A. P. Rendell, J. C. Burant, S. S. Iyengar, J. Tomasi, M. Cossi, J. M. Millam, M. Klene, C. Adamo, R. Cammi, J. W. Ochterski, R. L. Martin, K. Morokuma, O. Farkas, J. B. Foresman and D. J. Fox.*Journal*, 2016.
35. F. Weigend, Accurate Coulomb-fitting basis sets for H to Rn, *Phys. Chem. Chem. Phys.*, 2006, **8**, 1057-1065.
36. F. Weigend and R. Ahlrichs, Balanced basis sets of split valence, triple zeta valence and quadruple zeta valence quality for H to Rn: Design and assessment of accuracy, *Phys. Chem. Chem. Phys.*, 2005, **7**, 3297-3305.
37. M. Molon, C. Gemel, M. von Hopffgarten, G. Frenking and R. A. Fischer, Molecular Hume–Rothery Compounds [M(ZnR)n] and [M(ZnR)a(GaR)b] (a + 2b = n ≥ 8): Relations of Coordination Polyhedra and Electronic Structure, *Inorg. Chem.*, 2011, **50**, 12296-12302.
38. M. A. Saada, A. Hémon-Ribaud, M. Leblanc and V. Maisonneuve, Conservation of [(C2H4NH3)3N]2·(ZrF7)2·H2O layers during the topotactic dehydration of [(C2H4NH3)3N]2·(ZrF7)2·9H2O, *J. Fluor. Chem.*, 2005, **126**, 1072-1077.
39. R. A. Levenson and R. L. R. Towns, Crystal and molecular structure of potassium heptocyanovanadate(III) dihydrate, *Inorg. Chem.*, 1974, **13**, 105-109.
40. K. Schubert, H. G. Meissner, M. Pötzschke, W. Rossteutscher and E. Stolz, Einige Strukturdaten metallischer Phasen (7), *Naturwissenschaften*, 1962, **49**, 57-57.
41. M. Muhr, H. Liang, L. Allmendinger, R. Bühler, F. E. Napoli, D. Ukaj, M. Cokoja, C. Jandl, S. Kahlal, J.-Y. Saillard, C. Gemel and R. A. Fischer, Catalytic Alkyne Semihydrogenation with Polyhydride Ni/Ga Clusters, *Angew. Chem. Int. Ed.*, 2023, **62**, e202308790.
42. M. F. Lappert, M. J. Slade, A. Singh, J. L. Atwood, R. D. Rogers and R. Shakir, Structure and reactivity of sterically hindered lithium amides and their diethyl etherates: crystal and molecular structures of [Li{N(SiMe3)2}(OEt2)]2 and tetrakis(2,2,6,6-tetramethylpiperidinatolithium), *J. Am. Chem. Soc.*, 1983, **105**, 302-304.
43. P. L. Hall, J. H. Gilchrist, A. T. Harrison, D. J. Fuller and D. B. Collum, Mixed aggregation of lithium enolates and lithium halides with lithium 2,2,6,6-tetramethylpiperidide (LiTMP), *Journal of the American Chemical Society*, 1991, **113**, 9575-9585.
44. P. Binger, G. Benedikt, G. W. Rotermund and R. Köster, Borverbindungen, XV. Alkalimetall-alkyl-1-alkinyl-boranate, *Justus Liebigs Ann. Chem.*, 1968, **717**, 21-40.

45. H. Bönnemann and B. Korall, Ether-Soluble Ti0 and Bis(η6-arene)titanium(0) Complexes from the Reduction of TiCl4 with Triethylhydroborate, *Angew. Chem. Int. Ed. Engl.*, 1992, **31**, 1490-1492.
46. J. C. Green, M. L. H. Green and N. M. Walker, Synthesis, crystal structure and reactions of zerovalent 16-electron bis(η-cycloheptatriene)zirconium, *J. Chem. Soc., Dalton Trans.*, 1991, DOI: 10.1039/DT9910000173, 173-180.
